# Conversational repairs on Reddit: Widely initiated but often uncompleted

**DOI:** 10.1371/journal.pone.0316618

**Published:** 2025-01-02

**Authors:** Alex Goddard, Alex Gillespie

**Affiliations:** 1 Department of Psychological and Behavioural Science, London School of Economics, London, United Kingdom; 2 Department of Psychology, Oslo New University College, Oslo, Norway; Kitami Institute of Technology, JAPAN

## Abstract

Conversational repair has been proposed as a universal system for maintaining mutual understanding during social interactions. The repair system has been studied extensively in offline synchronous interactions (e.g., face-to-face, phone calls) and has been observed across cultures and languages. However, the prevalence of conversational repairs is unclear in online asynchronous text-based interactions. Online interactions are increasingly important for public deliberation, and it is therefore important to understand how conversational repairs manifest in different online contexts. To address this gap, we conducted two analyses of Other-initiated repairs in 25 English-language Reddit communities (subreddits), covering a diverse range of topics and communication norms. Analysis 1 examines the frequency of repair initiations across subreddits, finding them to be widespread (in every subreddit) and frequent (58.48% of interactions experience a repair initiation). Analysis 2 examines the emergence of repairs, finding that a repair initiation becomes increasingly likely the longer a comments thread progresses (Median time-to-repair = 6 turns). These results suggest that the prevalence and emergence of repair initiations in online interactions are comparable to offline contexts. However, we also find 44.80% of initiations receive no reply, precluding the possibility of a repair completion. Thus, conservatively, nearly half of the repair initiations in our data went uncompleted. This suggests that the online medium alters the way initiations are completed compared to offline interactions. We discuss the implications of this finding and avenues for future research.

## Introduction

Conversational repairs, defined as any sequence of interaction aimed at addressing misunderstanding [1], have been observed across cultures and language groups with little variation in functional form [2, 3]. Currently, comparative studies of repair have been limited to synchronous (generally face-to-face) communication and virtual, public, asynchronous, and text-based interactions have been neglected. Yet, these online interactions (e.g., comments sections, blog posts, Twitter threads) influence democracies by shaping public deliberation [4, 5]. Conversational repairs may be especially important in online interactions [6], which are frequently noted for their incivility [7, 8], as they are the way interlocuters address emergent misunderstandings.

To address this gap, our research examined the distribution (Analysis 1) and emergence (Analysis 2) of Other-initiations of repair across 25 subreddits (3,750 interactions and 157,667 comments), each with their own norms and modalities [9]. We find repair initiations are widespread (present in all 25 subreddits), frequent (found in 58.49% of interactions) and that the likelihood of a comments thread experiencing an initiation increases alongside its length (> 50% at the 6^th^ turn). These results resemble those found in offline synchronous interactions (e.g., face-to-face), supporting the idea of a common conversational repair system across mediums. However, due to the small number of turns in the average comment threads (M = 3.60; SD = 2.03), many repair initiations lacked any response (44.80%) and, therefore, a completion. This indicates that, while conversational repairs are initiated in a similar way in online and offline interactions, they differ in how they are completed.

### Universal system of repair in face-to-face interactions

A social interaction is defined here as two or more individuals (Self and Other), embedded in a context, interacting over any topic (Object), using a semiotic system such as language [10]. Language is not a conduit, meaning information is not simply transferred between interlocuters [11]. Instead, meaning is co-constructed through interaction and the same word can express different things in different contexts [12]. Moreover, language can be interpreted in many different ways [13]. Misunderstanding and miscommunication are therefore unavoidable in social interaction, as participants must negotiate the various meanings, intentions, and interpretations of language across different contexts [14].

Conversational repair has been suggested as a universal system for addressing misunderstanding and miscommunication during social interaction [2, 15–17]. Repairs originate in Conversation Analysis (CA), a field of study dedicated to studying naturally occurring social interactions [18]. In practice, this typically involves transcribing recordings of synchronous interactions (typically video and audio recordings) and performing rigorous qualitative analysis [19]. A repair is defined as a sequence of actions performed to address potential or emergent trouble in social interaction [1, 20]. Any repair sequence involves three steps: a “trouble source”, an “initiation”, and a “completion”. A trouble source was originally defined as any problem of “speaking, listening, and understanding” [1], although more recent conceptualizations also point to repair being used for maintaining social norms during interaction [17]. An initiation draws attention to the trouble source (e.g., “what did you mean by X?”) and a completion attempts to rectify the trouble source (e.g., “I meant Y”).

Repairs are typologized by who (Self or Other) initiated and completed the sequence, where the trouble source is always produced by the Self. Table 1 shows the five repair sequences that are consistently found across the literature [1, 15, 16, 21, 22]. Self-repairs are initiated and completed by the Self in the same turn as the trouble source (turn 1). In asynchronous text-based interactions, this involves typing a comment and editing it repeatedly prior to sending [23]. Third turn repairs [20] are initiated after two turns of trouble: the Self says something (turn 1) that the Other responds to (turn 2). This response reveals an interpretation from the Other that the Self considers worthy of initiating and completing a repair (turn 3). Other-initiated Self-repairs repairs are initiated by the Other (turn 2) by requesting clarification [3] and completed by the Self in the third turn (turn 3). Finally, Other-completed repairs are both initiated and completed by the Other in the turn directly following the trouble source (turn 2).

**Table 1:** Repair typology.

Repair	Trouble	Initiator	Completion	Example:
Self	Self (turn 1)	Self (turn 1)	Self (turn 1)	*Self*: “I loved Paris… Sorry, London”
Third turn	Self (turn 1) + Other (turn 2)	Self (turn 3)	Self (turn 3)	*Self*: “I loved Paris.” *Other*: “France is a beautiful country.” *Self*: “Sorry, I meant London.”
Other-initiated	Self (turn 1)	Other (turn 2)	Self (turn 3)	*Self*: “I loved Paris.” *Other*: “I thought you went to London?” Self: “Yes, sorry, I meant London.”
Other-completed	Self (turn 1)	Other (turn 2)	Other (turn 2)	*Self*: “I loved Paris.” *Other*: “You were in London, not Paris.”

Repairs have been suggested as a universal system for addressing communication problems. Theoretically, universality is assumed because of the nature of linguistic communication: miscommunications and misunderstandings are inevitable given enough time because language can be interpreted in multiple ways and meaning is co-constructed by interlocuters rather than transferred [14]. Evidence for universality is given in comparative studies of repairs across languages and cultural contexts [2, 15–17].

Early studies found Self-repairs and Other-initiated repairs were more prevalent than other types, indicating a universal “preference” for Self-completed repairs [1]. Preferences are behaviors that are “conversationally or socially appropriate” [24]. The preference for Self-completion has been studied extensively, with studies finding that, on average, Self-completed repairs occur in 16–24% of observed utterances compared to 3–6% for Other-completed repair [25–30].

More recently, Dingemanse and colleagues’ [3] study of Other-initiated repairs across 12 languages (8 language families) provides direct evidence for a universal system of conversational repair in synchronous interaction. They find that Other-initiated repairs are common in all languages (once every 1.4 minutes), that “95% of repair initiations happen within 4.13 minutes of the last one” (p. 4), and that different communication contexts (e.g., eating, socializing) predict different levels of specificity in the initiation. Previous studies have also found that the word “huh?” appears across languages as an Other-initiation of repair [2, 31].

These findings support a “pragmatic universals hypothesis” [32], which predicts that pragmatics (such as turn-taking and repair) vary little across cultures, enabling variation in other areas of communication [3]. Because of the co-construction of meaning in social interaction, repairs are expected in all cultural contexts, regardless of the functional and contextual form of speech (e.g., phonetics, grammars, vocabularies). Even though the norms around repairs may vary (e.g., politeness of seeking clarification), all languages require a repair system to address misunderstandings in interaction.

### Repairs in online interactions

Currently, there are no comparative studies of conversational repairs in virtual communities. Repairs have been studied extensively in synchronous interactions [15], but have historically received less attention in analyses of computer-mediated text-based communication [23, 33]. However, there has been growing interest in text-based repairs due to the recently increased use of artificial intelligence for language analysis. Specifically, chatbots are being used more frequently for various task-driven purposes (e.g., customer service) and repairs appear important for achieving participants’ goals.

For instance, Ashktorab and colleagues [34] found that participants prefer when a banking chatbot allowed for Self-repair by providing options of intended meaning following misunderstandings. Li and colleagues [35] found that participants will occasionally attempt Self-repair (e.g., rephrasing) when they fail to progress in the chatbot interaction. Dippold [36] found that more common Self-repair strategies (e.g., rephrasing) were not as effective as less frequent Self-repair strategies (e.g., restating the aim) for achieving participant goals. However, these human-computer textual communications provide limited insight into repair strategies in public-facing online interactions between human participants.

In addition to chatbot studies, repairs have been studied within private text-based communications. For instance, Meredith and Stokoe [23] examined repairs in interactions conducted over Facebook instant messenger, identifying a “message construction” Self-repair that involves editing messages prior to making them public, unique to text-based communication. In another example, Mostovaia [37] found that participants in German WhatsApp message threads use various types of Other-initiated Self-repair repair in a similar manner to face-to-face interactions [3]. These studies highlight how repairs operate in text-based mediums; however, they do not speak to how they operate in large public forums where participants are often strangers to each other and have less accountability or pressures to reply.

More relevant to our research, a small number of studies have explored how repairs appear in public online interactions [6, 38, 39]. Studying the repair system in online interactions is particularly important because public deliberation increasingly occurs in a virtual public sphere, where citizens from around the world come to discuss a huge variety of topics [40]. The quality of these public-facing interactions matters for society because they are increasingly shaping individual perspectives [41] and mobilizing offline political action [4]; for instance, Facebook interactions were integral for coordinating the Egyptian revolution [42]. Additionally, participants manage their own and others’ behaviors in online forums, providing insights into how humans manage public interactions. For instance, strangers have been found to come together to tackle norm violations online [43] and content moderation is performed largely by individual volunteer members of different communities [44].

Studying how conversational repairs manifest across different online communities can provide insight into how participants negotiate a diversity of interactional goals and norms. For instance, Gordon [38] conducted a case study into 10 posts and comments from health and weight-loss blogs, finding that text-based repair strategies vary based on the goals and norms of the online communities. In addition, the repair system appears important in the dynamics of norm adherence online. For instance, Paakki and colleagues [6] examined conversation breakdowns in online interactions, finding that trolling participants do not respond to repair attempts in an attempt to derail the flow of conversation. Najma [39] demonstrates how repairs are used in Arabic Twitter for the purposes of activism and holding powerful groups to account. These studies of online repair are context specific, focusing on one online community, meaning there has yet to be a systematic comparison of repairs across different virtual public spheres.

We address this gap by comparing repair initiations across different Reddit communities. Describing the online repair system across different virtual public spheres is important for two reasons. First, it provides insights into how the repair system differs between online (public and asynchronous) and offline (private and synchronous) contexts of interaction. Second, interpersonal misunderstandings (addressed through repair) relate to political thinking and intergroup relations. For instance, feeling misunderstood often precedes online incivility [45], while feeling understood predicts out-group trust and forgiveness [46]. Repairs are generally absent from conventional models of online interaction quality, such as deliberative theory [47]. Integrating online repairs into models of online interaction quality may help platforms construct better environments for building mutual understanding.

### Research questions

This article explores the Other-initiations of repair on Reddit. The platform comprises various “subreddits”, each with unique rules and norms [9]. All subreddits have the same structural features, where individuals create a post (e.g., story, question, picture, video, etc.) that others discuss in the comments section. We chose Reddit because it enables the study of repair across communication contexts (subreddits) while holding the medium of communication constant. Some subreddits are expected to have different opportunities for misunderstandings, and consequently repairs, to emerge. For instance, r/changemyview involves trying to change the original poster’s perspective, possibly increasing opportunities for misunderstanding and repair, while r/aww involves posting cute pictures, possibly reducing opportunities for misunderstanding and thus repair.

Our research compared Other-initiations of repair across 25 different subreddits. Other-initiations were chosen as they have been the object of cross-cultural studies of face-to-face repairs [3, 31]. We use the term “Other-initiations” to denote all Other-initiated repairs that have been completed by the author of the trouble source (Self-completed), another participant (Other-completed), or had no completion. We compared Other-initiations across subreddits through two exploratory research questions:

RQ1: How does the distribution of Other-initiations vary across subreddits?

RQ1 explores how the frequency of Other-initiations varies across virtual public spheres. These locales differ from offline cultures in that they are structured by different modalities and norms. With regards to modalities, Dingemanse and colleagues’ [3] cross-cultural study indicates that the repair system varies across different physical environments for social interaction (e.g., if a participant is doing something whilst talking). While not fully analogous, subreddits have different modalities of interaction depending on the nature of the subreddit [9]. For instance, some are focused more on deliberation and debate (e.g., r/changemyview), others more on sharing jokes (e.g., r/funny), and others on sharing pictures (e.g., r/aww).

It is unclear how the frequency of Other-initiations varies across subreddits. On the one hand, findings from offline interactions [3] suggest different modalities should create variance between subreddits. On the other hand, these same results find little variance in initiation frequencies across different cultural contexts. Because subreddits have different norms that relate less to the modality of communication–for instance, subreddits may vary by political ideology and discussion points (e.g., r/politics, r/coronavirus, r/brexit)–they arguably have different cultures, suggesting less variance in frequency of repairs across contexts of interaction. RQ1 therefore seeks to explore how modalities and cultural norms affect repair initiations in online communications, through an examination of the distribution of repairs across subreddits.

RQ2: How does the emergence of Other-initiations vary across subreddits?

RQ2 explores the temporal features of repair initiations, exploring whether there is variation in their emergence across subreddits. In synchronous interaction, the time between repairs appears invariant across languages [3], suggesting that, as an interaction increases in length, the likelihood of a repair initiation increases at a similar rate across cultural context. The first turn at which an initiation occurs is also the first time a misunderstanding is made visible. By examining the emergence of repair initiations, RQ2 thus also examines how and when misunderstandings emerge (and are resolved) across different virtual public spheres.

Our research questions are exploratory because the study of repairs in online interactions is in its infancy, meaning confirmatory studies could accidentally obfuscate important features of the repair system that are currently unknown to researchers. This study aims to provide insight on how modality and cultural norms (represented in subreddits) change the system of repair. In face-to-face interactions, repairs vary systematically according to disruptive environmental features [3] but we do not know how disruption translates to online interactions. This study provides an exploratory overview of subreddit variations in repair initiations as a guide for future research into repairs in online forums. Exploratory studies are an important step for future confirmatory studies, helping researchers form testable hypotheses from empirical findings [48].

## Materials and method

We use exploratory statistical analysis to address our research questions. We choose statistical methods as comparative studies require quantitative methods [49, 50]. Given that the role of corrective actions in online interactions is not well-documented [23, 33], exploratory results are useful for identifying potential areas of further study. We explore RQ1 using mixed effects modelling [51] and RQ2 through survival analysis [52].

### Ethics

The data collection and analyses were approved by the London School of Economics and Political Science’s Research Ethics Committee (Ref: 56581). The requirement for informed consent was waived by the Research Ethics Committee. This is because, first, the volume of online interactions data makes it unfeasible to contact individual participants; second, Reddit data is publicly available and is therefore already openly accessible; third, Reddit has a norm of anonymity, where it is against sitewide rules to post personal identifying information. While there are exceptions to the anonymity norm (e.g., “ask me anything” threads where a celebrity is interviewed by the community), users generally withhold personal information and use non-identifiable usernames.

To mitigate the lack of informed consent, we took extra precautionary steps to anonymize the data. First, we anonymized all user information by replacing unique identifiers (e.g., usernames) using text analysis. Second, we excluded all personal and identifiable information (e.g., dates, conversation ids) from the final dataset included for analysis. We also contacted Subreddit moderators prior to data collection and provided two opportunities to opt-out the subreddit from the study (initial email and follow up). Four subreddits who declined to participate were removed and a further subreddit was removed after being banned from Reddit. Data was collected using the Reddit Application Programming Interface between the 7^th^ and 23^rd^ of February 2022 by randomly sampling post IDs and downloading all relevant replies.

The waiving of informed consent means that sharing the data openly may be against the wishes of the participants. We have therefore only made a minimal numerical dataset (S1 Data) available for replicating our findings. However, for purposes of transparency, we have provided illustrative examples of repair sequences in the coding manual (S1 File) and made available a larger sample of repair sequences for scrutiny (S3 File). Furthermore, we have included the code used for downloading the raw textual dataset from the Reddit API in the Jupyter notebook used for replicating the study’s findings (S2 File). The provision of the code is contingent upon the researchers obtaining necessary permissions and ethical clearance. The Jupyter notebook and numerical data used to produce the study’s results is also made available through a GitHub repository for ease of replication (see https://github.com/alexiamhe93/RedditRepairInitiations).

### Data

**Fig 1:**
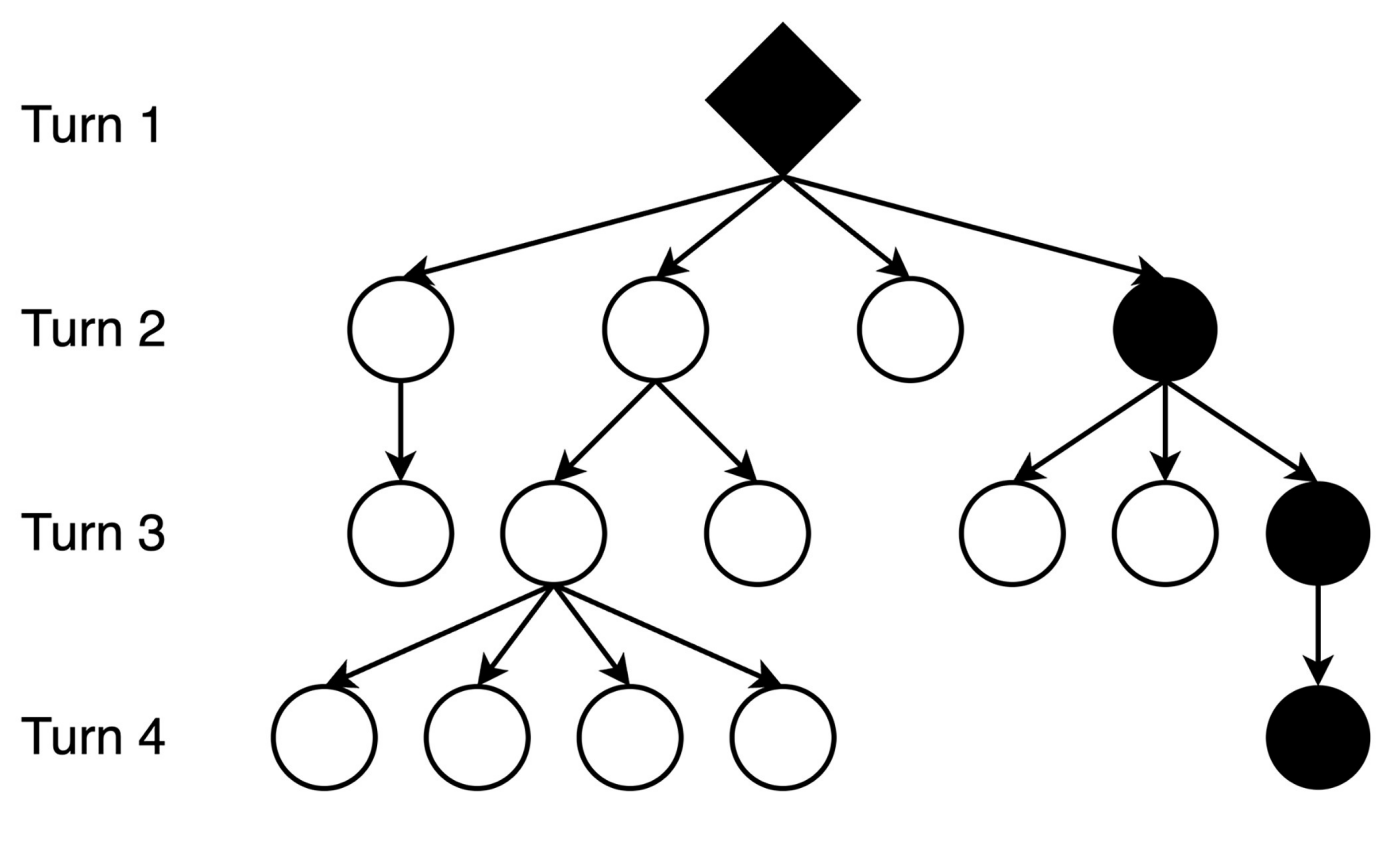
Online interaction data structure. The diamond represents the post and the circles represent comments. The filled in shapes illustrate a single comments thread, whose length is measured in turns.

Our full dataset comprised of 150 unique interactions sampled from 25 subreddits (n = 3,750). An interaction refers here to a post and all comments underneath it. Fig 1 illustrates the structure of a single Reddit interaction as a directed network graph, emerging from the post (root node) and growing out into branches based on the “reply-to” function. We refer to each of these branches as a comments “thread”, reflecting the typical terminology used by internet users. A post or comment is termed a “turn”, where the post is always the first turn in a thread or interaction. We define the total number of turns in an interaction as its “size” and define the number of turns in a thread as its “length”. In Fig 1, for instance, the interaction is 16 turns in size with 10 threads varying from two to four turns in length. The total dataset consists of 157,667 turns, belonging to 3,750 interactions and 93,441 threads.

We chose the term “interaction” to represent a post-comments set as it is the natural organization of Reddit interactions; readers click on individual posts to access and engage in the comments section. Threads were monitored as these are structurally similar to face-to-face interactions, where individuals take turns in a linear fashion [18, 50]. Reddit interactions differ from their synchronous counterparts as they have both the interaction and thread level of organization, as opposed to only the latter. This unique modality allowed us to examine how repair initiations emerged as interactions grow in size and as threads grow in length, reflecting the unique properties of online interactions.

Our study only focused on English-language subreddits, and they were sampled for variation in different interactional goals and topics (Table 2). We focused on discussion based subreddits (e.g., r/changemyview, r/unpopularopinion, r/todayilearned), political subreddits (e.g., r/politics, r/worldnews, r/brexit), question-answer based subreddits (e.g., r/iama, r/askreddit, r/explainlikeimfive), academic subreddits (e.g., r/science, r/psychology), and various media sharing subreddits (e.g., r/movies, r/music, r/publicfreakout, r/videos). The sampling sought to provide a breadth of different modalities of communication and norms to allow for comparative analysis.

**Table 2:** Subreddit sample and descriptions. We sampled 150 interaction sets from each subreddit.

Subreddit	Description
r/askreddit	Participants ask general questions for other Redditors to answer.
r/aww	Participants share media content they consider cute.
r/books	Participants discuss books.
r/brexit	Participants discuss Britain’s exit of the European Union.
r/changemyview	Participants seek to change each other’s perspectives.
r/coronavirus	Participants discuss the corona virus and its implications.
r/explainlikeimfive	Participants ask for a simple explanation to something confusing.
r/food	Participants discuss food.
r/funny	Participants post funny media content.
r/iama	Participants interview a known figure (e.g., a celebrity).
r/jokes	Participants share jokes.
r/lifeprotips	Participants share tips for helping others.
r/movies	Participants discuss movies.
r/music	Participants share music.
r/pics	Participants share photographs.
r/politics	Participants discuss various aspects of politics.
r/psychology	Participants discuss psychological research.
r/publicfreakout	Participants share media of people losing composure publicly.
r/science	Participants discuss scientific topics and research.
r/showerthoughts	Participants discuss unusual thoughts or ideas.
r/todayilearned	Participants discuss facts they have recently learnt.
r/unpopularopinion	Participants share a controversial opinion on any topic.
r/videos	Participants share video content.
r/wallstreetbets	Participants discuss financial trading.
r/worldnews	Participants discuss international news.

### Data coding

We coded this data in a manual scoring stage, where Other-initiations of repair and their completions were identified, and an algorithmic stage, where the manually coded data was used to fine-tune a BERT language model [53]. The codebook used for manual scoring is included in the supplementary materials (S1 File). The manual coding stage was done in two rounds. First, two coders were trained to identify the linguistic indicators of initiations (Krippendorff’s α = 0.80; 95% absolute agreement). Second, the data was recoded for completions, ensuring that initiations could be considered part of a repair sequence.

Initiations were operationalized in three types of clarification requests of varying degrees of specificity [3]: “open requests” specify little about the trouble source (e.g., “huh?”); “restricted requests” specify the trouble source (e.g., “What did you mean by X?”); and “restricted offers” provide a possible solution to the trouble source (e.g., “When you said X, did you mean Y?”). Completions were coded based on whether the initiation was addressed by the participant who provided the trouble source (Self), another participant (Other), or remained uncompleted. This meant we identified instances of Other-initiated Self-repairs, Other-initiated Other-repairs, and attempts at repair without completion. Table 3 provides an empirical example of a restricted offer and Table 4 an example of a restricted request, alongside their corresponding trouble source and completion. For further examples of the manual coding, we have included a random sample of the different repair types in the supplementary materials (S3 File). This includes 20 examples of restricted requests, 20 examples of restricted offers, and 8 examples of open requests (the total number identified in the data).

**Table 3:** Example of a restricted offer in an r/explainlikeimfive interaction.

Turn	Author	Text	Repair sequence
1	Self	Title: ELI5: How exactly does potential difference work? I still don’t get it. Body: Yeah the title.	Trouble source
2	Other	Gravitational? Electrical?	Restricted offer
3	Self	Electrical please	Self-completion

**Table 4:** Example of a restricted request in an r/askreddit interaction.

Turn	Author^a^	Text	Repair sequence
1	Other	What if you really wanted to post a video but for some reason it wasn’t letting you, so you created your own sub to see if it was specific to that certain sub from before and you still couldn’t?	
2	Self	Then I can’t post a video.	Trouble source
3	Other	But why can’t you?	Restricted request
4	Self	The question, which you proposed doesn’t let me.	Self-completion

^a^Authors are organized by who initiated the trouble source [1] and therefore starts with the Other.

The example of a restricted offer in Table 3 is extracted from the r/explainlikeimfive subreddit (ELI5). The first turn involves a question about how “potential difference” operates. The Other then initiates a repair by asking what type of potential difference the Self is talking about (“Gravitational? Electrical?”). This provides two options for the Self to consider, meaning the initiation is coded as a restricted offer. The Self then clarifies they are talking about electrical potential difference, thereby completing the repair sequence.

The example of a restricted request in Table 4 is extracted from the r/askreddit subreddit. The repair sequence follows a question from the Other in the first turn about why they cannot post a video on the subreddit. The trouble source involves a simple answer to the question that the Other finds dissatisfactory. They therefore initiate a repair in the second turn, asking specifically why the Self is unable to post a video. This initiation is a restricted request as it does not provide a possible solution but still points to the source of the trouble. In the third turn, the Self clarifies that the Other’s original question limited their response.

Following manual coding of the data, a secondary stage was used to code the full dataset for repair initiations using machine learning. Specifically, a Bidirectional Encoder Representations from Transformers (BERT) [53] model was fine-tuned by using 70% of the manually coded data and validated using the remaining 30%. The model was trained to perform binary predictions of initiations and had moderately high accuracy (AUC PR = 0.81, macro average F1 = 0.88, accuracy = 0.94). After validation, the BERT classifier was used to code the remaining turns in the dataset for whether they contained an initiation. Instructions for downloading the trained model and details of its validation (including analysis of misclassifications) can be found in the supplementary materials (S2 File).

### Analysis 1: Mixed effects model

We use descriptive statistics and a generalized mixed effects model [54] to address RQ1 and examine how the frequency of initiations varies across subreddits. Mixed effects models are an extension of linear regression that allow for the inclusion of random effects in the model. Conventional regression only uses fixed effects, which are explanatory variables assumed to apply in the same way across all data points. Conventional regressions only have one intercept, which in this case would be the mean level of repair initiations when holding all fixed effect explanatory variables constant. Random effects, in contrast, are categorical explanatory variables that allow for different intercepts for each category. In this case, a subreddit is assumed to be a random effect, where the mean level of repair initiations varies for each community. Random effects are useful when there is a hierarchical interdependence between data points. In this case, turns are embedded in interactions which are themselves embedded in subreddits.

In our model, the response variable is whether a turn contains a repair initiation (CR). The fixed effects include the turn number (t; post = turn 1), the standardized word count (wc) of the turn, and standardized count of the previous turn (prev_wc). These fixed effects are chosen as covariates for examining the differences between subreddits as they are natural features of the Reddit interactions. The scaling was performed to reduce potential problems with model assumptions due to heavily skewed distributions. The two fixed effects represent a unique identifier for each interaction (conv) and the subreddit identifier (sub). The final model is as follows and was selected following a backwards stepwise process (see S2 File for further details and code for replication):

CR∼1+t+wc+prev_wc+wc:prev_wc+(1|conv)+(1+wc|sub).

### Analysis 2: Survival analysis

We address RQ2 using survival analysis, a set of statistical techniques developed by actuaries and medical researchers for estimating how different variables affect the time until a person dies [55]. Survival analysis thus concerns how the probability of an event occurring changes over time. Our study uses survival analysis to focus on the duration before a conversation or thread undergoes a repair initiation. Survival analysis is designed for handling "censored" data, meaning instances where the event of interest, like repair initiation, does not occur. This method allows us to include all threads and interactions in our analysis, even those that never experience a repair initiation. By incorporating these non-event cases, we can gain insights into both the occurrences and absences of repair initiations, offering a more complete understanding of communication dynamics.

Survival analysis requires estimating the hazard function, which quantifies the instantaneous likelihood of a repair initiation occurring at a specific time, given it has not yet occurred. This estimation requires a vector of durations *T* and a vector of whether the event *E* occurred. Durations are either the time to the event or the time of last observation (censored) if the event did not occur. For our thread data, *T* represents the number of turns until an initiation or the total turns a thread lasted. In our interaction data, *T* is either the number of comments before an initiation or the total count of comments in the interaction. We employ both non-parametric and parametric methods to estimate the hazard function and calculate the cumulative distribution of an initiation occurring (see S2 File for further details and code for replication). This allows us to compare the time-to-initiation across different subreddits.

Our survival analysis uses various (univariate) parametric and non-parametric estimators. For the former, we used the Kaplan-Meier [56] estimator to calculate the hazard rate and the Nelson-Aalen estimator [57, 58] to calculate the cumulative hazards. For the latter, we explored common univariate models through observation of the produced residuals and partial AIC.

## Results

### Analysis 1: Distribution of repairs across subreddits

Repair initiations were common across the full dataset. Of the 3,750 interactions, 2,193 (58.48%) contained at least one repair initiation. These interactions contained 92,441 individual threads, of which 34,642 (37.47%) contained at least one initiation. The threads and interactions are comprised of 157,667 unique turns, of which 26,249 (16.65%) were identified as repair initiations. The different subreddits were found to have different rates of repair initiations, both in the average number of turns classified as initiations (M = 15.56%; SD = 9.75%) and in the number of interactions containing a repair (M = 48.88%; SD = 29.08%).

**Fig 2:**
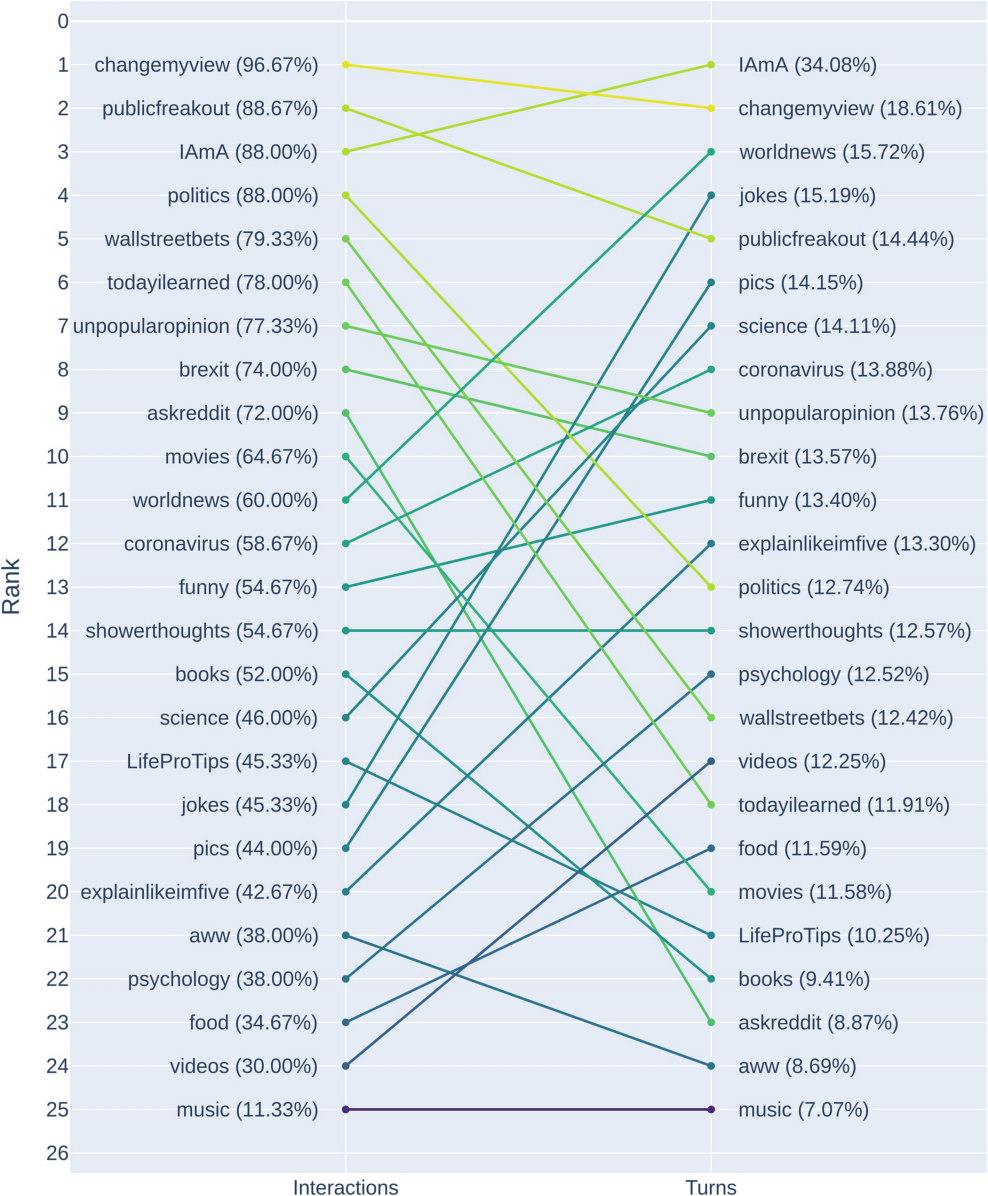
Subreddit ranks based on the percentage of repair initiations at the turn and interaction level. The turns refer to all 150 posts and their comments for a subreddit, the interaction level refers to all the turns relating to a post.

Fig 2 illustrates the ranking of subreddits based on the rate of initiations at the turn and interaction levels. The figure illustrates how the rate of repair initiations at the interaction level varies more (min = 11.33%; max = 96.67%) than at the turn level (min = 7.07%; max = 34.08%). It also demonstrates how the subreddits change rank depending on the level of analysis. For instance, r/changemyview has the highest proportion of initiations at the interaction level, where 145 (96.67%) interactions contain an initiation. However, at the turn level, r/iama has the highest proportion of repair initiations, with 34.08% of turns containing initiations. The r/music subreddit and r/showerthoughts subreddits are the only ones to hold the same rank across the levels, the former having the lowest rate of repair initiations.

Drilling down into the characteristics of repair sequences, the manual coding identified 335 Other-initiations of repair across 585 interactions. We find that initiations were most frequently completed by the Self (n = 187) followed by a second Other (n = 102). The remaining initiations (n = 66) were not completed by either Self or Other. The differences between completion speaker were statistically significant (Chi-square = 7314.14; p< = 0.001), providing supporting evidence for a preference for Self-completion. We also find that more specific repair initiations are used more frequently, where restricted offers (n = 189) are more common than restricted requests (n = 158), and both are considerably more common than open requests (n = 8). The differences between the frequency of initiation types were statistically significant (Chi-square = 148.37; p< = 0.001), providing supporting evidence for a preference for more specific repairs.

Given that the BERT classifier was not trained to identify completions, we could only estimate the completion of initiations in the full dataset by examining who responded. We counted each occurrence of Self and Other replies to the initiation to provide this estimate. We found that 7,308 (27.85%) initiations were replied to by Self exclusively, 5,432 (20.7%) by one or more Others, and 1,746 (6.65%) by both Self and Other(s). While these statistics are only tentative, they support the manually coded data in demonstrating a preference for Self-completion for cases where initiations were completed. Significantly, we found that many initiations (n = 11,756; 44.80%) had no replies, indicating a lack of completion. This rate of uncompleted repairs is a robust conservative estimate of the true rate, as no reply necessarily indicates a lack of explicit completion. These uncompleted repairs happen early on in a thread (M = 3.30; SD = 2.02), with 55.06% of them occurring in the second turn.

The results of the mixed effects model (Table 5) provide more details on the potential predictors of initiations and the different rates of initiations at the turn level. The goal of the model was to predict whether a turn contained a repair initiation, whilst considering the hierarchical relationships between subreddits and interactions. All fixed effects were found to be significant at the 0.1% level of statistical significance. The model finds that, as interactions progress, each subsequent turn slightly decreases the likelihood of a turn being a repair initiation by 9% (OR = 0.91; CI = 0.90–0.92). This suggests initiations are marginally more likely at the start of an interaction. The odds ratio for the word count (OR = 0.78; CI = 0.63–0.79) indicates that longer turns are associated with a 29% decrease in the likelihood of an initiation. This suggests initiations are generally shorter than other turns. Conversely, the odds ratio for the previous word count is 1.48 (CI = 1.46–1.51), indicating that longer previous turns increase the likelihood of a repair initiation by 48%. This implies that a long turn increases the likelihood of the subsequent turn containing a repair initiation. The interaction term between current and previous word counts (OR = 0.95, CI = 0.93–0.96), indicates that the combined effect of current and previous word counts slightly reduces the likelihood of a repair. This suggests that longer turns in the interaction are less likely to be a repair initiation when the previous turn is also long.

**Table 5:** Mixed effects model results.

Predictors	Odds Ratios	CI 95%	p-value
Fixed effects			
(Intercept)	0.21	0.17–0.24	< 0.001
t	0.91	0.90–0.92	< 0.001
wc	0.71	0.63–0.79	< 0.001
prev wc	1.48	1.46–1.51	< 0.001
wc × prev wc	0.95	0.93–0.96	< 0.001
Random effects			
Group	Name	τ	
conv	(Intercept)	0.23	
sub	(Intercept)	0.17	
	wc	0.07	
σ^2^	3.29		
ICC	0.12		
Observations	153,916		
Marginal R^2^ / Conditional R^2^	0.08 / 0.19		

The results show that approximately 12% of the total variance in repair initiation (ICC = 0.12) is attributed to between-group variance, highlighting that differences between individual interactions and subreddits significantly influence repair dynamics. The variability of repair initiation is larger across interactions (τ00 = 0.23, SD = 0.48) than across subreddits (τ00 sub = 0.17, SD = 0.41). This suggests that the rate of initiation varies less between subreddits than it does across specific interactions. A moderate positive correlation between subreddit intercepts and log word count random slopes (ρ01 = 0.22) suggests that subreddits with higher baseline repair rates tend to exhibit a more pronounced effect of word count on repair initiation.

The model explains approximately 19% of the variance in repair initiation when considering both random and fixed effects (Conditional R^2^ = 0.19), with 7.7% explained by fixed effects alone (Marginal R^2^ = 0.08). This indicates that while the specific predictors—turn number, word counts, and their interaction—have an impact on whether a turn is an initiation, much of the variability is due to differences in interactions and subreddits.

### Analysis 2: Emergence of repairs across subreddits

**Fig 3:**
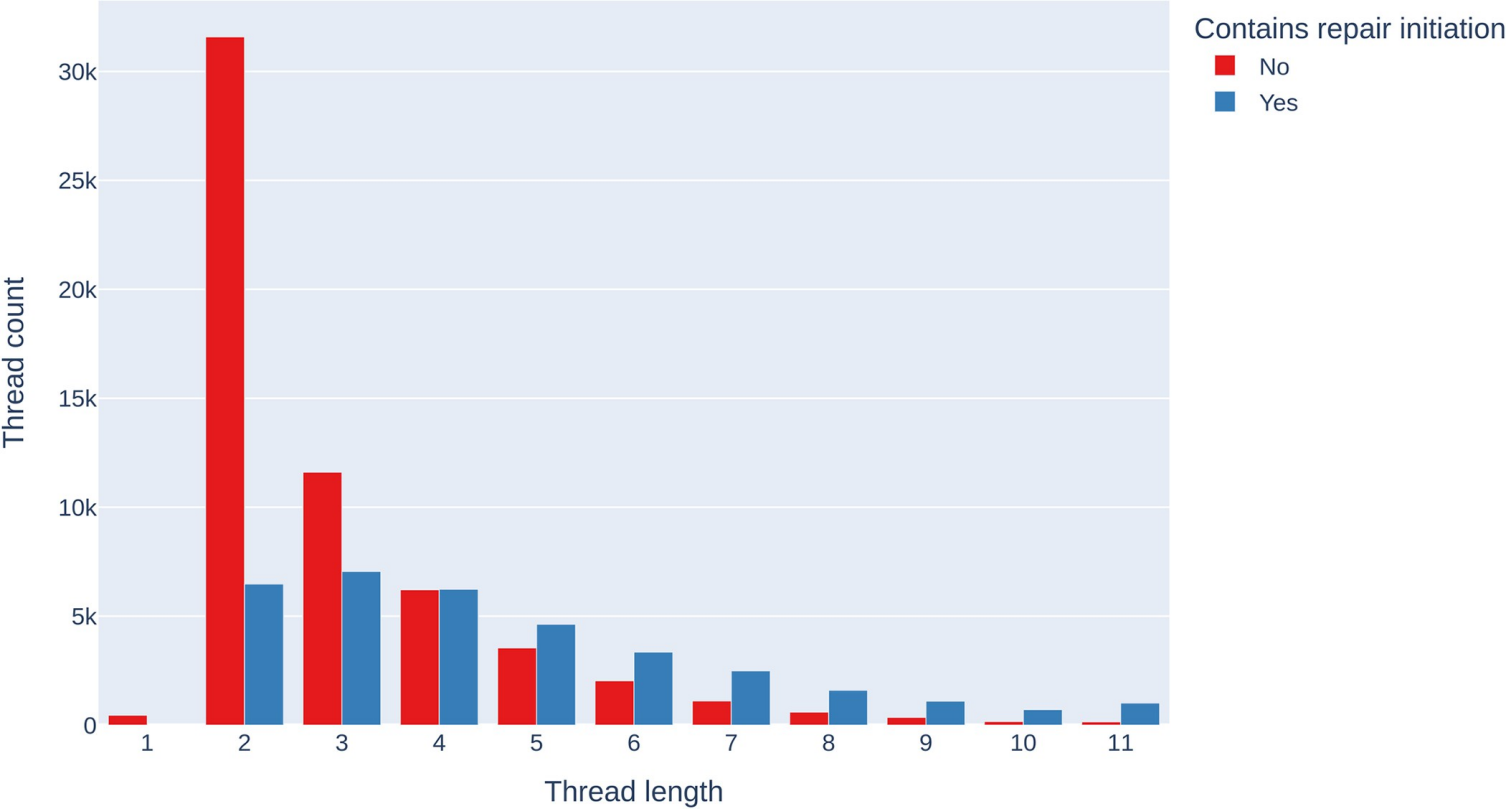
Thread count against thread length. A thread’s length refers to the number of comments organized underneath the post through the reply-to function.

Fig 3 shows the number of threads that do and do not contain at least one repair initiation, and their length. It shows that thread lengths are highly skewed (Median = 3; M = 3.60; SD = 2.03), with 57,170 (61.84%) having a length of three turns or fewer. We also observe that the proportion of threads containing an initiation increases with the length of the thread. For the first four turns, there are fewer interactions with an initiation than without. For all subsequent turns, there are more threads that contain an initiation than threads that do not.

**Fig 4:**
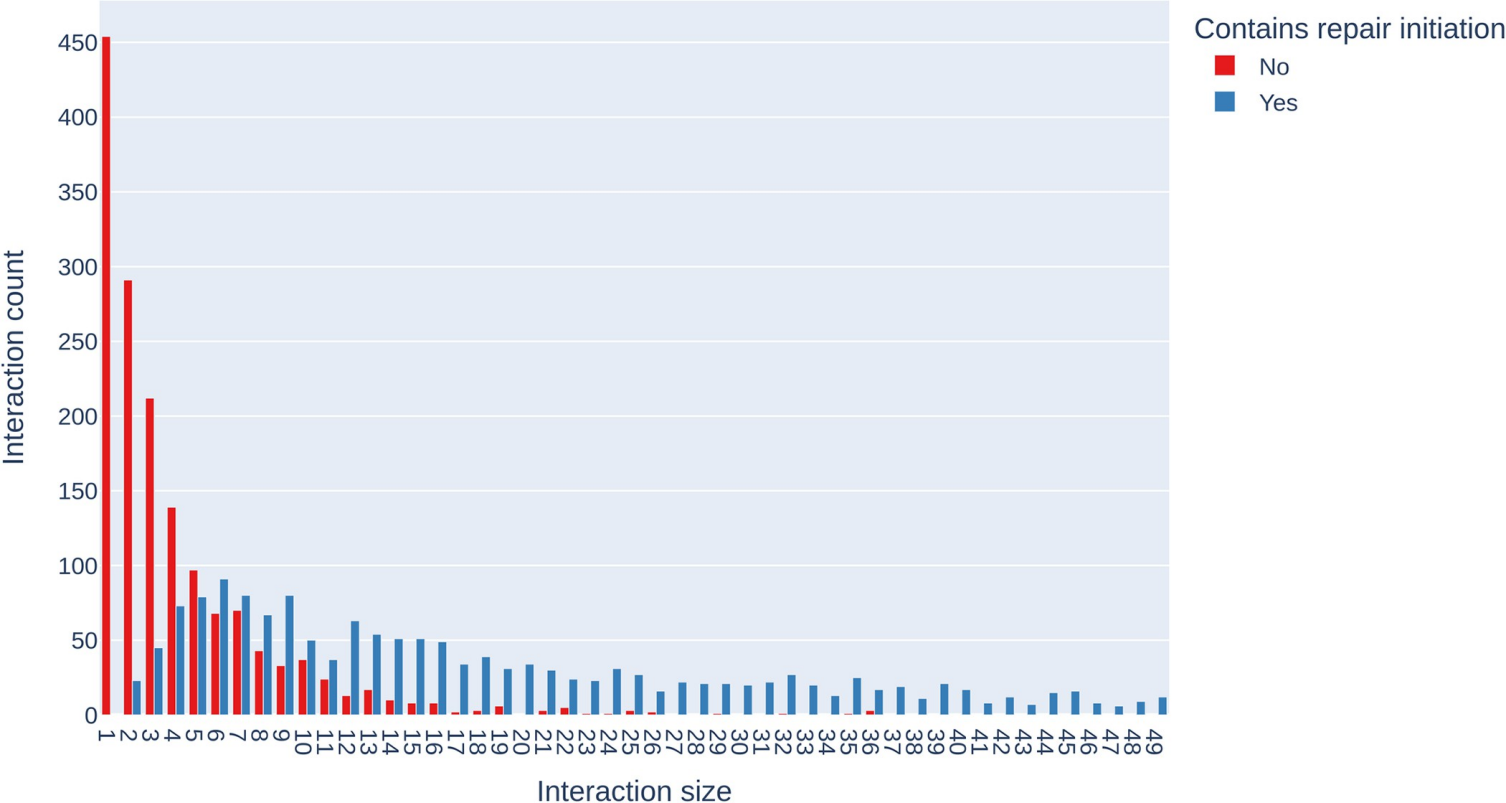
Interaction count against interaction size. An interaction’s size refers to the total number of turns in the whole post-comments set. Due to the graph’s long tail, it has been truncated at interactions smaller than 50 turns in size.

When examining interactions, we find a similar pattern, where the number of interactions containing a repair initiation increases as a post gathers more comments underneath it. Fig 4 shows the number of interactions that do and do not contain at least one repair initiation and their size. As with the threads, the size of interactions on Reddit is highly skewed (Median = 9; M = 42.04; SD = 94.53), with far more small interactions than long. Unlike threads, which do not exceed 11 turns, the number of turns in an interaction has a very long tail, with the largest interaction containing 501 unique turns. The number of interactions without an initiation decreases considerably as the interactions increase in size.

Table 6 shows the median length of threads and median interaction size for each subreddit. We observe significantly more variations in the median interaction size (M = 15.28; SD = 18.43) than between median thread lengths (M = 2.76, SD = 0.72). This suggests that thread lengths are similar across subreddits but that the amount of engagement with posts varies considerably. For instance, r/iama has a median size of 87.5 turns but median thread length of 4 turns, the same as r/changemyview (Median size = 42). The r/music subreddit is unusual in that the median thread length is longer than the median interaction size, which is only a single post with no comments.

**Table 6:** Median interaction size and thread length. Interaction size refers to the total number of turns in a post-comments set and thread length refers to the total number of turns in a single set of comments organized by the reply-to function.

Subreddit	Median Interaction Size	Median Thread Length
r/iama	87.5	4.0
r/changemyview	42.0	4.0
r/politics	34.0	3.0
r/publicfreakout	29.0	3.0
r/wallstreetbets	23.0	2.0
r/brexit	18.0	3.0
r/movies	17.5	3.0
r/todayilearned	17.0	3.0
r/askreddit	17.0	2.0
r/unpopularopinion	17.0	2.0
r/books	11.0	2.0
r/coronavirus	9.5	4.0
r/worldnews	8.0	4.0
r/funny	7.0	2.0
r/lifeprotips	7.0	3.0
r/showerthoughts	7.0	2.0
r/explainlikeimfive	6.0	3.0
r/jokes	4.0	3.0
r/pics	4.0	3.0
r/science	4.0	3.0
r/aww	3.5	2.0
r/food	3.0	2.0
r/psychology	3.0	2.0
r/videos	2.0	3.0
r/music	1.0	2.0

**Fig 5:**
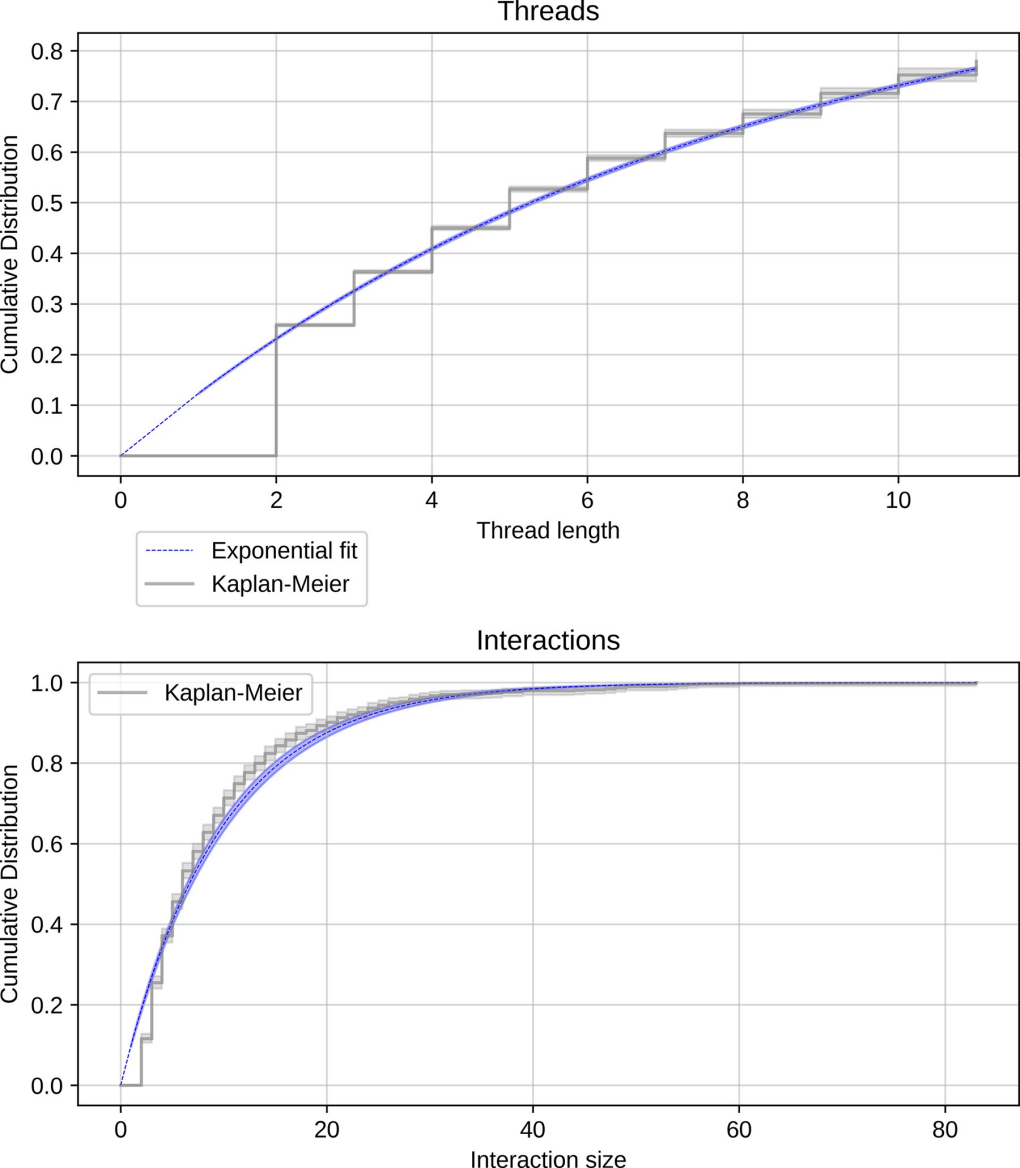
Cumulative distribution of repair initiations at the thread and interaction levels. The thread level is a single reply-to chain of comments and the interaction level all comments and the post, inclusive of all threads. The cumulative distribution was calculated using a Kaplan-Meier estimator (grey lines) and an exponential fitter (blue lines), subtracting the resulting survival function from one.

To directly estimate the emergence of repair initiations, Fig 5 illustrates the cumulative distribution of a repair initiation calculated using the Kaplan-Meier estimator at both the thread and interaction level. We observe that the likelihood of a thread or interaction experiencing an initiation increases for each additional comment. For the threads, we find that the median survival time is 5 turns, meaning a repair initiation becomes more likely than not at the 6^th^ turn. The results are similar for the interactions, where the median survival time is 6 comments, indicating that an interaction is more likely than not to contain an initiation by the 7^th^ turn. The cumulative distribution for the interaction size approaches 1, where 99% of interactions are expected to contain an initiation after reaching 37 comments in size.

**Fig 6:**
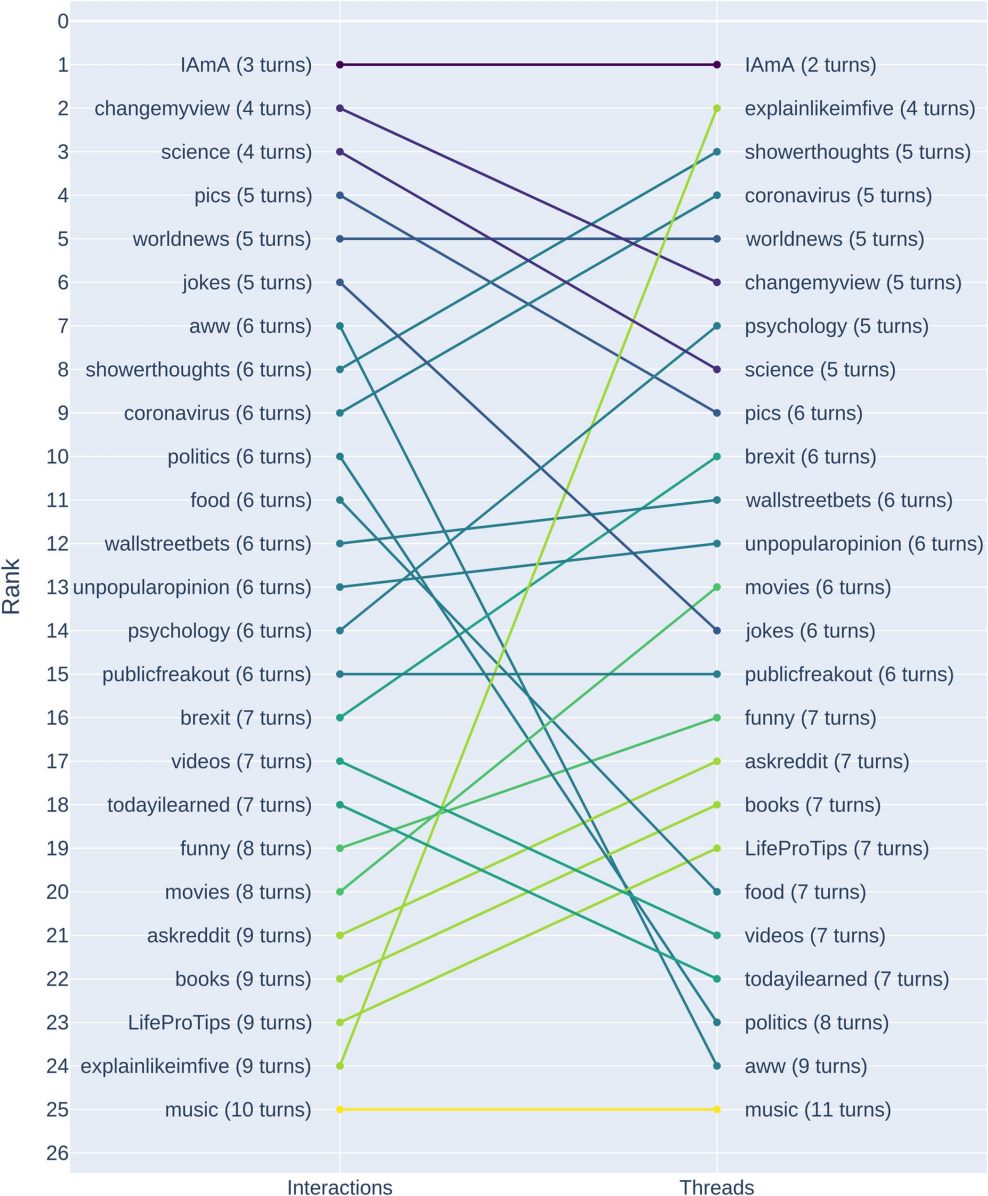
Median survival times for subreddits at the thread and interaction level. The median survival times were estimated using a Kaplan-Meier estimator.

**Fig 7:**
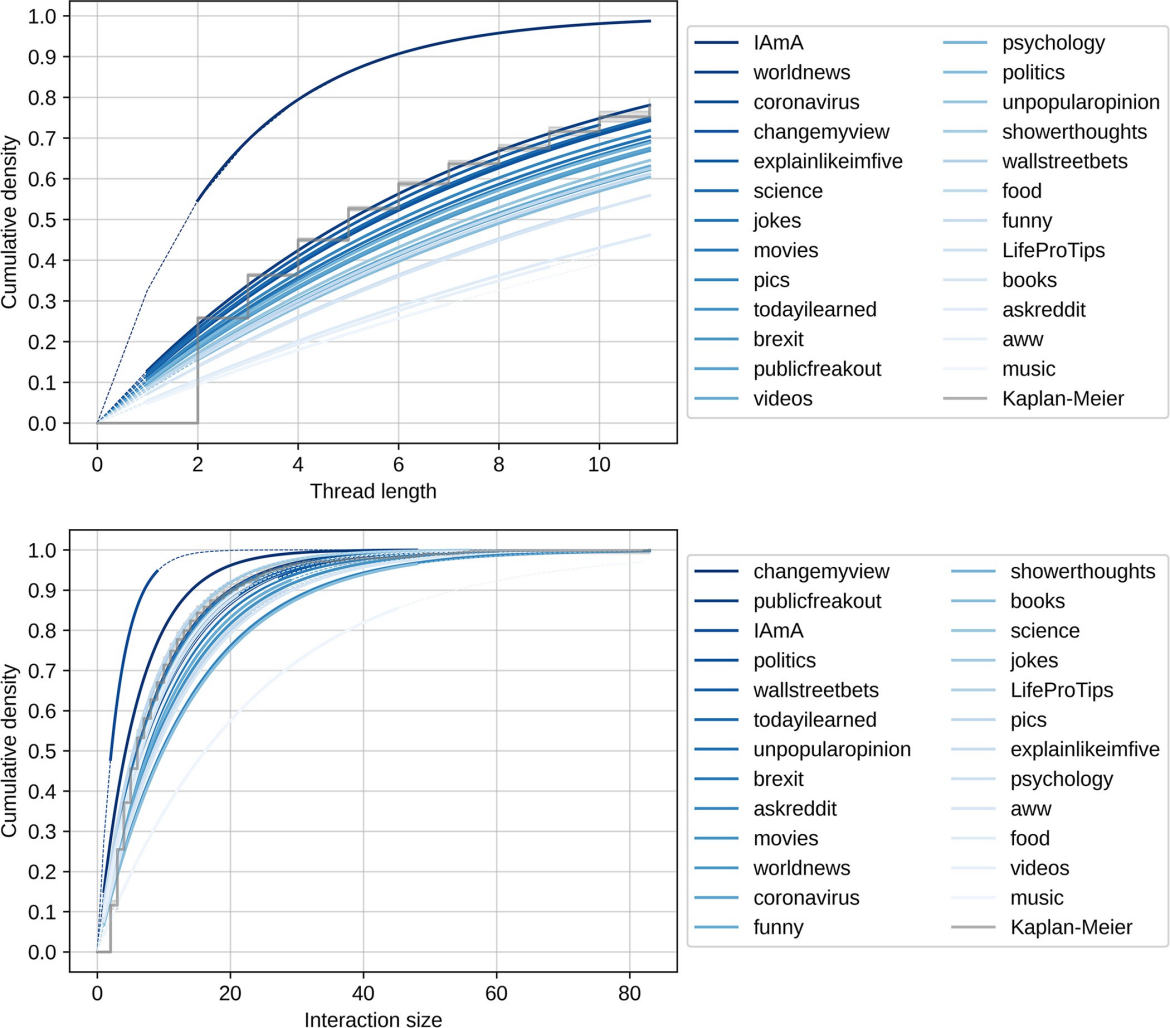
Cumulative distribution of repair initiations at the thread and interaction levels across subreddits. The thread level is a single reply-to chain of comments and the interaction level all comments and the post, inclusive of all threads. The cumulative distribution was calculated using a Kaplan-Meier estimator (grey lines) and an exponential fitter (blue lines), subtracting the resulting survival function from one. Only the exponential estimators are shown for the subreddits for clarity.

To examine the differences in the emergence of initiations across subreddits, we estimated the median survival time for each subreddit using the Kaplan-Meier estimator for both threads and interactions. Fig 6 shows the median survival times for each subreddit across interactions and threads, illustrating the differences in their rank. We can observe that r/iama has the lowest medium survival time for both interactions (S(t) = 3) and threads (S(t) = 2), with initiations happening soon after a post has been created. The r/music subreddit has the highest median survival times, with threads never reaching a point where an initiation becomes likely (there are no threads longer than 11 turns). Fig 7 illustrates the cumulative distributions across subreddits, highlighting the increasing likelihood of a repair initiation over time. We see that the likelihood increases for all subreddits over time.

## Discussion

Our results provide insights into how the repair system operates in online interactions [2, 15–17]. Examining the distribution of initiations (RQ1), we find that repair initiations are both widespread, appearing in every subreddit, and frequent, appearing in the majority (58.48%) of interactions (i.e., posts and their comments) under study. These results resemble findings from offline synchronous interactions, where initiations are found across cultures and languages [3]. Examining the emergence of repair initiations (RQ2), we find that the likelihood of a repair initiation increases over time. As interactions grow in size, they become more likely to include an initiation than not at the 7^th^ comment. Similarly, as threads increase in length, they become more likely to contain an initiation at the 6^th^ turn. These findings are similar to those of repairs in offline interaction, where the likelihood of an initiation increases over time [3].

We found that subreddits vary in both the frequency of initiations and their emergence. These variations appear related to the modality of a subreddit, specifically the underlying goals of the interaction that may influence the opportunities for trouble. We found that the r/iama subreddit was a clear outlier in having significantly more initiations than other subreddits at the thread level. The initiations also emerged sooner than in other subreddits. This can be explained by r/iama’s unique modality, where, rather than interacting with each other, Redditors interview and interact with a person of significance, such as a celebrity or representative of an organisation. It follows that initiations would be more frequent as the aim of the subreddit is to seek clarification. Similarly, the r/music subreddit consistently had the lowest frequency of initiations. This may be explained by the subreddit’s goal of sharing r/music and its low interactivity; it was the only subreddit to have more posts without comments than posts with one or more comments.

Interestingly, we found that subreddits changed rank in the frequency and emergence of repair initiations depending on the resolution of the analysis (interactions or turns). As an example of frequency ranks, r/iama may have the largest number of repair initiations, but r/changemyview has more interactions with initiations. This might be explained by some r/iama interviews not receiving much attention (generating fewer interactions with initiations) and r/changemyview’s goal of altering perspectives generating more misunderstandings. As an example of emergence ranks, interactions in r/explainlikeimfive are the second slowest in experiencing an initiation (9 turns), but the second fastest (4 turns) in comments threads. The r/explainlikeimfive subreddit involves the post asking for clarification on some topic, with the comments providing explanation. This may explain why it takes longer for an initiation to emerge as the second turns are answering the question posed in the post. On the other hand, the threads may experience a repair sooner than other subreddits as the participant who posed a question in the post seeks clarification on the others’ answer to their question.

As Reddit interactions progress through time, they grow in both size (interaction level) and length (thread level) to form a reply tree (Fig 1). This makes them structurally different to offline synchronous interactions, whose size and length are equivalent due to their linear progression. Our findings highlight that modality changes the frequency and emergence of initiations in a similar way to offline interactions [3]. However, they also highlight how subreddit’s initiation rates vary depending on the thread length and interaction size. This is significant because it highlights how the structural design of social media platforms can influence the way misunderstandings emerge and are resolved. Our results therefore reveal both the complex interaction between repair initiations and a subreddit’s context (norms and modality), and their interaction with the unique structural features of online interactions.

We also found supporting evidence of a preference for Self-completion [25–30], and for a preference for more specific types of initiation [3]. On the topic of completions, we found that multiple people may reply to the same initiation either in place, or in combination with, the Self. This is a peculiar feature of the asynchronous modality of Reddit, where each comment can spawn a new thread for every reply and thus branch out into new interactions. In offline interactions, however, the synchronicity of the medium bounds completions to the immediate moment, making it difficult for participants to refer back to the trouble source after only a few turns [1, 20].

Importantly, we also found that nearly half of repair initiations went without reply and, therefore, without a completion. Our results therefore suggest that the distribution of repairs online and offline are initiated in similar ways but differ in how they are completed. The completion rates may be explained by how the average Reddit thread is only three turns in length, with longer threads becoming increasingly less frequent. Any repair initiation posed in the last comment of a thread will go without a reply. Shorter thread lengths mean fewer replies and, therefore, fewer completions. This explanation is supported by the finding that most repair initiations without reply were in the second turn.

The consequences of low completion rates are currently unknown. For instance, low rates of completion could be problematic as they might indicate that many repair initiators never have their misunderstanding clarified, creating potential resentment towards the addressee on the part of the initiator. However, this view may be unduly pessimistic as the negative effects of uncompleted repairs may be mitigated by other contextual features. We observe that Others frequently complete the repair in place of the Self, thereby rectifying the problem of a hanging initiation. Additionally, the asynchronous nature of text-based interactions makes delayed responses more socially acceptable than in other mediums [59], thereby reducing the expectations of participants to have their questions answered. Finally, interactions may evoke several highly similar initiations, only one of which is completed. The initiator might therefore observe their misunderstanding clarified elsewhere in the interaction, but not directly in the thread where the initiation was posed. For instance, the initiator may notice that the Self has already responded to a similar initiation from a different Other.

One area where uncompleted repairs may be a significant problem is in their potential role in maintaining social norms in virtual public spheres. Dingemanse and Enfield [17] suggest that repairs are used to enforce social norms by holding other participants accountable to their perspective. In online interactions, for instance, repairs have been found to be used in activism against norm-violating oppressors [39] and trolls have been found to derail interactions by rejecting attempted repairs [6]. Because online interactions allow individuals to walk away at any point, people can choose to ignore repair initiations relating to a norm violation (e.g., “Did you mean to say that? It was quite hurtful”). Ignoring these requests has no repercussions for the norm violating Self. In contrast, the Other must either leave a misunderstanding unresolved or assume the intended meaning of the Self without further information. In other words, the lack of reply may not be deliberate (i.e., an unintentional norm violation), yet the Other may infer deliberate violation due to the lack of response.

## Limitations and future directions

There are several limitations and avenues for future research stemming from our study. First, our study does not formally examine each subreddit’s norms (e.g., ideological positions, moderation practices) and interactional goals (e.g., discussion, content reaction, interviews). Future studies should establish how these two categories interact with the distribution and emergence of repair. Based on findings from face-to-face interaction [3] and those presented in this study, we would expect less variability between subreddits with similar interactional goals, but different cultural norms, than the opposite pattern. Future studies should include subreddits in other languages than English, as to establish how language context interacts with the different norms and modalities of subreddits, and also directly compare text-based and face-to-face interaction (e.g., in a controlled experiment).

Second, future studies should focus on how cultural norms and modalities interact with the rates of repair on Reddit. Our study suggests that modality may play an important role in why subreddits vary in their rate of repair (e.g., r/iama’s high rate and r/music’s low rate). However, our study does not formally quantify the subreddit norms (e.g., ideological positions, moderation practices) and modalities (e.g., discussion, content reaction, interviews) that may be driving underlying differences in subreddits. This means that the cause of why subreddits vary in their repair rate is currently unclear. Future research on the origins of frequency variations is important for understanding whether different virtual public spheres deal with misunderstandings differently due to context-specific rules and norms.

Third, the study of how and when repair initiations are completed online requires attention. The asynchronous medium and scale of Reddit interactions means participants can choose when to reply and to whom without being held accountable for leaving a repair uncompleted. However, it is unclear whether the high number of uncompleted repairs has negative consequences (e.g., increased felt misunderstanding) or whether participants negotiate this low completion rate in other ways (e.g., seeking clarification elsewhere). The nature of completions online appears different to face-to-face interaction as Others can complete an initiation when the Self has yet to reply. Future descriptive studies that focus on how participants negotiate completions may therefore be able to shed light on the way the textual medium interacts with the repair system.

## Conclusion

Our findings suggest that, while online and offline Other-initiated repairs share similar properties, they also differ in systematic ways relating to the nature of the online medium. Similar to observations from synchronous interactions [3], we find that Other-initiations of repair on Reddit are widespread on Reddit and become increasingly likely as time progresses. However, we find that many repair initiations go without a reply and, therefore, are uncompleted. The high number of uncompleted initiations is significantly different from offline interactions, where all repair initiations expect (and usually receive) a completion at the next available opportunity [1]. The effects of these uncompleted repairs are currently unknown. On the one hand, interlocuters may have their misunderstandings answered elsewhere. On the other, interlocuters may feel unsatisfied, confused, or insulted by their initiation being ignored, causing misunderstanding and exacerbating interpersonal conflicts. This may mean that online interactions limit the potential for building mutual understanding through repairs, leaving unresolved issues to reverberate through the public sphere.

## Supporting information

S1 File Codebook for scoring repair initiations in Reddit interactions. (PDF)

S2 File Python and R code and annotations for replicating results. (PDF)

S3 File Random sample of example repair sequences. (XLSX)

S1 Data Data for replicating the results. (ZIP)
