# Supplementary material for: Conversational repairs on Reddit: Widely initiated but often uncompleted
Source: PLoS One. 2025 Jan 2;20(1):e0316618. doi: 10.1371/journal.pone.0316618 (PMC11694982; doi:10.1371/journal.pone.0316618)
Supplement: S1 File — (PDF) [file pone.0316618.s001.pdf]

S1: Codebook for scoring Other-initiated repairs in Reddit interactions

1 Introduction

This codebook details the process used for scoring Other-initiated repairs in Reddit interactions in the article titled: *Conversational repairs on Reddit: Widely initiated but often uncompleted*.

A conversational repair has three components. First, a trouble source involves a problem of miscommunication and misunderstanding that arises in the interaction [1]. Second, an initiation involves a participant alerting other speakers to the trouble source. Third, a completion involves a participant addressing the misunderstanding or miscommunication. The initiation and completion can be within the same turn (produced by one speaker) or distributed across two turns (produced by two speakers). A repair can be initiated by either the person whose statement caused trouble in the interaction (Self) or another person who noticed the trouble (Other).

In the present coding scheme, we consider instances of repair where the initiation and completions are in separate turns and independent from the trouble source. This relates to two types of repair: Other-initiated Self-completed repairs and Other-initiated Other-completed repair, where the latter is completed by a second Other (i.e., a third party). The manual coding scheme adapts the Dingemanse and colleagues' [2] typology, aimed at synchronous communication, for online text-based communications. The codebook details how different types of initiations (Section 3.2) and completions (Section 3.3) were identified and scored.

The coding of repairs were guided by three principles:

1. Next turn proof [3]. A turn was considered a repair initiation if another speaker treats it as such. This enables ambiguous or unintended cases of repair initiations (e.g., rhetorical questions, a joke) to become initiations based on the completer's interpretation.

2. Unambiguous repair initiations. A turn was considered a repair initiation if it unambiguously addresses a problem of misunderstanding (e.g., "What did you mean by that?"). These instances violate the next turn proof principle but enable the tracking of uncompleted repairs (see Section 3.2.4).

3. Conservatism in coding [2]. Some turns *resemble* repair initiations but are not actually performing that function, such as an exclamation (e.g., "WHAT?"). As such, we took a conservative approach to scoring repairs, defaulting to *no repair* if there were hesitations regarding the certainty of the repair.

2 Data structure

This codebook was designed for scoring text-based interactions conducted on Reddit. Reddit is a social media platform comprised of smaller "subreddits" with their own sets of rules, norms, and topics of discussion [4]. Subreddits are structured around posts and comments. Posts can involve sharing external media content (e.g., YouTube videos, memes, images, etc.) or pure text. Commenters then "reply" to the post (and subsequently to each other) in a reply tree. A reply tree is so-called as posts and comments can have multiple direct replies that branch off into different conversation threads.

Figure 1 shows a graphical representation of a Reddit reply tree. There are eight conversation threads contained with the reply tree, each four turns in length. Every thread begins at the post and ends at the final reply of its branch. In the highlighted thread, the first turn is the post, the second comment is Reply 1, the third is Reply 4, and the fourth is Reply 9. In this codebook, we code data as threads because it involves a linear turn-taking process between participants. While the wider reply tree is the self-contained interaction space, we chose to score threads as they resemble face-to-face interactions in their linear structure, making them more straightforward to code than the full interaction.

Figure 1: Reply tree conversation structure on Reddit

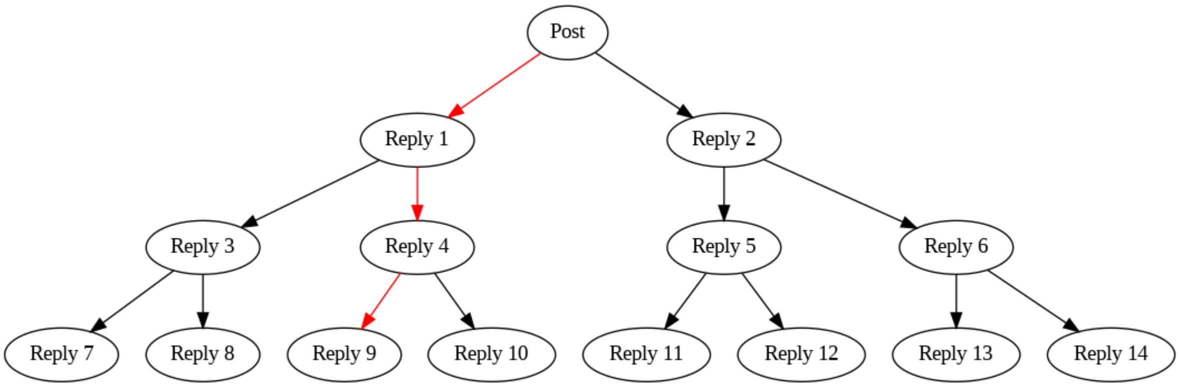

3 Coding scheme

3.1 Iterative coding procedure

Due to the contextual nature of repairs, the coding was done iteratively, adapting the codebook where necessary. The iterative process of coding began by scoring 500 Reddit threads using linguistic features. In total, we coded 500 threads, of which 250 were shared for estimating inter-rater reliability (Krippendorff's alpha = 0.80). The coding scheme was then adjusted systematically based on inductive coding of the interactions. This involved removing false positives based on the four principles and rectifying any false negatives. In total, 4,803

threads were manually coded for repair initiations, sampled from 500 Reddit post-comments sets.

3.2 Repair initiations

There are three categories of repair initiations coded for in the data, varying in their degree of specificity [2,5]. An initiation is more specific when it addresses an unambiguous aspect of the trouble source. From least to most specific, the three types of repair initiations are: *Open Requests*, *Restricted Requests*, and *Restricted Offers*. We describe the coding process below, providing empirical examples and criteria used for their inclusion and exclusion. In cases where multiple requests and offers were present, we coded for the most specific repair type.

3.2.1 Open requests

Open requests are the least specific form of repair. These signal trouble in communication without specifying what or where the issue is. Examples include interjections like "Huh?", question words like "What?", formulaic expressions like "Sorry?", or simply a question mark with no other words attached “?”. We coded for open requests in cases where the apparent intent of the statement was confusion. This includes any singular statement of confusion (e.g., “huh?”), statements of misunderstanding without specificity (e.g., “I don’t get it”), and other instances of unspecific questions where the next speaker treated it as an initiation.

Example 1 provides an example of an Other-initiated Other-completed repair using an Open request. The trouble source involves the Self commenting on a title of post in a previous turn. The first Other initiates the repair with a single question mark (open request) to

indicate that they do not understand the comment. A second Other completes the repair by stating that the first word in the title sounds like a "slang term" for female genitalia, thereby explaining why the title was "unfortunate" and completing the repair.

Example 1. Open request

Turn	Speaker	Empirical example	Repair components
1st	Self	what an unfortunate way to start the title	Trouble
2nd	Other	?	Initiation
3rd	Other (2)	The first word sounds like a slang term for vagina.	Completion

Questions and statements resembling open requests were not coded when the context indicated that they were not addressing a problem of misunderstanding. These include *exclamations* (e.g., "seriously?!"), which express surprise without indicating misunderstanding, *question-formatted news receipts* [6] where the speaker is reacting to news (e.g., "really?"), and any *jokes or puns,* which intentionally express misunderstanding for humorous purposes.

3.2.2 Restricted requests

A restricted request specifies the nature or location of the problem within the trouble source. In terms of linguistic markers, restricted requests are often characterized by question markers (*who, what, where, which, why, when, how,* and *whose*) specifying part of the content of the trouble source (e.g., "who are you talking about?").

Example 2 illustrates an Other-initiated Self-completed repair containing a restricted request. The trouble source involves a comment about Tom Clancy's books, using an acronym to refer to a book written by the author. The Other initiates a repair by using a

restricted request that points to the source of misunderstanding (the acronym) followed by a question mark. The Self completes the repair by expanding the acronym and stating that this was the first novel written by Tom Clancy.

Example 2. Restricted request

Turn	Speaker	Example text	Repair components
1st	Self	Novel published 1993. Checks out. :)<br><br>Interesting: Clancy wrote it before HFRO, but agent and publishers more or less told him it was too dark. Eventually was published...sixth? or so of his books.	Trouble
2nd	Other	HFRO?	Initiation
3rd	Self	Hunt for Red October. His first published novel.	Completion

Restricted requests were only coded when a turn addresses a misunderstanding of the Self's perspective. Instances where a request is directed at a reported perspective in the trouble source were not considered repair initiations (e.g., "Why would Trump have done that?"). A question was not considered a restricted request if it was a *question-formatted news receipt* (e.g., "Why would anyone do that?"), a *joke* or *sarcastic statement* (e.g., "I wonder why you would do that ;)"), and a *rhetorical question* (e.g., "Who do you think?"). Example 3 illustrates an excluded question that contains the linguistic marker of a restricted request but could have arguably been performed for humorous purposes. The humorous intent was assumed as the reference to "New Zealand" in the trouble source is in reference to a place.

Example 3. Excluded question resembling restricted request

Turn	Speaker	Example text
1st	Self	Title: Spotted in Auckland, New Zealand. #diamondhands
2nd	Other	What is a New Zealand?
3rd	Self	It's like an Old Zealand, except it's a new one.

As repair initiations are an interactional achievement between interlocuters, excluded question types were coded as restricted request if the completion treated it as a genuine repair initiation. For instance, the second turn in example 4 is rhetorical, indicated using the word “lol” and the dismissive tone of the question. However, a second Other responding in the third turn treats the question as genuine, answering it as if it were non-rhetorical.

Example 4. Restricted request determined by completion

Turn	Speaker	Empirical example	Repair components
1st	Self	Title: Biden's first days in office were way better than I expected	Trouble
2nd	Other	lol who cares	Initiation
3rd	Other (2)	The majority of voters. That's why he was elected.	Completion

3.2.3 Restricted offers

A restricted offer is the most specific type of repair initiation as it provides a choice of completion for the Self to choose from. In terms of linguistic markers, restricted offers can be indicated by an “or” question (e.g., “Did you think it was red or black?”), a suggestion (e.g., “Did you mean 2023?”), or a partial repetition with a replacement (e.g., “You mean ‘going out in *London*’?”).

Example 5 illustrates an Other-initiated Self-completed repair containing a restricted offer. The trouble source involves the Self asking for help on understanding how “potential difference” works. The Other initiates the repair by asks for clarification on what type of potential difference, using a restricted offer by presenting two possible solutions (“gravitational” or “electrical”). In the third turn, the Self clarifies they meant electrical potential difference.

Example 5. Restricted offer

Turn	Speaker	Example text	Repair components
1st	Self	Title: ELI5: How exactly does potential difference work? I still don't get it. Body: Yeah the title..	Trouble
2nd	Other	Gravitational? Electrical?	Initiation
3rd	Self	Electrical please	Completion

Questions were not coded as restricted offers when they refer to a *reported perspective* in the trouble source (e.g., "Did Trump think he could win this"), when it is a *joke* or *sarcastic* statement (e.g., "You mean you *don't* want to get hurt?"), a *question-formatted news receipt* (You can't be serious?!"), a *personal insult* (e.g., "Are you dumb or just stupid?"), and a *rhetorical question* (e.g., "Do you REALLY believe that would work?"). For example, the Other responding in example 6 uses a phrase resembling a restricted offer ("It doesn't?") as a reaction to the previous post, following it up with an answer to the question within the same turn. This indicates they have understood the post and the statement is therefore not considered a repair initiation.

Example 6. Excluded question resembling restricted offer

Turn	Speaker	Example text
1st	Self	Title: When I was a little kid, I thought "This little piggy went to market." meant it went shopping. Body: It does not.
2nd	Other	Wait...what? It doesn't? Then what...........oh my!

Excluded question types were coded as restricted offers in cases where the third turn treated the question as an initiation, thereby completing the repair sequence. For instance, in example 7, the Self states they think the Harry Potter had a better soundtrack than narrative. The Other then uses a phrase structured as a restricted offer to express their disagreement.

However, the Self treats it as a repair initiation and elaborates on why they hold their perspective.

Example 7. Restricted offer determined by completion

Turn	Speaker	Example text	Repair components
1st	Self	The Harry Potter movies (especially the last few)	Trouble
2nd	Other	You thought the soundtrack was exceptionally better than the plot for Harry Potter? The blasphemy!	Initiation
3rd	Self	Yes. The soundtrack told the story where the books/film failed	Completion

3.2.4 Uncompleted initiations

There are cases where unambiguous attempts at repair may not have completions. Open requests were generally considered unambiguous as their inherent ambiguity renders them explicit indications of misunderstanding. For instance, there is evidence that the word "huh?" is found universally across cultures and languages [7], indicating it has the same pragmatic intent (repair) regardless of context. If a turn contained only the word "huh", it was coded as an open request, regardless of the completion.

Restricted requests were considered unambiguous when they make specific reference to the Self's perspective (e.g., "Why are you confused?") and when they repeat (e.g., "Why "confused"?) or rephrase/refer to the trouble source (e.g., "Why are you struggling with the idea?"). For instance, in example 8, the second turn receives no response yet is unambiguously asking for clarification on the Self's meaning in the previous turn.

Example 8. Restricted request without completion

Turn	Speaker	Example text	Repair components
1st	Self	Title: When I was a little kid, I thought "This little piggy went to market." meant it went shopping. Body: It does not.	Trouble
2nd	Other	Wait... what does it mean then? I must really be missing something	Initiation

Similarly, restricted offers were considered unambiguous when they refer to the Self's perspective (e.g., "You mean 2023?"), when they repeat (e.g., "Is it actually 2024?"), or rephrase/refer to (e.g., "Wasn't that the year after?") the trouble source. For instance, the Other in example 9 asks for clarification on what is meant by "UK style chips", which the Self has been referring to throughout the interaction. This initiation is anticipating an answer where, if that is the style of chips the Self is referring to, then the Other is positioning they agree with the statement.

Example 9. Restricted offer without completion

Turn	Speaker	Example text	Repair components
1st	Self	Having had both they're definitely different. Both delicious in their own right but I'd always rather have [UK style] chips	Trouble
2nd	Other	By proper UK style chips, are you referring to those stodgy fuckers that look like you've basically just cut a small potato into a few chunks and have that golden goodness of what is almost like fried mashed potato inside when you take a bite? Cause aye, those are awesome.	Initiation

An uncompleted initiation does not necessarily mean that no one has replied. It can also be when the next speakers do not attend to the clarification request and, instead, choose to ignore it. For instance, this ignoring of initiation has previously been associated with trolling in online interactions [8] and therefore justifies the coding of non-completions. We

do not explicitly code for these as they are self-evident from the statement of non-completion with a preceding turn.

3.3 Repair completions

Repair completions were coded as binary (completed or not completed). Who completed is determined by whether the completer is the source of the trouble source (Self) or not (Other). Our approach to coding completions was to determine whether the problem of understanding raised by the initiation is addressed in the next adjacent turn. A completion was coded when either the Self (see Example 2, 5, 7) or an Other acting in place of the Self (see Example 1, 4) addresses the initiation. Table 1 provides an overview of six possible indicators of completion. The Self may completely repeat the trouble source or partially repeat it, or rephrase it to double down on their original statement. The Self can also argue for their original statement through reframing it with a different perspective.

Table 1. Completion types. Trouble and initiation examples in the first row are designed to apply across the completion examples. Initiation 1 always relates to disconforming and initiation 2 conforming completions.

Completion type	Description	Example and conformity
	Conformity refers to whether the completion agrees with the premise of the initiation.	Trouble = "The year was 2022"<br>Initiation (I1) = "Was it not later?"<br>Initiation (I2) = "It's this year then?"
Full repetition	The content of the trouble source is repeated precisely.	Completion = "The year was 2022."
Partial repetition	Part of the content is referenced verbatim.	Completion = "It was 2022"

Rephrasing	The trouble source is rephrased without significant modification.	Completion = "It was before 2023"
Reframing	The trouble source is framed in a different perspective (modified).	Completion = "I was in Dubai, so it had to be then"
Explicit marker	The Self overtly refers to the intended meaning of the trouble source.	Completion = "I really did mean 2022"

Completions were not coded for if the answer provided did not address the misunderstanding raised in the initiation. This included instances where the initiation was *ignored by the person replying*, the response is a *secondary Other repeating or rephrasing the prior initiation* (e.g., "I also want to know what he meant"), or the *response is a joke or a pun*. For instance, example 10 highlights an instance where a restricted offer received a reply that was not coded as a completion. In the trouble source, the Self uses the word "retention", which leads the first Other to use a restricted offer to ask for clarification on what type of retention the Self is referring to. In the third turn, a second Other simply responds "yes" (in what is assumed to be an attempt at humour), thereby not acknowledging either of the options presented by the first Other and leaving the repair initiation uncompleted.

Example 10. Restricted offer with uncoded completion

Turn	Speaker	Example text	Repair components
1st	Self	Retention all day	Trouble
2nd	Other	Now, is that retention of streamers or retention of viewers?	Initiation
3rd	Other (2)	Yes.	

References

1. Schegloff EA, Jefferson G, Sacks H. The preference for self-correction in the organization of repair in conversation. Language. 1977;53: 361–382. doi:10.2307/413107

2. Dingemanse M, Kendrick KH, Enfield NJ. A coding scheme for other-initiated repair across languages. Open Linguist. 2016;2: 35–46. doi:10.1515/opli-2016-0002

3. Sacks H, Schegloff EA, Jefferson G. A simplest systematics for the organization of turn-taking for conversation. Language. 1974;50: 696–735. doi:10.2307/412243

4. Chandrasekharan E, Mattia S, Jhaver S, Charvat H, Bruckman A, Lampe C, et al. The internet’s hidden rules: An empirical study of Reddit norm violations at micro, meso, and macro scales. Proc ACM Hum-Comput Interact. 2018;2: 32:1-32:25. doi:10.1145/3274301

5. Dingemanse M, Roberts SG, Baranova J, Blythe J, Drew P, Floyd S, et al. Universal principles in the repair of communication problems. PLOS ONE. 2015;10: e0136100. doi:10.1371/journal.pone.0136100

6. Dingemanse M, Enfield NJ. Other-initiated repair across languages: Towards a typology of conversational structures. Open Linguist. 2015;1. doi:10.2478/opli-2014-0007

7. Dingemanse M, Torreira F, Enfield NJ. Is “huh?” a universal word? Conversational infrastructure and the convergent evolution of linguistic items. PLOS ONE. 2014;8: e78273. doi:10.1371/journal.pone.0078273

8. Paakki H, Vepsäläinen H, Salovaara A. Disruptive online communication: How asymmetric trolling-like response strategies steer conversation off the track. Comput Support Coop Work CSCW. 2021;30: 425–461. doi:10.1007/s10606-021-09397-1
