# Supplementary material for: Conversational repairs on Reddit: Widely initiated but often uncompleted
Source: PLoS One. 2025 Jan 2;20(1):e0316618. doi: 10.1371/journal.pone.0316618 (PMC11694982; doi:10.1371/journal.pone.0316618)
Supplement: S2 File — (PDF) [file pone.0316618.s002.pdf]

S2 File - Code for replicating results

This document details the code for replicating the results in the article titled:

"Conversational repairs on Reddit: Widely initiated but often uncompleted"

The document is divided into three main sections:

1. BERT Classifier validation

This section is concerned with validating a BERT [1] classifier used in scoring the Reddit comments for repair initiations. A repair initiation is operationalised as a clarification request (e.g., "what do you mean?") where one participant asks another to clarify something they have misunderstood. The BERT classifier was fine-tuned from the base model using the low code ktrain [2] package.

2. Analysis 1: Generalized mixed effects model

This section replicates the results of Analysis 1, which involves a generalized mixed effects model conducted using the lme4 R package [3]. In addition to the results presented in the article, it provides details of model selection and validation alongside.

3. Analysis 2: Survival analysis

This section replicates the results of Analysis 2, which involves a survival analysis conducted using the lifelines Python package [4].

Note on notebook usage.

For an easy accessible version of the notebook, click here and go to "S2_File.ipynb":

https://github.com/alexiamhe93/RedditRepairInitiations

This will open up the notebook in Google Colab and can be run directly without the need to sign into any account.

The below cells install R and all relevant packages required for analysis. If checking the BERT model, we advise using a GPU as running on a CPU alone will take a lot of time.

For accessing the text data

> <<< WARNING >>>

You should **only** access the text if you have (1) *permission from the copyright holders* (i.e., Reddit) and a (2) *host institution's permission via the relevant Research Ethics Committee* or other relevant ethics body.

> INSTRUCTIONS:

In order to access the text data, you must do the following:

1. Create a Reddit account if you do not have one already
2. Access the Reddit API
3. Create an app and fill out the form
4. Note your username, password, client ID, and client secret. Also define a "user_agent" - this is just an arbitrary name (no spaces) that should be unique and helps Reddit identify you (e.g., "username_RepairProject"). The client ID and client secret are obtained after creating an app in the Reddit API.
5. Input these details into the code below and run it. There may be problems at points due to the API having limits on how many downloads an individual can run. In this case, it may be best to split the data up and download in batches or add a cooldown to the code.

See the PRAW and Reddit API for more information.

> CODE FOR DOWNLOADING

```
import praw

reddit = praw.Reddit(client_id="your-client-id",client_secret="your-client-secret",
                     password="your-reddit-password",user_agent="your-unique-useragent",username="your-reddit-username")
reddit.read_only = True

def fetch_text(id):
  try:
    comment = reddit.comment(id=id)
    return comment.body
  except:
```

```
    try:
      submission = reddit.submission(id=id)
      out_text = " ".join(["Title:", submission.title, "Body:", submission.selftext])
      return out_text
    except:
      print("Problem fetching text - probably deleted.")
      return "[deleted]"

  turn_anon["text"] = turn_anon.id_turn.apply(fetch_text)
  mc_anon["text"] = mc_anon.id_turn.apply(fetch_text)
```

Install correct version of tensorflow for ktrain

If using in Google Colab, you may have to restart the session after loading it. Do not disconnect the runtime but instead just go to Runtime - Restart Session. There is no need to run the below cell after doing this.

In [ ]:
```
!pip install tensorflow==2.15.1 # You may have to restart session after doing this
```

Load data required for the analysis

In [1]:
```
import os
import numpy as np
# Plotting
import plotly.graph_objects as go
import plotly.express as px
from plotly.subplots import make_subplots
# For performing basic inferential statistics
import scipy.stats as stats
from collections import Counter
# General packages for data manipulation
import pandas as pd
pd.options.mode.chained_assignment = None  # default='warn'
from IPython.display import Image
```

In [2]:
```
# for downloading data
import requests, zipfile, io
r = requests.get('https://github.com/alexiamhe93/RedditRepairInitiations/blob/main/data.zip?raw=true' )
z = zipfile.ZipFile(io.BytesIO(r.content))
z.extractall()
```

In [3]:
```
## Load data
try:
 # Data for mixed effects model
 turn_df = pd.read_csv("turn_anon.csv")
 # Data for survival analysis
 threads_df = pd.read_csv("threads.csv")
 conv_df = pd.read_csv("conv.csv")
 # Manually coded data for BERT validation.
 mc_df = pd.read_csv("mc_anon.csv")
except:
 turn_df = pd.read_csv("data/turn_anon.csv")
 threads_df = pd.read_csv("data/threads.csv")
 conv_df = pd.read_csv("data/conv.csv")
 mc_df = pd.read_csv("data/mc_anon.csv")
```

In [ ]:
```
# This downloads the pretrained BERT model from dropbox - approx. 1/2 minutes depending on download speed
!wget -O initiation_BERT_model_087.zip https://www.dropbox.com/scl/fi/oxbjjrwim5sbfwcsl63n9/initiation_BERT_model_087.zip?rlkey=io2bbjiv7udjbs3u
!unzip initiation_BERT_model_087.zip
```

1. Validating BERT classifier

This section details the validation of the BERT classifier. It provides an accuracy report on the test set and an examination of misclassifications.

Note for replication: This part of the notebook cannot be fully replicated without the original text used to train and validate the BERT classifier. Unfortunately, due to data restrictions, this cannot be shared publicly. However, it is available upon request.

To mitigate the absence of the text data we have still provided the fine-tuned BERT model for exploration. It can be used to predict the category of a text (binary: repair initiation or not) by using the following code:

`predictor = ktrain.load_predictor('initiation_BERT_model_087')`

`predictor.predict("your text to classify")`

The statistics on the manual coded data (see 2.3.1 and 2.3.2) can be produced with the existing code.

1.1 Install and load packages

In [ ]:
```
# install ktrain for loading the model.
!pip install ktrain
```

In [ ]:
```
# For BERT classifier
import ktrain
from sklearn.metrics import classification_report,confusion_matrix,ConfusionMatrixDisplay
from sklearn.metrics import precision_recall_curve, auc, roc_auc_score
```

1.2 Load and prepare data

We split the manually coded data into component parts used for training and validating the BERT classifier. The training and test data excluded all first turns as these, by definition, cannot contain a repair initiation.

In [4]:
```
# remove first turns
n_MC = len(mc_df)
n_dia_MC = len(mc_df.id_conv.unique())
print(f"There are {n_MC} manually coded turns from {n_dia_MC} interactions.\n")
train_test = mc_df[mc_df["turn"]!=1]
# get the training and test data
train_mc = train_test[train_test.train_test == "train"]
print(f"The training set is {len(train_mc)} turns.")
test_mc = train_test[train_test.train_test == "test"]
print(f"The test set is {len(test_mc)} turns.")
training_data_size = 100*(len(train_mc) / (len(test_mc) + len(train_mc)))
print(f"Trained using {training_data_size:.2f}% of the manually coded data.\n")
# split initiations, trouble sources, and completions for further analysis
initiations = mc_df[mc_df.initiation==1]
print(f"There are {len(initiations)} repair initiations in the manually coded data.")
troubles = mc_df[(mc_df.initiation!=1) | (mc_df.completion != 1)]
completions = mc_df[mc_df.completion == 1]
print(f"There are {len(completions)} repair completions in the manually coded data.")
# get all the BERT coded data
BERT_codes = turn_df[turn_df.coded_by == "BERT"]
```

```
There are 3314 manually coded turns from 585 interactions.

The training set is 1983 turns.
The test set is 842 turns.
Trained using 70.19% of the manually coded data.

There are 355 repair initiations in the manually coded data.
There are 288 repair completions in the manually coded data.
```

1.3 Load the fine-tuned BERT model

We first load in the BERT model used for coding the full dataset. The model was fine-tuned from the base model and we include the weights for use in the analysis.

In [ ]:
```
# Load the trained BERT model for identifying initiations
predictor = ktrain.load_predictor('initiation_BERT_model_087')
```

```
/usr/local/lib/python3.10/dist-packages/keras/src/initializers/initializers.py:120: UserWarning: The initializer GlorotNormal is unseeded and being called m
ultiple times, which will return identical values each time (even if the initializer is unseeded). Please update your code to provide a seed to the initializer, o
r avoid using the same initializer instance more than once.
  warnings.warn(
```

In [ ]:
```
def calculate_accuracy(true_scores, test_scores):
  """
  For calculating predictive accuracy statistics for assessing classifier performance.
  """
  # Calculate AUC-ROC
  auc_roc = roc_auc_score(true_scores, test_scores)
  print(f'AUC-ROC: {auc_roc:.2f}\n')
  # Compute precision-recall curve
  precision, recall, thresholds = precision_recall_curve(true_scores, test_scores)
  # Calculate AUC-PR
  auc_pr = auc(recall, precision)
  print(f'AUC-PR: {auc_pr:.2f}\n')
  print(classification_report(true_scores,test_scores))
  conf_mx = confusion_matrix(true_scores, test_scores)
  disp = ConfusionMatrixDisplay(confusion_matrix=conf_mx)
  disp.plot()
```

Run the classifier on the test set and calculate accuracy statistics. Due to the inbalanced nature of the data (i.e., far fewer turns with initiations than turns without) the AUC PR (Area Under the Precision-Recall curve) is more appropriate than the conventional F1 score. The interpretation is the same, with higher values indicating a more accurate classifier.

In [ ]:
```
test_mc["BERT_scores"] = predictor.predict(test_mc.text.to_list())
test_mc["BERT_scores"] = test_mc.BERT_scores.apply(lambda x: 1 if x == "initiation" else 0)
true_scores = test_mc.initiation.astype(int).to_list()
test_scores = test_mc.BERT_scores.to_list()
calculate_accuracy(true_scores, test_scores)
```

AUC-ROC: 0.90

AUC-PR: 0.81

	precision	recall	f1-score	support
0	0.98	0.96	0.97	731
1	0.75	0.85	0.80	111
accuracy			0.94	842
macro avg	0.86	0.90	0.88	842
weighted avg	0.95	0.94	0.94	842

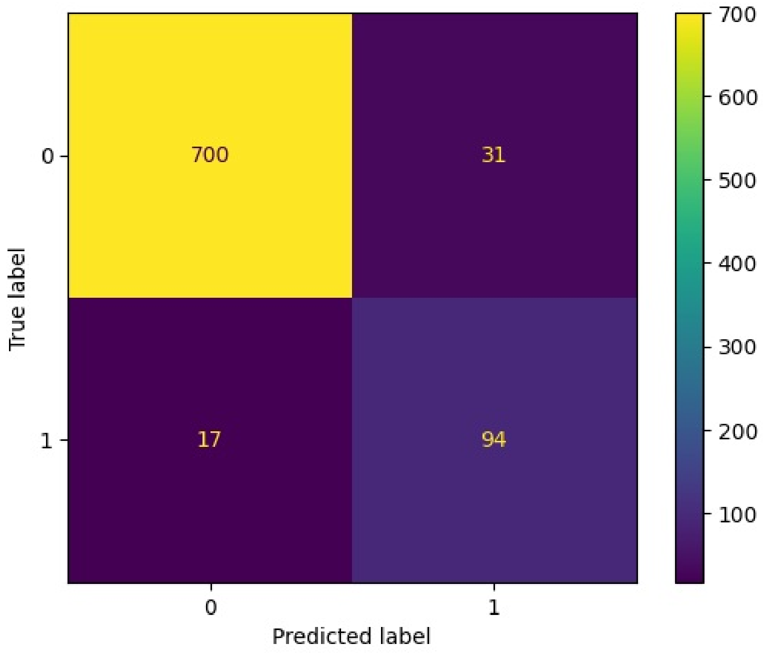

The AUC PR is moderately high (0.81) indicating the classifier is performing well. We can see it only makes 48 errors in classification over the 842 turns. It has a lower precision than recall indicating that the classifier produces more false positives than false negatives.

1.4 Examining misclassifications

We can now examine some misclassifications to see where the BERT classifier is having issues in performance.

In [ ]:
```
False_Positives = test_mc[(test_mc.BERT_scores == 1) & (test_mc.initiation == 0)]
False_Negatives= test_mc[(test_mc.BERT_scores == 0) & (test_mc.initiation == 1)]
print(f"There are {len(False_Positives)} false positives and {len(False_Negatives)} false negatives.")
```

There are 31 false positives and 17 false negatives.

1.4.1 False positives

In [ ]:
```
# Print the false positives
for fp in False_Positives.text.values:
  print(fp)
```

What about things that don't have husks? Like nuts or seeds that don't digest?
Is it weird I have never had a rhubarb in my life? What do they taste like?
What's the most direct way you know someone can modify their reality and abandon the usual conditionings of this system/matrix?
How were they not already there given the regime was committing genocide? Like, Aung San Suu Kyi is guilty of genocide.
People can be so terrible to each other. What did they achieve by killing her?
I'm a Freshman in college majoring in Electrical Engineering with a minor in Business Administration.

My dream job is CTO/VP/a similar upper corporate position. Do you have any advice on how I can make this dream reality?
What exactly is a project manager and how did you become one ?
Pick up some more AMC to offset that GME loss, sonnnnnnn!! How do people not get this?!
A whole 2.9 poop units? My god.
Why would they even do that? Seriously no one ever thinks about how the whole Jurassic park thing has zero affect on modern science
Less bugs and no useless dlc like fallout 4 i guess?
Hi babe! How was work today?
Why is this not my life?
What the fuck is that leaf doing? Can someone explain?
Crazy? No. Happy? Yes.
What? How can it be uncomfortable?
Why does this look like chicken?
Has it inspired you to pursue any other russian literature? I enjoyed The idiot and am reading to Anna karenina right now
This. If it's important, they'll leave a message. If not, why waste the energy?
Is it on k road lol?
Support? Nah most of these people are just genuinely retarded
How do you know? (Not doubting you just wondering)
Genuine question. As vaccines work by stimulating our own immune systems, why is a person surviving exposure to virus after being vaccinated contributing to selection pressure any more than an unvaccinated person surviving infection?
I want to try the Gulag Archipelago by Solzhenitsyn sometime soon. But I have a backlog of books already on-hand to read first, starting with one about the Rwandan genocide. Did you enjoy the Russian literature you just referenced? Anything in particular in these novels that really grabbed your interest?
Jean sandals?
Why do we have to do everything so backwards...?
Did you threaten to overrule him?
Do you have a car battery?
google it? i started my shit off with “i think” so i didn’t have to do the work for yalls. i got my news from this here reddit thingy
For kids? Uhh...
Would they be more likely to pass it than a food handler though? Everyone needs to wash their hands after touching the dog and before eating...

We can see that the classifier is misclassifying several types of questions that do not necessiraly indicate a request for clarification:

1. Question formatted news receipts and exclamations [5]. These questions involves a reaction to new information rather than a request for clarification (e.g., "A whole 2.9 poop units? My God.", "Support? Nah (...)".
2. Rhetorical questions. These questions are not meant to evoke a response and, instead, are meant to express the speaker's opinion (e.g., "Crazy? No. Happy? Yes.", "Why is this not my life?").
3. Questions taken out of context or quoted. These questions are reported speech that reflect the speaker's knowledge of the previous turn. For instance "Hi babe! How was work today?" is a reaction to a piece of media in the r/aww subreddit, meant to describe what the subject of the picture or video is saying.

We also see that some questions appeared to have been misclassified by the manual coders as they are unambiguously asking for clarification. For instance, "What exactly is a project manager and how did you become one?" is addressed to the previous speaker in the r/iama (ask me anything) subreddit. This subreddit involves asking the post creator any questions the commenter has in mind. In this case, the post creator stated they are a project manager and the next speaker is seeking clarification.

1.4.2 False negatives

In [ ]:
```
# Print the false positives
for fn in False_Negatives.text.values:
  print(fn)
```

Dang the downvotes... As a fan myself I agree. It seriously makes the whole experience hell when you witness the shithole of twitter. Kpop itself is marketed in a way that encourages competition between groups. There's voting by fans for awards(rigged lmao) and even the groups themselves. Like its crazy that a fanbase literally votes for who gets to be in the industry. Kpop also releases way too much content for the purpose of establishing a parasocial relationship with fans. This only causes fans to get even more cultish. The setup of Twitter really helps facilitate this behaviour. Anything controversial has an easier time rising to the top and having a civil discussion is nonexistent. I'm usually active in kpop communities here on Reddit and while there are disagreements, people are actually able to have civil discussions. Blatant hate gets deleted lol. It also depends on the subs you are in. Overall, yeah I've never had Twitter and I'm kind of scared to lol. Also, everyone is a kid on twitter??? Why does everyone just reveal that they are minors?????? I've seen comments like "I'm 11 :))". ????
Obsessed with you guys. Your ice cream is delicious. The freestyles are on point. How fun is it to donate and get to hear a freestyle on the live stream? From Britney Spears, to tiger king, to dnb youve killed it on all of my topics with skillz and hilarity. Just to die for! I appreciate all the details and personal touches. One of your cone heads somehow noticed that we both had a gold grill tooth while I was in the crowd. Ok now for a question- curious to learn more about your studio space. Does it have other uses? Do you rent it for events or other creative uses outside of fungineers needs? Cant wait for the day to see something live!
Commit yourself to the goal. Obtaining a skill and some kind of recognition professionally is a good thing. Getting a degree in engineering doesn't mean abandoning other stuff. Worst case, you get a degree and have more options. That is where it is generally better to position yourself - with more options; especially if you are just starting out. EDIT: I don't mean to say, get out of music. If you want to commit to it, then try to list what you are doing and think what is going to be needed? Sitting in a room all day? I suggest - that is not the answer. Are you actively (meaning hours going out) getting gigs? Are you marketing yourself? Are you networking? Do you have a mentor? What about your repertoire - what have you listened to recently to broaden your horizons? What specific areas are you developing musically (not thinking, actually doing!). How will you know you've achieved it? Don't imagine in broad terms (that is called fantasy). You need specific goals in developing your profession, music and skills with a timeframe to achieve them. Want to make music - what is popular now? Have you identified who can/will produce music for you? Have you tried to meet them? Why should people pay to listen to your music

? (Because YOU think it is good? Because YOU think "I am so creative", because YOU think "this is me being me"?) Don't want to focus on money or popularity? Then I hope you've got a plan to support yourself. Most people in ANY industry think about it as a BUSINESS.

I don't understand is why this is being presented as a new idea/discovery. As in anger being a common underlying feature in all forms of NPD. Kohut wrote about this stuff long before i was born. In essence narcissists, unable to acknowldge their own failures, blame other people for everything that goes wrong creating constant anger and resentment.

I prefer creating an account with the store and adding the gift card to my account. That way, it's stored on the store's system which pulls your balance away from the physical card which eliminates the risk for your card balance being stolen from the physical card. When you get to the checkout counter at the store, tell the clerk you have a registered account with the store and you have an available gift card balance you would like to use and more than likely you will be able to give them the card number and pin from your store phone app and usually even pull up a scannable digital copy of the card. I just feel like there's less risk doing that versus taking a snapshot. What if you accidentally delete the photo without backing it up to the cloud? What if you lose or damage your phone or get a new phone and the picture cannot be recovered?

>First, I think you are overestimating the attention span of kindergarten kids in terms of making fun of others. I don't know, man, there was a kid in my reception class who took playing Spiderman a tad too seriously just one time and the mockery hadn't ended until seven years later because we all went to new schools. >This works on the receiving end too, a rough day today does not necessarily have a long lasting impact Not necessarily, but I still remember shit that happened to me in reception (four years old btw in case you didn't know what reception year is) and my sister and a couple friends have bad memories from nursery (1-3) that they recall with astounding accuracy and clearly have affected their lives and not for the better. I think you've actually _understated_ those things. >Certainly the parent has a responsibility to make sure they are informed, and help them work through the possibilities. Again, I don't know. Kids do unwise stuff all the time. I'd say, that depending on the age, a parent has more responsibility than just being in an advisory capacity. I couldn't count the amount of decisions that I was certain were genius that my parents advised against, which I did anyway and suffered as a result. A 15 year old teen, sure. Be an advisor to them, a 4 year old, you gotta be more. >After that, the kids all just went about their day and no one really cared. Holy shit. What country is this in? I'm not saying I don't believe you, I mean, my comment was laced with my own anecdotes but where I grew up, this is straight up fantastical. Like the stuff of silly stories.

I really doubt there's been studies on the average intelligence of people that identify republican or democrat but there's huge amounts of data on educational level. People that have achieved higher levels of education are by far more likely to be democrat than republican. In other words, on average republicans are less educated than democrats. Is that a good proxy for intelligence? I don't know, I think it's as good as we could expect. Also it didn't used to be like this, up until fairly republicans had a lead in college grads but over this last several decades the party has just taken this anti-education stance. They don't trust science, they don't value education, they think it's liberal brainwashing, they don't value facts. Also, the stats are clear regarding religious leanings and political affiliation as well. There's a reason people think these things.

you have to slow down in order to go towards the sun... this is so strange to me but i trust you and it kinda makes sense. its like if you are in a car and you want to stop at the curb, you have to slow down to actually reach the curb otherwise you just go past it?

I have no idea what your title is saying

I'm not sure if you're joking, but I assume they mean "retarded", which adds a whole new layer of irony to their statement

Idk how you missed him riding on the wrong side of the road but ok.

I think you missed the previous part of that sentence, though I don't know what he meant by that either.

> If not however, you're courting more than just ridicule but bruising as well. Also when a kid is made fun of by another kid, they'll often stand up for themselves. Kids are tenacious like that. When a kid is made fun of by *everybody,* they just implode. I think this is where our experiences diverge, which naturally would lead to our very different takes. All of the elementary age violence I saw was pushing or a single punch, and was all done out of anger and not spite. And, having been the kid made fun of by everybody, it sucked, but then recess ended or the teacher got them back on track, and life moved on. It sometimes came back up here and there, but kids make fun of everything so it was very rare for one thing or target to persist. > Wounds scar for much longer than they hurt. You were likely shaped by those events even if you no longer feel them. I guess I can see that, but at what point is that just "you" and not a defect? I'm probably more shy than I would have been because of my childhood, but I'm also probably better than average at reading a room because of them. There are times I wish I was more outgoing, but there are also plenty of times that I have defused a social situation because I was the first to see it coming.

Then they need to fix the distribution, not the customer base. I can get you that there are tons of essential workers or other sick people who won't be able to get the vaccine right now but healthy people can because they're taking their parents or grandparents. If they weren't willing to take them before, that's bad. If the elderly weren't willing to go before, then why's that? There's no reason why Mass can't be giving the vaccine to more people in need if every other state is able to do it.

> even giving engineering very clear descriptions of exactly what the customer needs to do business, including UX and clear AC's, they still miss the mark at least 1/2 the time.

That's exactly what I said I hated PM trying to micromanage. The fact of the matter is that PMs are middle-men between people who don't exactly understand what they need and people who know how to take their needs and develop a solution for them, and PMs screw up the translation.

Then they try to micromanage the implementation of the solution without any idea how it works under the hood, and surprise the outcome is less than ideal because box in the engineers they build exactly what they're asked to build instead of actually trying to solve business problems with software.

The whole relationship is fucked. How is UX developing a solution before engineering even understands what the ask is?

Youre really fucking insecure dude and its honestly hella pathetic. I think you need to change your outlook on life and actually question whats important to you. Maybe question why youre so fucking anal about kissing and sex. Is it because youre grossed out or is it because your insecure self thinks that sex automatically means love and you cant stand the fact that she liked someone that isnt you. Its really sad that you feel the need to be special to someone instead of having dignity and pride with yourself.

Listen, man. We established that Yaniv is not acting in good faith. Who knows what their real motivations are - I think that using misgendering here as a way to question those motivations and highlight the chauvinistic nature of their conduct is reasonable. The n-word is a derogatory term for a whole race of people. It does not have a non-insulting meaning that is widely used - so using it as an *insult* is not okay. Clearly the word can and *is* still used in the context of comedy or in other vernacular variations. I mean FFS - you dont actually believe that calling someone by their birth gender (that conforms to their genetic sex) is equivalent to referring to a black person with a term that has had centuries of racist use - do you? >   context matters *Exactly* - here the context is Yaniv whose behavior is unconscionable.

The false negatives appear to be turns that are very long, contain a statement of misunderstanding, or a rhetorical question that was included due to having the next speaker treat it as a completion.

As examples of statements of misunderstanding, "I have no idea what your title is saying" and "I'm not sure if you're joking (...)", "I don't understand is why this is being presented (...)" and "I don't know what he meant by that either". In these cases, the classifier is having difficulty parsing them as they are not phrased as questions.

As an example of a rhetorical questions: "I prefer creating an account with the store and adding the gift card to my account. (...) I just feel like there's less risk doing that versus taking a snapshot. What if you accidentally delete the photo without backing it up to the cloud? What if you lose or damage your phone or get a new phone and the picture cannot be recovered?"

The questions posed in the above turn are indicating the speaker's position on what the previous speaker has said. However, since the previous speaker *treated* these as an initiation, they were coded by the manual coders.

2. Analysis 1: Generalized mixed effects model

In this section, we build a mixed effects model to explore the frequency of repair initiations across subreddits. We first run descriptive statistics relating to the frequency of initiations before performing model selection, running the final model to generate statistical results, and checking assumptions have been met.

Analysis 1 explores RQ1: "How does the distribution of Other-initiations vary across subreddits?"

2.1 Install R and load necessary packages

The installation of R and the necessary packages can take a while (approx. 20 minutes).

In [ ]:
```
# Install R and Rpy2
!apt-get install r-base
!pip install -q rpy2
packnames = ('lme4', "AICcmodavg","sjPlot", "MuMIn")
from rpy2.robjects.packages import importr
from rpy2.robjects.vectors import StrVector
utils = importr("utils")
utils.chooseCRANmirror(ind=1)
utils.install_packages(StrVector(packnames))
```

In [7]:
```
%load_ext rpy2.ipython
```

In [ ]:
```
%%R
install.packages("glmmTMB", type="source")
install.packages("DHARMa", type="source")
```

In [9]:
```
# Load necessary R package
%%R
# For running the generalized mixed effects model
library(lme4)
# For comparing models
library(AICcmodavg)
# For plotting residuals (assumptions)
library(sjPlot)
# For estimating the explained variance R^2
library(MuMIn)
```

```
WARNING:rpy2.rinterface_lib.callbacks:R[write to console]: Loading required package: Matrix

WARNING:rpy2.rinterface_lib.callbacks:R[write to console]:
Attaching package: 'AICcmodavg'


WARNING:rpy2.rinterface_lib.callbacks:R[write to console]: The following object is masked from 'package:lme4':

    checkConv


WARNING:rpy2.rinterface_lib.callbacks:R[write to console]:
Attaching package: 'MuMIn'


WARNING:rpy2.rinterface_lib.callbacks:R[write to console]: The following objects are masked from 'package:AICcmodavg':

    AICc, DIC, importance
```

In [10]:
```
# For scaling the numerical variables prior to analysis
from sklearn.preprocessing import StandardScaler
from scipy.stats import pearsonr
```

2.2 Load functions used for Analysis 1

In [11]:
```
# Functions for fetching the number of initiations from the data
def get_initiation_pct(df,initiation_column):
  n = len(df)
  repairs = df[initiation_column].sum()
  pct_repairs = round((repairs/n)*100,2)
  return n, f"{repairs} ({pct_repairs:.2f}%)"

def get_repair_stats(turn_df, threads_df, conv_df, group_col = "subreddit"):
  unique_subreddits = turn_df[group_col].unique()
  # turn level
```

```
    nTurns, pct_turn_repair = get_initiation_pct(turn_df, "initiation")
    # thread level
    nThreads, pct_thread_repair = get_initiation_pct(threads_df, "contains_initiation")
    # interaction level
    nConv, pct_conv_repair = get_initiation_pct(conv_df, "contains_initiation")
    report = f"""
    Of {nTurns} turns, {pct_turn_repair} were repair initiations.
    Of {nThreads} threads, {pct_thread_repair} contained at least one repair initiations.
    Of {nConv} interactions, {pct_conv_repair} contained at least one repair initiation.
    """
    print(report)


def plot_initiations_subreddits(turn_df, conv_df, value_col='initiation', group_col='subreddit'):
    def prepare_data(df, group_col, value_col, category_name):
        """Helper function to prepare data for plotting."""
        sub_df = df.groupby(group_col)[value_col].mean().reset_index()
        sub_df.columns = ["Subreddit", "Initiation Rate"]
        sub_df["Category"] = category_name
        sub_df["Rank"] = sub_df["Initiation Rate"].rank(method='first', ascending=False)
        return sub_df

    # Prepare data for each category
    conv_data = prepare_data(conv_df, group_col, value_col, "Interactions")
    turn_data = prepare_data(turn_df, group_col, value_col, "Turns")

    # Combine dataframes
    combined_df = pd.concat([conv_data, turn_data])

    # Add initiation rate from interactions for color mapping
    color_mapping_conv = conv_data.set_index("Subreddit")["Initiation Rate"].to_dict()
    color_mapping_turn = turn_data.set_index("Subreddit")["Initiation Rate"].to_dict()

    # Create bump chart
    fig = px.line(combined_df, x="Category", y="Rank", color="Subreddit",
                  line_shape='linear', markers=True,
                  labels={"Rank": "Rank", "Category": "Category"},)
                  #width=800,  # Set width suitable for Word
                  #height=600) # Set height suitable for Word


    # Update trace colors based on initiation rate for interactions
    for trace in fig.data:
        subreddit = trace.name
        initiation_rate = color_mapping_conv.get(subreddit, 0)  # Get initiation rate, default to 0 if not found
        # Map initiation rate to color using Viridis scale
        line_color = px.colors.sample_colorscale('Viridis', initiation_rate)[0]  # Get the first element from the list
        trace.line.color = line_color
        trace.marker.color = line_color  # Make sure markers have the same color

    # Invert y-axis to have rank 1 at the top
    fig.update_yaxes(autorange="reversed", dtick=1)  # Add individual ticks for each rank

    # Remove legend
    fig.update_layout(showlegend=False)

    # Add annotations for each subreddit at the leftmost position
    for subreddit in combined_df["Subreddit"].unique():
        # Get the first point for each subreddit
        first_point = combined_df[combined_df["Subreddit"] == subreddit].iloc[0]
        last_point = combined_df[(combined_df["Subreddit"] == subreddit) & (combined_df["Category"] == "Turns")].iloc[0]
        initiation_rate_conv = color_mapping_conv[subreddit] * 100  # Convert to percentage
        initiation_rate_turn = color_mapping_turn[subreddit] * 100  # Convert to percentage for Turns

        # Set the annotation x position slightly to the left of the first point
        annotation_x_pos = -0.05  # Use a fixed offset to the left of the first category

        fig.add_annotation(
            x=annotation_x_pos,
            y=first_point["Rank"],
            text=f"{subreddit} ({initiation_rate_conv:.2f}%)",  # Add initiation rate percentage from interactions
            showarrow=False,
            xanchor="right",  # Position the text to the left of the point
            yanchor="middle",
            xshift=-5  # Additional shift to the left
        )

        # Add annotation for each subreddit at the rightmost position (Turns)
        annotation_x_pos_right = 1.05  # Adjusted for only two categories
        fig.add_annotation(
            x=annotation_x_pos_right,
            y=last_point["Rank"],
            text=f"{subreddit} ({initiation_rate_turn:.2f}%)",  # Add initiation rate percentage from Turns
```

```
        showarrow=False,
        xanchor="left",  # Position the text to the right of the point
        yanchor="middle",
        xshift=5  # Additional shift to the right
    )
fig.update_xaxes(title="")
fig.update_layout(font=dict(family="Arial", size=12))
fig.update_layout(width=800, height=1000)
# Show plot
fig.show()




def get_initiation_stats(df, initiation_column):
    n = len(df)
    repairs = df[initiation_column].sum()
    pct_repairs = round((repairs / n) * 100, 2) if n > 0 else 0
    return n, repairs, pct_repairs

def get_repair_stats2(turn_df,conv_df, group_col="subreddit"):
    unique_subreddits = turn_df[group_col].unique()

    # Overall stats
    n_turns, repairs_turn, pct_turn_repair = get_initiation_stats(turn_df, "initiation")
    n_conv, repairs_conv, pct_conv_repair = get_initiation_stats(conv_df, "contains_initiation")

    data = {
        "subreddit": ["All subreddits"],
        "N (conv)": [n_conv],
        "Repairs (conv)": [repairs_conv],
        "Pct Repairs (conv)": [pct_conv_repair],
        "N (turn)": [n_turns],
        "Repairs (turn)": [repairs_turn],
        "Pct Repairs (turn)": [pct_turn_repair],
    }

    # Stats for each subreddit
    for sub in unique_subreddits:
        n_turns, repairs_turn, pct_turn_repair = get_initiation_stats(turn_df[turn_df[group_col] == sub], "initiation")
        n_conv, repairs_conv, pct_conv_repair = get_initiation_stats(conv_df[conv_df[group_col] == sub], "contains_initiation")

        data["subreddit"].append(sub)
        data["N (conv)"].append(n_conv)
        data["Repairs (conv)"].append(repairs_conv)
        data["Pct Repairs (conv)"].append(pct_conv_repair)
        data["N (turn)"].append(n_turns)
        data["Repairs (turn)"].append(repairs_turn)
        data["Pct Repairs (turn)"].append(pct_turn_repair)

    outdf = pd.DataFrame(data)
    return outdf
```

2.3 Descriptive statistics

We first look at the proportion of initiations across our three levels of analysis: interactions, threads, and turns. An interaction is the entire post-comment set, a thread is one branch in the post comments set (organised by the reply-to function), and the turn level is across the entire subreddit.

In [12]:

```
get_repair_stats(turn_df, threads_df, conv_df)
```

Of 157667 turns, 26242 (16.64%) were repair initiations.
Of 92441 threads, 34642 (37.47%) contained at least one repair initiations.
Of 3750 interactions, 2193 (58.48%) contained at least one repair initiation.

We now examine how the ranks of subreddits vary based on the proportion of initiations at the interaction and turn levels. The interaction level is the number of interactions containing an initiation (n=150 for each subreddit) and the turn level is the number of turns that *are* initiations.

In [13]:

```
conv_df["initiation"] = conv_df.contains_initiation
plot_initiations_subreddits(turn_df, conv_df)
```

In [25]:

```
Image('Initiation_frequency_ranks.png')
```

Out[25]:

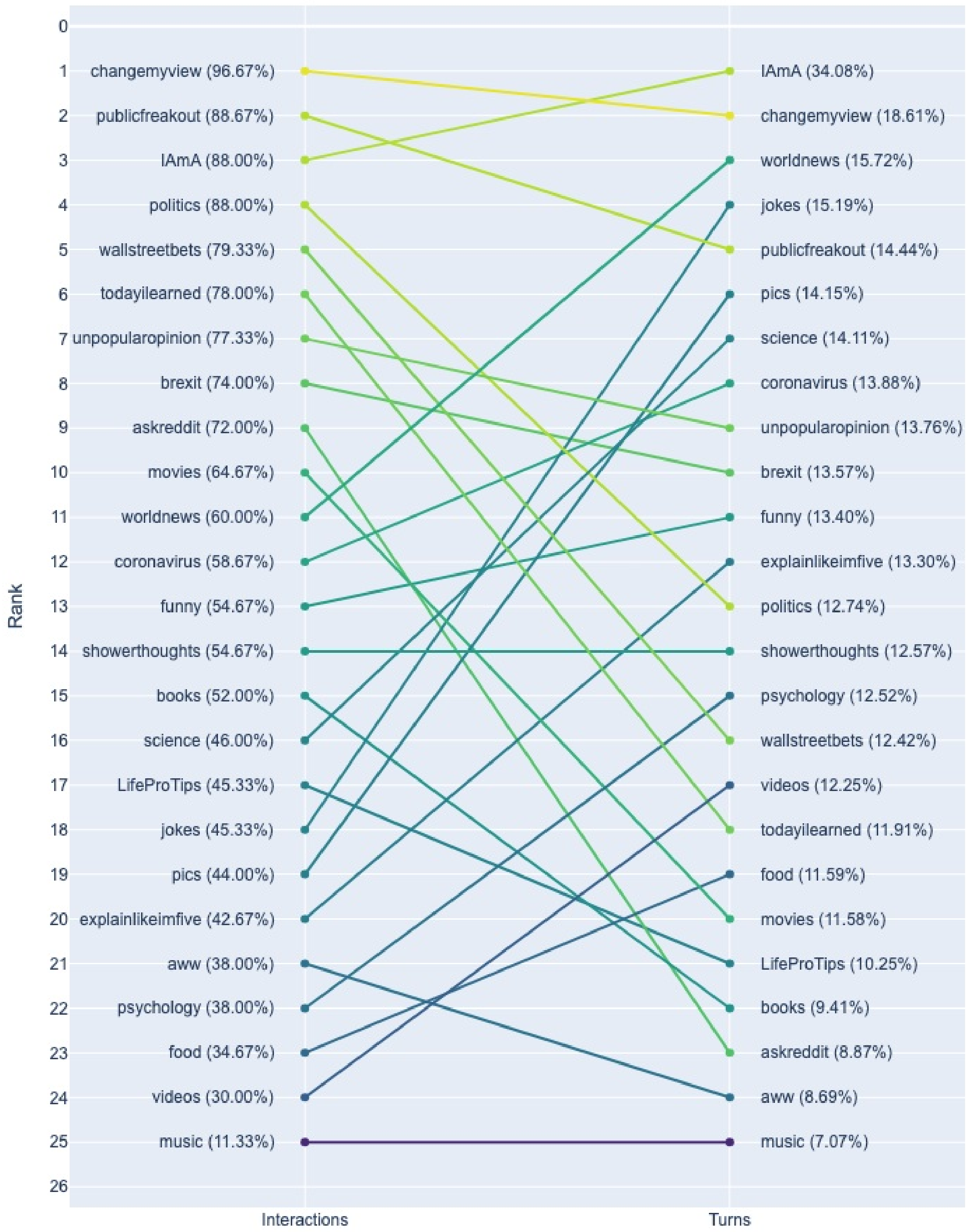

To examine the exact numbers for this graph, we put them into a table:

In [ ]:

```
rstats_subs = get_repair_stats2(turn_df, conv_df)
rstats_subs_mean = rstats_subs[["Pct Repairs (conv)","Pct Repairs (turn)"]].describe()
rstats_subs_mean.columns = ["Interaction","Turn"]
rstats_subs
```

Out[ ]:

	subreddit	N (conv)	Repairs (conv)	Pct Repairs (conv)	N (turn)	Repairs (turn)	Pct Repairs (turn)
0	All subreddits	3750	2193	58.48	157667	26242	16.64
1	explainlikeimfive	150	64	42.67	2082	277	13.30
2	politics	150	132	88.00	14283	1820	12.74
3	science	150	69	46.00	3635	513	14.11
4	todayilearned	150	117	78.00	9111	1085	11.91
5	movies	150	97	64.67	8201	950	11.58
6	wallstreetbets	150	119	79.33	10498	1304	12.42
7	askreddit	150	108	72.00	8025	712	8.87
8	pics	150	66	44.00	3025	428	14.15
9	coronavirus	150	88	58.67	4668	648	13.88
10	aww	150	57	38.00	4015	349	8.69
11	showerthoughts	150	82	54.67	2108	265	12.57
12	worldnews	150	90	60.00	8971	1410	15.72
13	videos	150	45	30.00	3845	471	12.25
14	changemyview	150	145	96.67	9485	1765	18.61
15	unpopularopinion	150	116	77.33	4898	674	13.76
16	psychology	150	57	38.00	1574	197	12.52
17	funny	150	82	54.67	4419	592	13.40
18	LifeProTips	150	68	45.33	1893	194	10.25
19	IAmA	150	132	88.00	27038	9214	34.08
20	music	150	17	11.33	835	59	7.07
21	jokes	150	68	45.33	2672	406	15.19
22	food	150	52	34.67	1398	162	11.59
23	brexit	150	111	74.00	4982	676	13.57
24	books	150	78	52.00	4772	449	9.41
25	publicfreakout	150	133	88.67	11234	1622	14.44

2.3.1 Preference for specificity

In the manual coded data, we tracked the different types of repair intiations. These were coded for specificity [6]. Open requests (e.g., "huh?") are the least specific type, leaving open what the misunderstanding is about. Restricted requests (e.g., "what do you mean by that?"), the second most specific type, indicates the location of the misunderstanding. Restricted offers (e.g., "did you mean X or Y?"), the most specific type, provides a possible solution to the misunderstanding.

We use a Chi-Square test to determine whether the differences between the types is statistically significant.

In [ ]:

```
def Chi2(categorical_lst, alpha = 0.01):
    """
    Chi-square test for identifying significant differences between groups.
    """
    # Count the frequency of each category
    observed_freq = Counter(categorical_lst)
    # Get the observed frequencies in the order of categories
    observed_values = list(observed_freq.values())
    # If you have an expected distribution, you can define it. For example, equal distribution:
    expected_values = [len(categorical_lst) / len(observed_freq)] * len(observed_freq)
    # Perform the Chi-Square Goodness of Fit test
    chi2_stat, p_value = stats.chisquare(f_obs=observed_values, f_exp=expected_values)
    print(f"Chi-Square Statistic: {chi2_stat:.2f}")
    print(f"P-Value: {p_value:.5f}")
    # Interpretation
    if p_value < alpha:
        print(f"The frequency of the categories is significantly different (sig level = {alpha}).")
    else:
        print(f"The frequency of the categories is not significantly different (sig level = {alpha}).")
```

In [ ]:

```
initiations = mc_df[mc_df.initiation==1]
```

```
pd.concat([initiations.clarification_type.value_counts().reset_index(),
          initiations.clarification_type.value_counts(normalize=True).reset_index()], axis=1)
```

Out[ ]:

	clarification_type	count	clarification_type	proportion
**0**	restricted offer	189	restricted offer	0.532394
**1**	restricted request	158	restricted request	0.445070
**2**	open request	8	open request	0.022535

In [ ]:
```
Chi2(initiations.clarification_type.to_list())
```

```
Chi-Square Statistic: 158.37
P-Value: 0.00000
The frequency of the categories is significantly different (sig level = 0.01).
```

2.3.2 Preference for self completion

We also tracked the completions for the initiations in the manually coded data. We also looked at who completed the repair. We can test to see whether there is a statistically significant difference in who completed the repair.

In [ ]:
```
pd.concat([mc_df.completed_by.value_counts().reset_index(),
          mc_df.completed_by.value_counts(normalize=True).reset_index()], axis=1)
```

Out[ ]:

	completed_by	count	completed_by	proportion
**0**	Self	187	Self	0.526761
**1**	Other	102	Other	0.287324
**2**	No_completion	66	No_completion	0.185915

In [ ]:
```
Chi2(mc_df.completed_by.to_list())
```

```
Chi-Square Statistic: 7314.14
P-Value: 0.00000
The frequency of the categories is significantly different (sig level = 0.01).
```

In addition to the manual coding, we tracked whether how many replies each comment/post received and whether these replies originated from the Self (author of the previous turn) or Other (any other author). This allowed us to estimate the number of completions in the fully coded dataset.

In [ ]:
```
# Calculate the number of all initiations
all_initiations = len(turn_df[turn_df['initiation'] == 1])
# Calculate the number of uncompleted initiations
uncompleted = len(turn_df.loc[(turn_df['initiation'] == 1) & (turn_df['Self'] == 0) & (turn_df['Other'] == 0)])
pct_uncompleted = round((uncompleted / all_initiations) * 100, 2)
# Calculate the number of self-completed only initiations
self_completed_only = len(turn_df.loc[(turn_df['initiation'] == 1) & (turn_df['Self'] != 0) & (turn_df['Other'] == 0)])
pct_Self_completed = round((self_completed_only / all_initiations) * 100, 2)
# Calculate the number of both-completed repairs
both_completed_repairs = len(turn_df.loc[(turn_df['initiation'] == 1) & (turn_df['Self'] != 0) & (turn_df['Other'] != 0)])
pct_both_completed = round((both_completed_repairs / all_initiations) * 100, 2)
# Calculate the number of other-completed only initiations
other_completed_only = len(turn_df.loc[(turn_df['initiation'] == 1) & (turn_df['Self'] == 0) & (turn_df['Other'] != 0)])
pct_other_completed = round((other_completed_only / all_initiations) * 100, 2)

print(f"""
There are {all_initiations} in the dataset.
- {self_completed_only} ({pct_Self_completed}%) were replied to by Self only.
- {both_completed_repairs} ({pct_both_completed}%) were replied to by both Self and Other(s).
- {other_completed_only} ({pct_other_completed}%) were replied to by Other(s) only.
- {uncompleted} ({pct_uncompleted}%) had no replies and thus remained uncompleted.
""")
```

```
There are 26242 in the dataset.
- 7308 (27.85%) were replied to by Self only.
- 1746 (6.65%) were replied to by both Self and Other(s).
- 5432 (20.7%) were replied to by Other(s) only.
- 11756 (44.8%) had no replies and thus remained uncompleted.
```

2.4 Generalized mixed effects model

Before exploring the generalized mixed effects model, it is necessary to prepare the dataset. We scale all of the numerical variables (word count, previous turn word count, and time since last turn). This helps fulfil the model assumptions. We remove all of the first turns as these can never be initiations and therefore adds unecessary noise to the analysis.

The variables are as follows:

`CR` = whether a turn is an initiation. This is our response variable.

`t` = turn number of the comment

`wc` = the turn's word count

`prev_wc` = the previous turn's word count

`reply_t` = the time (seconds) since the previous turn

`sub` = the subreddit from which the turn belongs

`conv` = the interaction from which the turn was sampled from.

Threads are not included as turns can belong to multiple threads at once.

In [ ]:
```
turn_df2 =turn_df.copy()
turn_df2 = turn_df2[turn_df2.turn != 1]
turn_df2 = turn_df2[["id_turn","id_conv","subreddit","turn","initiation","word_count","prev_word_count","time_since_last_comment"]]
turn_df2 = turn_df2.rename(columns = {"subreddit":"sub",
                                      "initiation": "CR",
                                      "word_count":"wc",
                                      "prev_word_count":"prev_wc",
                                      "time_since_last_comment":"reply_t",
                                      "turn":"t",
                                      "id_conv":"conv"})
response = "CR"
fixed_effects = ["t", "wc", "prev_wc","reply_t"]
random_effects = ["sub","conv"]
mme_cols = [response] + fixed_effects + random_effects
mme_df = turn_df2[mme_cols]
# Log center the wordcount to scale the variable
scaler = StandardScaler()
mme_df[["wc","prev_wc","reply_t"]] = scaler.fit_transform(mme_df[["wc","prev_wc","reply_t"]])
```

Now the dataset is created, we can load it into R for analysis.

In [ ]:
```
mme_df = mme_df.dropna()
mme_df.iteritems = mme_df.items
```

In [ ]:
```
%%R -i mme_df
data <- mme_df
```

2.4.1 Model selection

For identifying the best model, we first select the best intercept model using the AIC statistic. The intercept model only contains random effects, i.e., the categorical variables we expect to have different slopes (subreddit and interactions).

In [ ]:
```
%%R
f_i0 <- "CR ~ 1 + (1|sub)"
f_i1 <- "CR ~ 1 + (1|sub) + (1|conv)"

# Run models
im0 <- glmer(f_i0, data = data, family="binomial")
im1 <- glmer(f_i1, data = data, family="binomial")

model_lst = list(im0,im1)
mod_names = c(f_i0,f_i1)
myaicc<-as.data.frame(AICcmodavg::aictab(cand.set= model_lst, modnames=mod_names))[,-c(5,7)]
myaicc$eratio<-max(myaicc$AICcWt)/myaicc$AICcWt
out <- data.frame(Model=myaicc[,1],round(myaicc[, 2:7],4))
out
```

```
                        Model K     AICc Delta_AICc AICcWt Cum.Wt        eratio
2 CR ~ 1 + (1|sub) + (1|conv) 3 132781.7      0.000      1      1  1.000000e+00
1            CR ~ 1 + (1|sub) 2 134140.2   1358.495      0      1 9.849188e+294
```

The best intercept model is the more complex one with both subreddit and interactions as random effects. We can double check this is the case with an ANOVA:

In [ ]:
```
%%R
anova(im0,im1)
```

```
Data: data
Models:
im0: CR ~ 1 + (1 | sub)
im1: CR ~ 1 + (1 | sub) + (1 | conv)
    npar    AIC     BIC logLik deviance  Chisq Df Pr(>Chisq)
im0    2 134140 134160 -67068   134136
im1    3 132782 132812 -66388   132776 1360.5  1  < 2.2e-16 ***
---
Signif. codes:  0 '***' 0.001 '**' 0.01 '*' 0.05 '.' 0.1 ' ' 1
```

Now we add the fixed effects into the model. We add these in without interactions at present to see if their addition lowers the interpretability of the model. We do this with the AIC and BIC statistics. Lower AIC and BIC values are preferred because they indicate a model that strikes a better balance between accuracy (good fit to the data) and parsimony (simplicity of the model). We use a backwards selection process, starting with the most complex model and then examining simpler models.

In [ ]:

```
%%R
# All covariates
f_mx1 = "CR ~ 1 + t + wc + prev_wc + reply_t + (1|conv) + (1|sub)"
# Removed word counts
f_mx2 = "CR ~ 1 + t + reply_t + (1|conv) + (1|sub)"
# Removed turns
f_mx3 = "CR ~ 1 + wc + prev_wc + reply_t + (1|conv) + (1|sub)"
# Removed response time
f_mx4 = "CR ~ 1 + t + wc + prev_wc + (1|conv) + (1|sub)"
mx_1 = glmer(f_mx1, data = data, family="binomial") #
mx_2 = glmer(f_mx2, data = data, family="binomial") #
mx_3 = glmer(f_mx3, data = data, family="binomial") #
mx_4 = glmer(f_mx4, data = data, family="binomial") #

model_lst = list(im1,mx_1,mx_2, mx_3,mx_4)
mod_names = c("intercept","mx_1","mx_2", "mx_3","mx_4")
myaicc<-as.data.frame(AICcmodavg::aictab(cand.set= model_lst, modnames=mod_names))[,-c(5,7)]
myaicc$eratio<-max(myaicc$AICcWt)/myaicc$AICcWt
out <- data.frame(Model=myaicc[,1],round(myaicc[, 2:7],4))
out
```

```
      Model K     AICc Delta_AICc AICcWt Cum.Wt        eratio
5      mx_4 6 128446.1     0.0000 0.7234 0.7234  1.000000e+00
2      mx_1 7 128448.1     1.9224 0.2766 1.0000  2.614900e+00
4      mx_3 6 128972.7   526.5986 0.0000 1.0000 2.235749e+114
3      mx_2 5 131503.4  3057.2795 0.0000 1.0000           Inf
1 intercept 3 132781.7  4335.5387 0.0000 1.0000           Inf
```

The reply time seems to make the model worse. We can check to see if the most complex model (1) is performing the same as the model without reply time (4):

In [ ]:

```
%%R
anova(mx_1, mx_4)
```

```
Data: data
Models:
mx_4: CR ~ 1 + t + wc + prev_wc + (1 | conv) + (1 | sub)
mx_1: CR ~ 1 + t + wc + prev_wc + reply_t + (1 | conv) + (1 | sub)
     npar    AIC    BIC logLik deviance  Chisq Df Pr(>Chisq)
mx_4    6 128446 128506 -64217   128434
mx_1    7 128448 128518 -64217   128434 0.0777  1     0.7804
```

The two models explain the same amount of information. We can therefore move onto the second round of backwards selection:

In [ ]:

```
%%R
# Without word counts
f_mx6 = "CR ~ 1 + t + (1|conv) + (1|sub)"
# Removed turns
f_mx7 = "CR ~ 1 + wc + prev_wc + (1|conv) + (1|sub)"
mx_6 = glmer(f_mx6, data = data, family="binomial") #
mx_7 = glmer(f_mx7, data = data, family="binomial") #

model_lst = list(im1,mx_4,mx_6, mx_7)
mod_names = c("intercept","mx_4","mx_6", "mx_7")
myaicc<-as.data.frame(AICcmodavg::aictab(cand.set= model_lst, modnames=mod_names))[,-c(5,7)]
myaicc$eratio<-max(myaicc$AICcWt)/myaicc$AICcWt
out <- data.frame(Model=myaicc[,1],round(myaicc[, 2:7],4))
out
```

```
      Model K     AICc Delta_AICc AICcWt Cum.Wt        eratio
2      mx_4 6 128446.1     0.0000      1      1  1.000000e+00
4      mx_7 5 128971.2   525.0562      0      1 1.033989e+114
3      mx_6 4 131502.9  3056.7367      0      1           Inf
1 intercept 3 132781.7  4335.5387      0      1           Inf
```

The more complex model from the previous selection seems best; we can double check with an anova:

In [ ]:
```
%%R
anova(mx_7, mx_4)
```

```
Data: data
Models:
mx_7: CR ~ 1 + wc + prev_wc + (1 | conv) + (1 | sub)
mx_4: CR ~ 1 + t + wc + prev_wc + (1 | conv) + (1 | sub)
     npar    AIC    BIC logLik deviance  Chisq Df Pr(>Chisq)
mx_7    5 128971 129021 -64481   128961
mx_4    6 128446 128506 -64217   128434 527.06  1  < 2.2e-16 ***
---
Signif. codes:  0 '***' 0.001 '**' 0.01 '*' 0.05 '.' 0.1 ' ' 1
```

The more complex model is chosen with response time removed:

```
CR ~ 1 + t + wc + prev_wc + (1|conv) + (1|sub)
```

We can now add interaction effects into the model. The word count of the turn would, theoretically, be related to the word count of the previous turn. Therefore, we assume an interaction between them. We run this model then check it against our previous best model:

In [ ]:
```
%%R
# With the interaction
f_mx8 = "CR ~ 1 + t + wc + prev_wc + wc:prev_wc + (1|conv) + (1|sub)"
mx_8 = glmer(f_mx8, data = data, family="binomial")
```

In [ ]:
```
%%R
model_lst = list(im1,mx_4,mx_8)
mod_names = c("intercept","mx_4","mx_8")
myaicc<-as.data.frame(AICcmodavg::aictab(cand.set= model_lst, modnames=mod_names))[,-c(5,7)]
myaicc$eratio<-max(myaicc$AICcWt)/myaicc$AICcWt
out <- data.frame(Model=myaicc[,1],round(myaicc[, 2:7],4))
out
```

```
      Model K     AICc Delta_AICc AICcWt Cum.Wt   eratio
3      mx_8 7 128424.3     0.0000      1      1     1.00
2      mx_4 6 128446.1    21.8292      0      1 54972.95
1 intercept 3 132781.7  4357.3679      0      1      Inf
```

In [ ]:
```
%%R
anova(mx_8, mx_4)
```

```
Data: data
Models:
mx_4: CR ~ 1 + t + wc + prev_wc + (1 | conv) + (1 | sub)
mx_8: CR ~ 1 + t + wc + prev_wc + wc:prev_wc + (1 | conv) + (1 | sub)
     npar    AIC    BIC logLik deviance  Chisq Df Pr(>Chisq)
mx_4    6 128446 128506 -64217   128434
mx_8    7 128424 128494 -64205   128410 23.829  1  1.053e-06 ***
---
Signif. codes:  0 '***' 0.001 '**' 0.01 '*' 0.05 '.' 0.1 ' ' 1
```

The more complex model with the interaction term performs the best:

```
CR ~ 1 + t + wc + prev_wc + wc:prev_wc + (1 | conv) + (1 | sub)
```

A mixed effects model lets us add in random slopes, which allows the variables to vary according to the mixed effects.

Model slopes are added based on the assumption that the fixed effect varies according to the grouping variable. In this case, we pick the word count

Adding random slopes reduces the chance of type 1 errors, [7] but can make the results more difficult to interpret.

We only include word count as a random slope for interactions and subreddit to show the comparison between them. The other variables were included in a previous iteration but none of these models converged.

In [ ]:
```
%%R
# Turn and word counts as random slope
f_rs1  = "CR ~ 1 + t + wc + prev_wc + wc:prev_wc + (wc|conv) + (1|sub)"
# Turn only
f_rs2  = "CR ~ 1 + t + wc + prev_wc + wc:prev_wc + (1 |conv) + (wc|sub)"

m_rs1 = glmer(f_rs1, data = data, family="binomial") # NO CONVERGENCE
m_rs2 = glmer(f_rs2, data = data, family="binomial")
model_lst = list(im1,mx_4,m_rs1, m_rs2)
mod_names = c("intercept","mx_4","m_rs1","m_rs2")
myaicc<-as.data.frame(AICcmodavg::aictab(cand.set= model_lst, modnames=mod_names))[,-c(5,7)]
myaicc$eratio<-max(myaicc$AICcWt)/myaicc$AICcWt
out <- data.frame(Model=myaicc[,1],round(myaicc[, 2:7],4))
out
```

```
      Model K     AICc Delta_AICc AICcWt Cum.Wt       eratio
4    m_rs2 9 128146.0     0.0000 0.8456 0.8456 1.000000e+00
3    m_rs1 9 128149.4     3.4013 0.1544 1.0000 5.477400e+00
2     mx_4 6 128446.1   300.1114 0.0000 1.0000 1.473507e+65
1 intercept 3 132781.7  4635.6501 0.0000 1.0000          Inf
```

The word count in interactions as a random slope failed to converge. We can now check whether the word count in subreddits as random slopes model (f_rs2) performs better than the model without random slopes:

In [ ]:
```
%%R
anova(mx_4,m_rs2)
```

```
Data: data
Models:
mx_4: CR ~ 1 + t + wc + prev_wc + (1 | conv) + (1 | sub)
m_rs2: CR ~ 1 + t + wc + prev_wc + wc:prev_wc + (1 | conv) + (wc | sub)
      npar    AIC    BIC logLik deviance  Chisq Df Pr(>Chisq)
mx_4     6 128446 128506 -64217   128434
m_rs2    9 128146 128236 -64064   128128 306.11  3  < 2.2e-16 ***
---
Signif. codes:  0 '***' 0.001 '**' 0.01 '*' 0.05 '.' 0.1 ' ' 1
```

The random slopes model performs better than the one without and, as such, we include these in the final model.

2.5 Analysis 1 results

Now that we have selected the best model, we can examine the results:

In [ ]:
```
%%R
summary(m_rs2)
```

```
Generalized linear mixed model fit by maximum likelihood (Laplace
  Approximation) [glmerMod]
 Family: binomial  ( logit )
Formula: CR ~ 1 + t + wc + prev_wc + wc:prev_wc + (1 | conv) + (wc | sub)
   Data: data

     AIC      BIC   logLik deviance df.resid
128146.0 128235.5 -64064.0 128128.0   153907

Scaled residuals:
   Min     1Q Median     3Q    Max
-7.397 -0.445 -0.366 -0.267 97.257

Random effects:
 Groups Name        Variance Std.Dev. Corr
 conv   (Intercept) 0.22947  0.4790
 sub    (Intercept) 0.16592  0.4073
        wc          0.06441  0.2538   0.22
Number of obs: 153916, groups:  conv, 3296; sub, 25

Fixed effects:
             Estimate Std. Error z value Pr(>|z|)
(Intercept) -1.585678   0.084809 -18.697  < 2e-16 ***
t           -0.096226   0.004214 -22.836  < 2e-16 ***
wc          -0.340828   0.056932  -5.987 2.14e-09 ***
prev_wc      0.394888   0.008961  44.068  < 2e-16 ***
wc:prev_wc  -0.056841   0.006911  -8.224  < 2e-16 ***
---
Signif. codes:  0 '***' 0.001 '**' 0.01 '*' 0.05 '.' 0.1 ' ' 1

Correlation of Fixed Effects:
           (Intr) t      wc     prv_wc
t          -0.154
wc          0.228 -0.014
prev_wc    -0.042  0.221 -0.020
wc:prev_wc -0.002 -0.023 -0.014 -0.181
```

We can convert the estimates of the models into odds ratios for clarity:

In [ ]:
```
%%R
se <- sqrt(diag(vcov(m_rs2)))
# table of estimates with 95% CI
tab <- cbind(Est = fixef(m_rs2), LL = fixef(m_rs2) - 1.96 * se, UL = fixef(m_rs2) + 1.96 * se)
odds_ratio = exp(tab)
probabilities = odds_ratio/(1+odds_ratio)
round(odds_ratio,2)
```

```
            Est   LL   UL
(Intercept) 0.20 0.17 0.24
t           0.91 0.90 0.92
wc          0.71 0.64 0.80
prev_wc     1.48 1.46 1.51
wc:prev_wc  0.94 0.93 0.96
```

In [ ]:
```
%%R
sjPlot::plot_model(m_rs2,axis.labels=c("word count t0 x word count t-1","word count t-1","word count t0","turn"),
                   show.values=TRUE, show.p=TRUE,
                   )
```

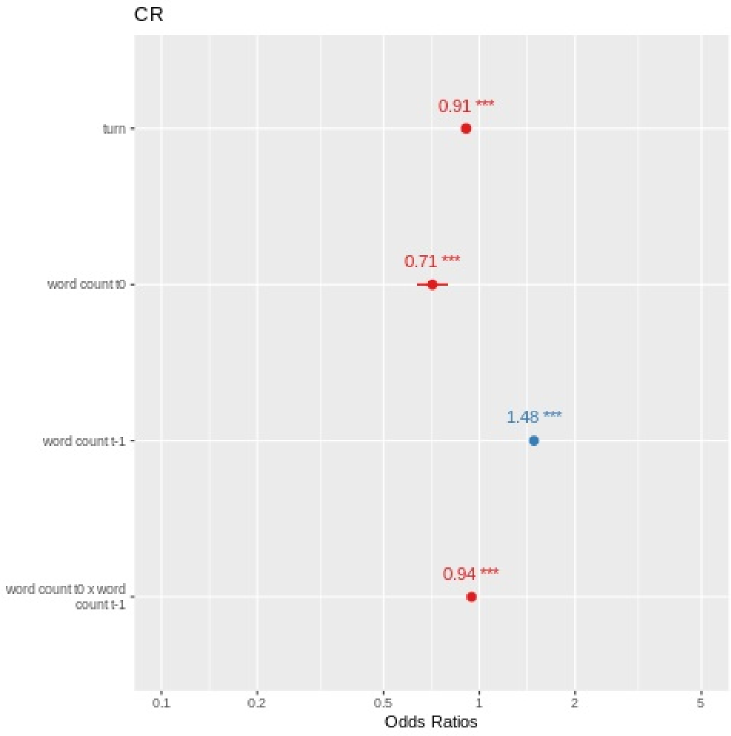

In [ ]:
```
# Run this if you want to export an easily interpreted tabular output of the model
# It provides the variance and ICC.
#%%R
#tab_model(m_rs2, file = "new_model_v11_RS6.html")
```

We can check to see whether the predicted probabilities for the subreddits by the model resemble the raw proportions.

In [ ]:
```
%%R -o sub_random_effects,intercept

fixed_effects = fixef(m_rs2)
intercept <- fixed_effects["(Intercept)"]
random_effects <- ranef(m_rs2)
sub_random_effects = random_effects$sub
```

In [ ]:
```
sub_random_effects = sub_random_effects.reset_index().rename(columns={"index":"subreddit"})
sub_weights = mme_df['sub'].value_counts().reset_index().rename(columns={"sub":"subreddit"})
sub_df5 = mme_df.groupby('sub')['CR'].agg("mean").reset_index()
sub_proportions = sub_df5[["sub","CR"]].rename(columns={"sub":"subreddit","CR":"Raw Probability"})
sub_all = pd.merge(sub_random_effects, sub_weights, on='subreddit', how='inner')
sub_all = pd.merge(sub_all, sub_proportions, on='subreddit', how='inner')
# Replicating Dingemanse and colleagues probabilities
intercept = intercept[0]
m_intercept_sub = sub_all["(Intercept)"].mean()
m_Wintercept_sub = np.average(sub_all["(Intercept)"], weights=sub_all["count"])
sub_all["Probability"] = sub_all["(Intercept)"] + intercept + m_intercept_sub + m_Wintercept_sub
sub_all["Estimated Probability"] =  1 / (1 + np.exp(-sub_all["Probability"]))
sub_all[["subreddit","Estimated Probability", "Raw Probability"]].round(2).sort_values("Raw Probability", ascending = False)
```

Out[ ]:

	subreddit	Estimated Probability	Raw Probability
9	IAmA	0.49	0.34
4	changemyview	0.26	0.19
10	jokes	0.26	0.16
24	worldnews	0.30	0.16
14	pics	0.27	0.15
18	science	0.29	0.15
17	publicfreakout	0.27	0.15
8	funny	0.21	0.14
3	brexit	0.24	0.14
21	unpopularopinion	0.20	0.14
6	explainlikeimfive	0.23	0.14
19	showerthoughts	0.21	0.14
16	psychology	0.25	0.14
5	coronavirus	0.26	0.14
22	videos	0.21	0.13
23	wallstreetbets	0.20	0.13
7	food	0.24	0.13
15	politics	0.23	0.13
12	movies	0.17	0.12
20	todayilearned	0.19	0.12
11	LifeProTips	0.14	0.11
2	books	0.12	0.10
0	askreddit	0.16	0.09
1	aww	0.17	0.09
13	music	0.13	0.09

Check the correlation between these to see if they are coherent:

In [ ]:
```
# Assuming sub_all is your DataFrame
correlation, p_value = pearsonr(sub_all["Estimated Probability"], sub_all["Raw Probability"])

print(f"Correlation: {correlation:.2f}")
print(f"P-value: {p_value:.4f}")
```

```
Correlation: 0.93
P-value: 0.0000
```

The estimated probabilities and raw proportions are highly correlated indicating the model has generalized well to the data.

2.5.1 Assumption checks

We now check the assumptions of the model by plotting the residuals.

In [ ]:
```
# Load libraries for assumption checks
%%R
library(glmmTMB)
library(DHARMa)
```

```
WARNING:rpy2.rinterface_lib.callbacks:R[write to console]: This is DHARMa 0.4.6. For overview type '?DHARMa'. For recent changes, type news(packa
ge = 'DHARMa')
```

In [ ]:
```
%%R
x = plot_model(m_rs2, grid=T, type="diag")
x[[1]]
```

```
`geom_smooth()` using formula = 'y ~ x'
```

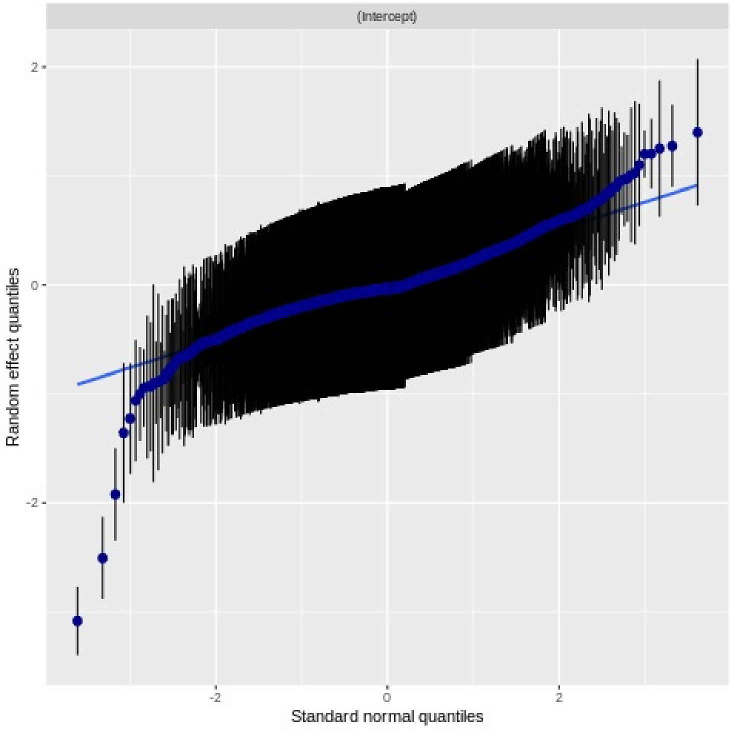

The residuals start to shift at the edges, however this is to be expected given the size of the dataset. Generally, the follow the fit line closely and so we can assume the assumptions were largely met.

2.6 Manual coding sense check

In [ ]:
```
turn_df2 = turn_df[turn_df.coded_by=="Human"]
turn_df2 = turn_df2[turn_df2.turn != 1]
turn_df2 = turn_df2[["id_turn","id_conv","subreddit","turn","initiation","word_count","prev_word_count","time_since_last_comment"]]
turn_df2 = turn_df2.rename(columns = {"subreddit":"sub",
                                    "initiation": "CR",
                                    "word_count":"wc",
                                    "prev_word_count":"prev_wc",
                                    "time_since_last_comment":"reply_t",
                                    "turn":"t",
                                    "id_conv":"conv"})
response = "CR"
fixed_effects = ["t", "wc", "prev_wc","reply_t"]
random_effects = ["sub","conv"]
mme_cols = [response] + fixed_effects + random_effects
mme_df = turn_df2[mme_cols]
# Log center the wordcount to scale the variable
scaler = StandardScaler()
mme_df[["wc","prev_wc","reply_t"]] = scaler.fit_transform(mme_df[["wc","prev_wc","reply_t"]])
```

In [ ]:
```
mme_df = mme_df.dropna()
mme_df.iteritems = mme_df.items
```

In [ ]:
```
%%R -i mme_df
data <- mme_df
```

In [ ]:
```
%%R
f_rs2  = "CR ~ 1 + t + wc + prev_wc + wc:prev_wc + (1 |conv) + (wc|sub)"
m_rs2 = glmer(f_rs2, data = data, family="binomial")
summary(m_rs2)
```

```
WARNING:rpy2.rinterface_lib.callbacks:R[write to console]: boundary (singular) fit: see help('isSingular')
```

```
Generalized linear mixed model fit by maximum likelihood (Laplace
  Approximation) [glmerMod]
 Family: binomial  ( logit )
Formula: CR ~ 1 + t + wc + prev_wc + wc:prev_wc + (1 | conv) + (wc | sub)
   Data: data

     AIC      BIC   logLik deviance df.resid 
   626.0    666.9   -304.0    608.0      687 

Scaled residuals: 
    Min      1Q  Median      3Q     Max 
-0.8154 -0.4370 -0.3868 -0.3447  3.3397 

Random effects:
 Groups Name        Variance Std.Dev. Corr 
 conv   (Intercept) 0.00000  0.0000        
 sub    (Intercept) 0.23519  0.4850        
        wc          0.09285  0.3047   -0.20
Number of obs: 696, groups:  conv, 240; sub, 25

Fixed effects:
            Estimate Std. Error z value Pr(>|z|)    
(Intercept) -1.47382    0.28466  -5.177 2.25e-07 ***
t           -0.10422    0.07710  -1.352    0.176    
wc          -0.15873    0.27203  -0.584    0.560    
prev_wc      0.08032    0.10843   0.741    0.459    
wc:prev_wc  -0.03995    0.09692  -0.412    0.680    
---
Signif. codes:  0 '***' 0.001 '**' 0.01 '*' 0.05 '.' 0.1 ' ' 1

Correlation of Fixed Effects:
           (Intr) t      wc     prv_wc
t          -0.781                     
wc          0.157  0.109              
prev_wc    -0.073  0.150  0.038       
wc:prev_wc  0.046  0.018  0.259 -0.103
optimizer (Nelder_Mead) convergence code: 0 (OK)
boundary (singular) fit: see help('isSingular')
```

3. Analysis 2: Survival analysis

In this section, we conduct a survival analyss to explore the emergence of repair initiations across subreddits. We first perform descriptive statistics relating to the emergence of initiations over time at a frequency level. We then use various survival analysis estimators to assess the increasing likelihood of a repair initiation across subredits.

Analysis 2 explores RQ2: "How does the emergence of Other-initiations vary across subreddits?"

3.1 Install and load necessary packages

In [ ]:
```
!pip install lifelines
```

In [15]:
```
import seaborn as sns
%matplotlib inline
import matplotlib.pyplot as plt
plt.rcParams['figure.dpi'] = 120
from itertools import combinations
from sklearn.preprocessing import MinMaxScaler
from lifelines import KaplanMeierFitter
from lifelines import WeibullFitter, ExponentialFitter,LogNormalFitter, LogLogisticFitter
from lifelines.plotting import qq_plot
import math
import warnings

# Suppress FutureWarning
warnings.filterwarnings("ignore", category=FutureWarning)
```

3.2 Load functions used for Analysis 2

In [16]:
```
def plot_turnCR(df, x="Interaction length", y="Interaction count"):
    # Set color palette
    colors = px.colors.qualitative.Set1

    # Create a figure for a single plot
    fig = go.Figure()

    # Add traces for the plot
```

```python
    for i, (initiation_type, group_df) in enumerate(df.groupby('initiation')):
        fig.add_trace(
            go.Bar(
                x=group_df[x],
                y=group_df[y],
                name=initiation_type,
                marker_color=colors[i % len(colors)]
            )
        )

    # Update layout
    fig.update_layout(
        barmode='group',
        xaxis_title=x,
        yaxis_title=y,
        title_text='',
        width=900,
        height=500,
        legend_title_text='Contains repair initiation',
        margin=dict(l=20, r=25, t=20, b=20)
    )
    fig.update_layout(font=dict(family="Arial", size=12))
    # Update x-axes
    fig.update_xaxes(tickmode='array', tickvals=df[x].unique())

    # Display the figure
    fig.show()


def plot_exponential_density(ax, T, E, xlabel, max_time=12, xticks=False, show_legend=True):
    exp = ExponentialFitter()
    exp.fit(T, event_observed=E, label="Exponential")

    prd = pd.DataFrame({
        "Time": exp.timeline,
        "Cumulative density": exp.cumulative_density_['Exponential']
    })

    new_row = {'Time': 0, 'Cumulative density': 0}
    prd2 = pd.concat([pd.DataFrame([new_row]), prd]).reset_index(drop=True)

    ax.plot(prd2['Time'], prd2['Cumulative density'], linestyle='--', color='blue', label='Exponential fit', linewidth=0.5)
    lower_bound = exp.confidence_interval_cumulative_density_['Exponential_lower_0.95']
    upper_bound = exp.confidence_interval_cumulative_density_['Exponential_upper_0.95']
    ax.fill_between(exp.cumulative_density_.index, lower_bound, upper_bound, alpha=0.3, color='blue')

    kmf = KaplanMeierFitter()
    kmf.fit(T, event_observed=E, label="Kaplan-Meier")
    kmf.plot_cumulative_density(ax=ax, color="grey", alpha=0.6)

    if xticks:
        ax.set_xticks(list(range(0, max_time)))
        ax.set_xticklabels(list(range(1, max_time + 1)))

    ax.set_ylabel('Cumulative Distribution')
    ax.set_xlabel(xlabel)

    if show_legend:
        ax.legend(loc='upper left', bbox_to_anchor=(0.05, -0.1), ncol=1)  # Adjust these parameters as needed
    ax.grid(True, alpha=0.5)

def plot_cumulative_densities_for_two(threads_df, conv_df, max_time1=12, max_time2=50):
    fig, axs = plt.subplots(2, 1, figsize=(7, 8))

    plot_exponential_density(axs[0], threads_df["TurnToInitiation"], threads_df.contains_initiation, "Thread length", max_time1, show_legend=True)
    axs[0].set_title("Threads")

    plot_exponential_density(axs[1], conv_df["TurnToInitiation"], conv_df.contains_initiation, "Interaction size", max_time2, show_legend=False)
    axs[1].set_title("Interactions")

    plt.rcParams['font.family'] = 'Liberation Sans'
    plt.rcParams['font.size'] = 10
    plt.tight_layout()

def plot_median_survival(threads_df, conv_df):
    def prepare_data(sub_df, category_name):
        """Helper function to prepare data for plotting."""
        sub_df.columns = ["Subreddit", "Median Survival Time"]
        sub_df["Category"] = category_name
        sub_df["Rank"] = sub_df["Median Survival Time"].rank(method='first', ascending=True)
        return sub_df

    # Prepare data for each category
```

```python
    conv_data = prepare_data(conv_df, "Interactions")
    turn_data = prepare_data(threads_df, "Threads")
    # Check for and handle infinite values
    conv_data.replace([np.inf, -np.inf], 11, inplace=True)
    turn_data.replace([np.inf, -np.inf], 11, inplace=True)
    # Combine dataframes
    combined_df = pd.concat([conv_data, turn_data])

    # Add initiation rate from interactions for color mapping
    color_mapping_conv = conv_data.set_index("Subreddit")["Median Survival Time"].to_dict()
    color_mapping_turn = turn_data.set_index("Subreddit")["Median Survival Time"].to_dict()

    # Normalize initiation rates for coloring
    scaler = MinMaxScaler(feature_range=(0, 1))
    normalized_conv = scaler.fit_transform(conv_data[["Median Survival Time"]])
    normalized_turn = scaler.fit_transform(turn_data[["Median Survival Time"]])

    # Update color mappings with normalized values
    normalized_color_mapping_conv = dict(zip(conv_data["Subreddit"], normalized_conv.flatten()))
    normalized_color_mapping_turn = dict(zip(turn_data["Subreddit"], normalized_turn.flatten()))

    # Create bump chart
    fig = px.line(combined_df, x="Category", y="Rank", color="Subreddit",
                  line_shape='linear', markers=True,
                  labels={"Rank": "Rank", "Category": "Category"},
                  width=800,  # Set width suitable for Word
                  height=600) # Set height suitable for Word

    # Update trace colors based on normalized initiation rate for interactions
    for trace in fig.data:
        subreddit = trace.name
        initiation_rate = normalized_color_mapping_conv.get(subreddit, 0)  # Get normalized initiation rate
        # Map initiation rate to color using Viridis scale
        line_color = px.colors.sample_colorscale('Viridis', initiation_rate)[0]  # Get the first element from the list
        trace.line.color = line_color
        trace.marker.color = line_color  # Make sure markers have the same color

    # Invert y-axis to have rank 1 at the top
    fig.update_yaxes(autorange="reversed", dtick=1)  # Add individual ticks for each rank

    # Remove legend
    fig.update_layout(showlegend=False)

    # Add annotations for each subreddit at the leftmost position
    for subreddit in combined_df["Subreddit"].unique():
        # Get the first point for each subreddit
        first_point = combined_df[combined_df["Subreddit"] == subreddit].iloc[0]
        last_point = combined_df[(combined_df["Subreddit"] == subreddit) & (combined_df["Category"] == "Threads")].iloc[0]
        initiation_rate_conv = color_mapping_conv[subreddit]
        initiation_rate_turn = color_mapping_turn[subreddit]

        # Set the annotation x position slightly to the left of the first point
        annotation_x_pos = -0.05  # Use a fixed offset to the left of the first category

        fig.add_annotation(
            x=annotation_x_pos,
            y=first_point["Rank"],
            text=f"{subreddit} ({initiation_rate_conv:.0f} turns)",  # Add initiation rate percentage from Interactions
            showarrow=False,
            xanchor="right",  # Position the text to the left of the point
            yanchor="middle",
            xshift=-5  # Additional shift to the left
        )

        # Add annotation for each subreddit at the rightmost position (Turns)
        annotation_x_pos_right = 1.05  # Adjusted for only two categories
        fig.add_annotation(
            x=annotation_x_pos_right,
            y=last_point["Rank"],
            text=f"{subreddit} ({initiation_rate_turn:.0f} turns)",  # Add initiation rate percentage from Turns
            showarrow=False,
            xanchor="left",  # Position the text to the right of the point
            yanchor="middle",
            xshift=5  # Additional shift to the right
        )
    fig.update_layout(font=dict(family="Arial", size=12))
    fig.update_layout(height=1000, width=800)
    fig.update_xaxes(title="")
    # Show plot
    fig.show()

def convertHazard_to_density(cum_hazard, return_survival=False):
  """
```

```
  Calculates the cumulative density from the cumulative hazard (e^(-Hazard))
  """
  survival = math.exp(-cum_hazard)
  if return_survival:
    return survival
  else:
    return 1 - survival

def get_cumulative_density(model, max_time = 36, return_survival=False):
  """
  Iterate over time points and calculate cumulative density
  """
  times = list(range(1,max_time))
  dens_lst = []
  for t in times:
    cum_hazard = model.cumulative_hazard_at_times(t)
    dens_ = convertHazard_to_density(cum_hazard, return_survival)
    dens_lst.append(dens_)
  return pd.DataFrame({"Time":times,"Cumulative density":dens_lst})


def sub_exp_density(surv_df, time_col, ax, x_label='Turn'):
    sub_df = surv_df.groupby("subreddit").agg({"contains_initiation": "mean"}).reset_index()
    sub_df = sub_df.sort_values("contains_initiation", ascending=False)
    sub_order = sub_df["subreddit"].to_list()
    exp = ExponentialFitter()
    T = surv_df[time_col]
    E = surv_df.contains_initiation
    mxtime = int(max(T))
    colors = list(reversed(sns.color_palette("Blues", len(surv_df["subreddit"].unique()))))
    for i, sub in enumerate(sub_order):
        T_temp = surv_df[surv_df["subreddit"] == sub][time_col]
        E_temp = surv_df[surv_df["subreddit"] == sub].contains_initiation
        exp.fit(T_temp, E_temp, label=sub)
        exp.cumulative_density_.plot(ax=ax, color=colors[i])
        prd = get_cumulative_density(exp, max_time=mxtime)
        new_row = {'Time': 0, 'Cumulative density': 0}
        prd2 = pd.concat([pd.DataFrame([new_row]), prd]).reset_index(drop=True)
        ax.plot(prd2['Time'], prd2['Cumulative density'], linestyle='--', color=colors[i], label='', linewidth=0.5)

    kmf = KaplanMeierFitter()
    kmf.fit(T, E, label="Kaplan-Meier")
    kmf.plot_cumulative_density(ax=ax, loc=slice(0, mxtime), color="grey", alpha=0.6)
    custom_ticks_y = np.arange(0, 1.1, 0.1)
    ax.set_yticks(custom_ticks_y)
    ax.legend(loc='center left', bbox_to_anchor=(1, 0.5), ncol=2)
    ax.set_xlabel(x_label)
    ax.set_ylabel('Cumulative density')
    ax.grid(True, alpha=0.5)

def combined_plot(surv_df1, surv_df2, time_col, x_label1='Turn', x_label2='Turn'):
    fig, axs = plt.subplots(nrows=2, ncols=1, figsize=(9, 8))
    sub_exp_density(surv_df1, time_col, axs[0], x_label=x_label1)
    sub_exp_density(surv_df2, time_col, axs[1], x_label=x_label2)
    plt.rcParams['font.family'] = 'Liberation Sans' # as alternative to Arial
    plt.rcParams['font.size'] = 10
    plt.tight_layout()
    plt.show()

def plot_cumulative_density(threads_df, conv_df):
    fig, axs = plt.subplots(2, 1, figsize=(7, 7))

    # Plot for the first dataframe
    kmf = KaplanMeierFitter()
    ax = axs[0]
    baseline_T = threads_df["TurnToInitiation"]
    baseline_E = threads_df.contains_initiation
    kmf.fit(baseline_T, baseline_E, label="N turns until initiation")
    kmf.plot_cumulative_density(ax=ax, color="black", at_risk_counts=False)
    ax.set_title("Threads: Turn-to-initiation")
    print(kmf.median_survival_time_)
    # Plot for the second dataframe
    kmf = KaplanMeierFitter()
    ax = axs[1]
    baseline_T = conv_df["TurnToInitiation"]
    baseline_E = conv_df.contains_initiation
    kmf.fit(baseline_T, baseline_E, label="N turns until initiation")
    kmf.plot_cumulative_density(ax=ax, color="black", at_risk_counts=False)
    ax.set_title("Interactions: Turn-to-initiation")
    print(kmf.median_survival_time_)
    plt.rcParams['font.family'] = 'Liberation Sans' # as alternative to Arial
    plt.rcParams['font.size'] = 10
    plt.tight_layout()
```

```
plt.show()
```

3.3 Descriptive statistics

We perform descriptive statistics at both the threads and interaction level of analysis.

3.3.1 Thread level descriptives

In [17]:
```
dia_len_desc = threads_df.interaction_length.describe()
dL_m = dia_len_desc.loc["mean"]
dL_median = dia_len_desc.loc["50%"]
dL_std = dia_len_desc.loc["std"]
dL_max = dia_len_desc.loc["max"]
print(f"The median number of turns in each thread is small (Median={dL_median}). The distribution of thread length is highly skewed (M={dL_m:.2f}; SD
```

The median number of turns in each thread is small (Median=3.0). The distribution of thread length is highly skewed (M=3.60; SD=2.03; Min = 1; Max = 1
1.0)

We can plot the initiations across the dataset. This helps us visualize how the distribution of repair initiations varies for different interaction lengths. The longer the interaction, the higher ratio of repairs.

In [18]:
```
surv_df = threads_df.copy()
surv_initiation = surv_df[surv_df.contains_initiation != 0]
surv_NOinitiation = surv_df[surv_df.contains_initiation == 0]
s_i =  surv_initiation.groupby('interaction_length')['contains_initiation'].agg(['sum']).reset_index()
s_Ni =  surv_NOinitiation.groupby('interaction_length')['contains_initiation'].agg(['count']).reset_index()
s_i = s_i.rename(columns = {"sum": "count"})
s_i["initiation"] = "Yes"
s_Ni["initiation"] = "No"
s_all = pd.concat([s_i, s_Ni])
s_all = s_all.rename(columns = {"interaction_length":"Thread length","count":"Thread count"})
plot_turnCR(s_all,x="Thread length",y="Thread count")
```

In [26]:
```
Image('Thread_length_time.png')
```

Out[26]:

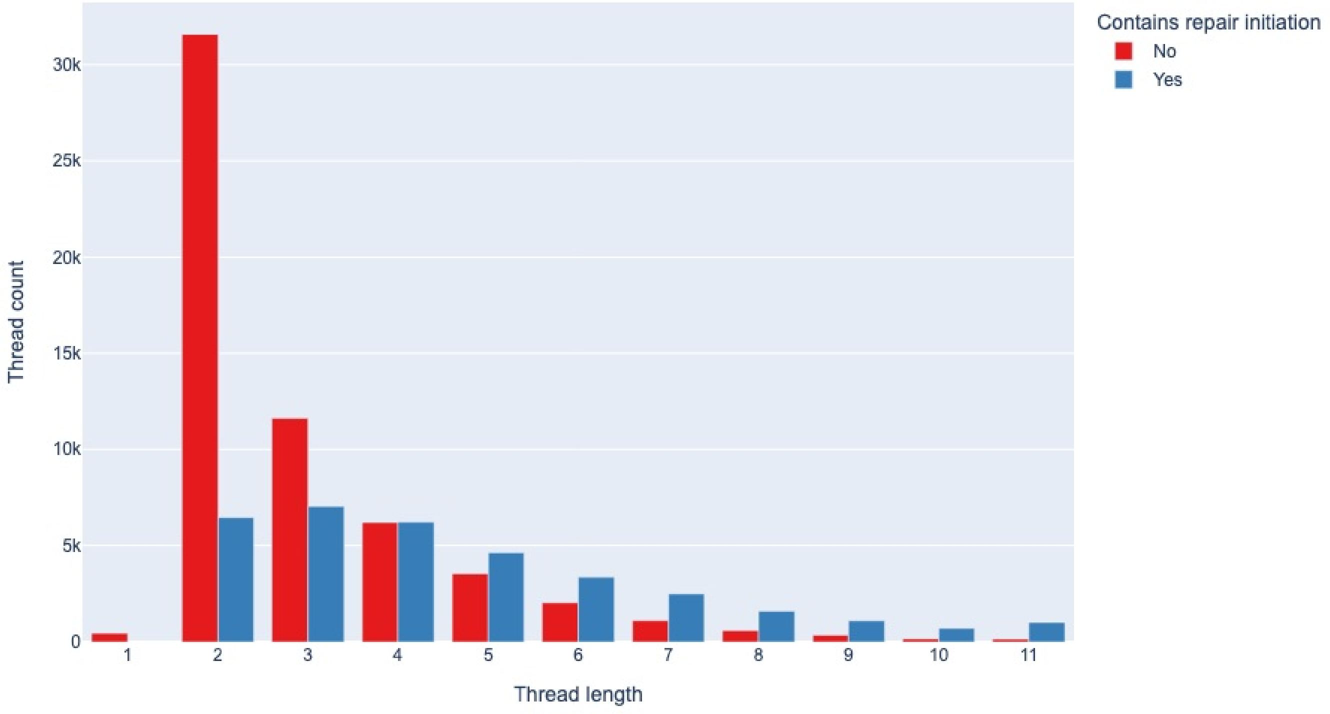

In [ ]:

```
total_interactions = s_all["Thread count"].sum()
FewerThan_3_turns = s_all[s_all["Thread length"] < 4]["Thread count"].sum()
ratio =  FewerThan_3_turns /total_interactions * 100
print(f"""
Of {total_interactions} threads, {FewerThan_3_turns} ({ratio:.2f}%) are <= 3 turns long.
""")
```

Of 92441 threads, 57170 (61.84%) are <= 3 turns long.

At the subreddit level we can examine the average interaction size and thread length to examine variations between subreddits:

In [19]:

```
subreddits = conv_df.subreddit.unique()
average_length = []
average_size = []
for sub in subreddits:
  average_length.append(threads_df[threads_df.subreddit == sub].interaction_length.median())
  average_size.append(conv_df[conv_df.subreddit == sub].interaction_size.median())
subreddit_length = pd.DataFrame({"subreddit":subreddits, "Median thread length":average_length,"Median interaction size":average_size}).round(2)
subreddit_length.sort_values(by=["Median interaction size"],ascending=False)
```

Out[19]:

	subreddit	Median thread length	Median interaction size
14	IAmA	4.0	87.5
11	changemyview	4.0	42.0
9	politics	3.0	34.0
23	publicfreakout	3.0	29.0
15	wallstreetbets	2.0	23.0
5	brexit	3.0	18.0
19	movies	3.0	17.5
20	todayilearned	3.0	17.0
6	askreddit	2.0	17.0
16	unpopularopinion	2.0	17.0
7	books	2.0	11.0
8	coronavirus	4.0	9.5
10	worldnews	4.0	8.0
0	funny	2.0	7.0
12	LifeProTips	3.0	7.0
4	showerthoughts	2.0	7.0
17	explainlikeimfive	3.0	6.0
21	jokes	3.0	4.0
2	pics	3.0	4.0
24	science	3.0	4.0
3	aww	2.0	3.5
13	food	2.0	3.0
22	psychology	2.0	3.0
18	videos	3.0	2.0
1	music	2.0	1.0

3.3.2 Interaction level descriptives

We perform the same statistics for the interaction level data.

In [20]:

```
dia_s_desc = conv_df.interaction_size.describe()
dL_m = dia_s_desc.loc["mean"]
dL_median = dia_s_desc.loc["50%"]
dL_std = dia_s_desc.loc["std"]
dL_max = dia_s_desc.loc["max"]
print(f"The median number of comments for each post is small (Median={dL_median}). The distribution of interaction sizes is highly skewed (M={dL_m:..
```

The median number of comments for each post is small (Median=9.0). The distribution of interaction sizes is highly skewed (M=42.04; SD=94.53; Min = 1 ; Max = 501.0)

In [21]:

```
# truncate the data due to long tail
size_ = 50
surv_df = conv_df.copy()
surv_initiation = surv_df.loc[(surv_df['contains_initiation'] != 0) & (surv_df['interaction_size'] < size_)]
surv_NOinitiation = surv_df.loc[(surv_df['contains_initiation'] == 0) & (surv_df['interaction_size'] < size_)]
s_i =  surv_initiation.groupby('interaction_size')['contains_initiation'].agg(['sum']).reset_index()
s_Ni =  surv_NOinitiation.groupby('interaction_size')['contains_initiation'].agg(['count']).reset_index()
s_i = s_i.rename(columns = {"sum": "count"})
s_i["initiation"] = "Yes"
s_Ni["initiation"] = "No"
s_all = pd.concat([s_i, s_Ni])
s_all = s_all.rename(columns = {"interaction_size":"Interaction size","count":"Interaction count"})
plot_turnCR(s_all,x="Interaction size",y="Interaction count")
```

In [27]:
```
Image('Interaction_size_time.png')
```

Out[27]:

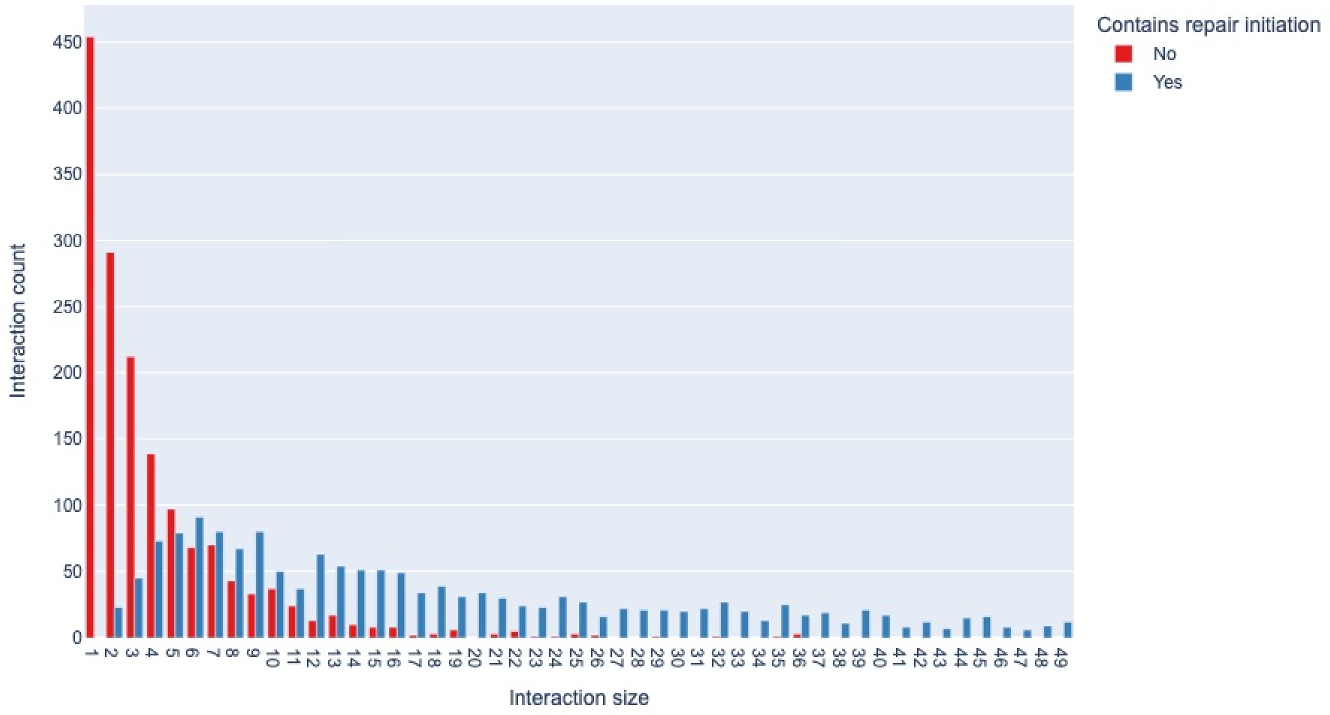

In [ ]:
```
total_interactions = s_all["Interaction count"].sum()
FewerThan_3_turns = s_all[s_all["Interaction size"] < 11]["Interaction count"].sum()
ratio =  FewerThan_3_turns /total_interactions * 100
print(f"""
Of {total_interactions} threads, {FewerThan_3_turns} ({ratio:.2f}%) are <= 10 turns long.
""")
```

Of 3107 threads, 2032 (65.40%) are <= 10 turns long.

3.4 Survival analysis

From the distribution plots, it appears that the likelihood of a repair initiation increases as a thread grows in length and an interaction grows in size. To plot this we can use the Kaplan-Meier estimator [8], a non-parametric method for identifying the survival function. To make the graphs clearer we plot the Cumulative Distribution Function (CDF) which equates to 1 - the survival function (S(t)) estimated using the Kaplan-Meier.

In [22]:
```
plot_cumulative_density(threads_df, conv_df)
```

5.0
6.0

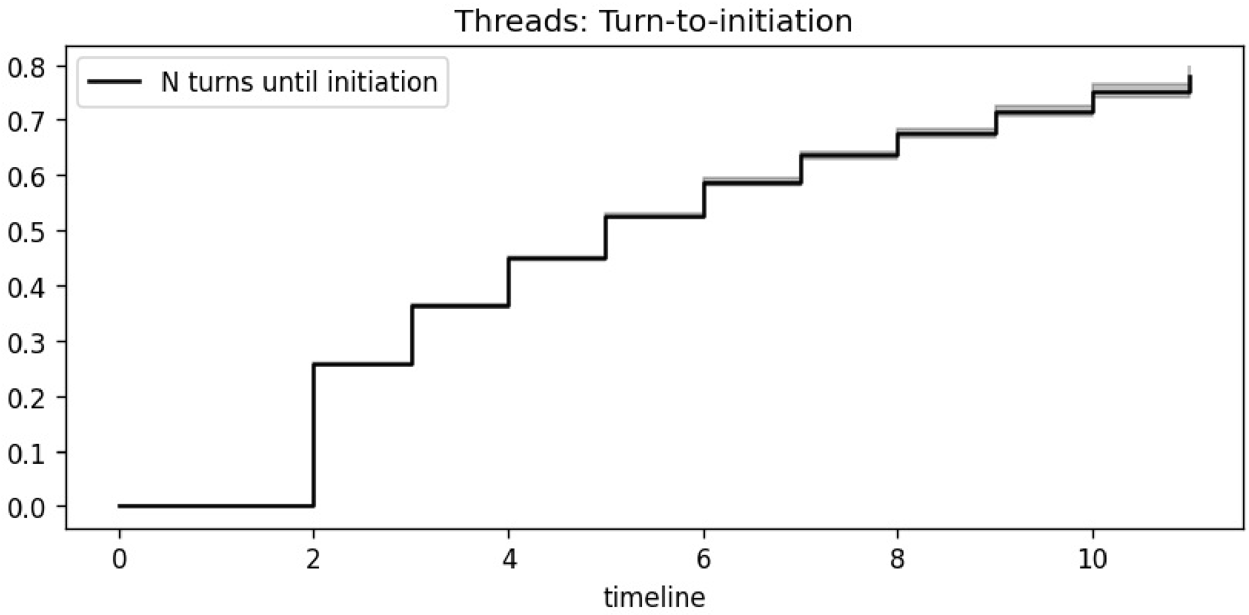

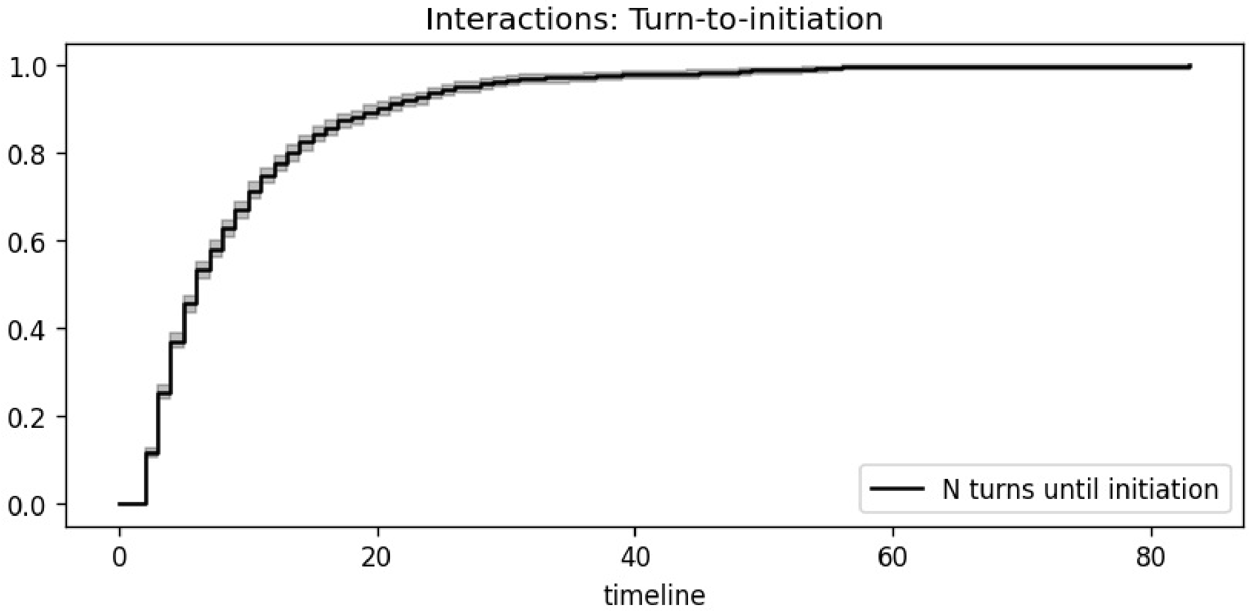

We observe that the cumulative distributions increase as the threads grow in length (top graph) and the interactions grow in size (bottom graph).

3.4.1 Identifying a parametric model

To be able to see the rate of growth, we can apply a parametric model. The below seeks to find the best model for the data at the interaction level:

In [ ]:

```
T = conv_df["TurnToInitiation"]
E = conv_df["contains_initiation"]
fig, axes = plt.subplots(2, 2, figsize=(8, 6))
axes = axes.reshape(4,)
AIC_ = []
BIC_ = []
models = []
Names_ = ["Weibull","LogNormal","LogLogistic","Exponential"]
for i, model in enumerate([WeibullFitter(), LogNormalFitter(), LogLogisticFitter(), ExponentialFitter()]):
    model.fit(T, E)
    qq_plot(model, ax=axes[i])
    AIC_.append(model.AIC_)
    BIC_.append(model.BIC_)
    models.append(model)
AICs = pd.DataFrame({"Fitter":Names_,"AIC":AIC_,"BIC":BIC_}).round(2)
AICs
```

Out[ ]:

	Fitter	AIC	BIC
**0**	Weibull	13943.33	13955.79
**1**	LogNormal	13245.95	13258.41
**2**	LogLogistic	13380.87	13393.32
**3**	Exponential	14307.93	14314.16

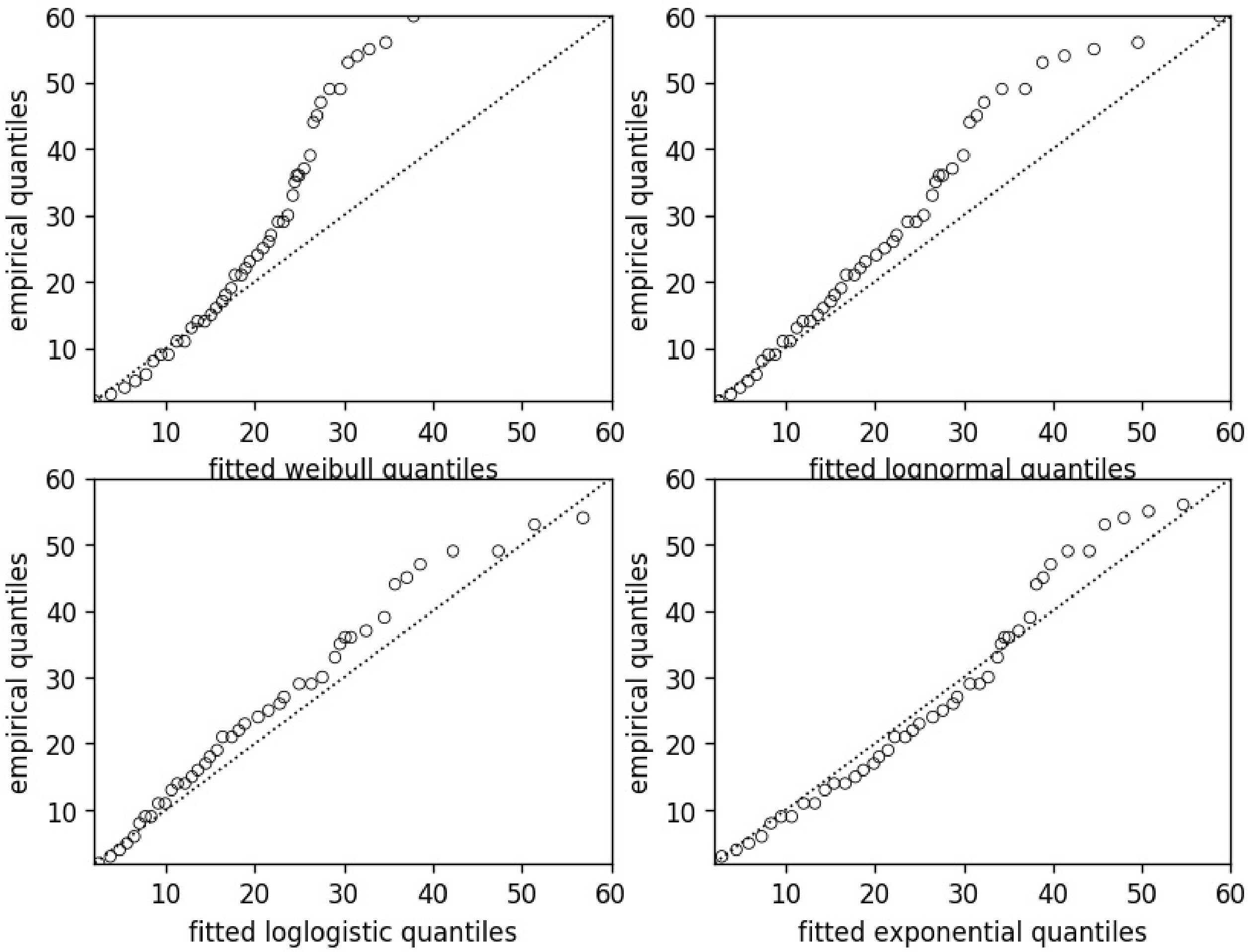

While the Weibull has the lowest AIC and exponential fitter the highest. However, we can see that only the exponential quantile plot shows the best fit (closest to the fit line). We can examine the results of the model as follows:

In [ ]:

```
exp = models[3]
exp.print_summary()
```

**model**	lifelines.ExponentialFitter
**number of observations**	3750
**number of events observed**	2193
**log-likelihood**	-7152.96
**hypothesis**	lambda_ != 0

	coef	se(coef)	coef lower 95%	coef upper 95%	cmp to	z	p	-log2(p)
**lambda_**	9.60	0.20	9.20	10.00	0.00	46.83	<0.005	inf

AIC 14307.93

We repeat the same but for the threads this time.

In [ ]:

```
T = threads_df["TurnToInitiation"]
E = threads_df["contains_initiation"]
fig, axes = plt.subplots(2, 2, figsize=(8, 6))
axes = axes.reshape(4,)
AIC_ = []
BIC_ = []
models = []
```

```
Names_ = ["Weibull","LogNormal","LogLogistic","Exponential"]
for i, model in enumerate([WeibullFitter(), LogNormalFitter(), LogLogisticFitter(), ExponentialFitter()]):
    model.fit(T, E)
    qq_plot(model, ax=axes[i])
    AIC_.append(model.AIC_)
    BIC_.append(model.BIC_)
    models.append(model)
AICs = pd.DataFrame({"Fitter":Names_,"AIC":AIC_,"BIC":BIC_}).round(2)
AICs
```

Out[ ]:

	Fitter	AIC	BIC
**0**	Weibull	187756.94	187775.81
**1**	LogNormal	173396.86	173415.73
**2**	LogLogistic	178045.79	178064.66
**3**	Exponential	209925.12	209934.55

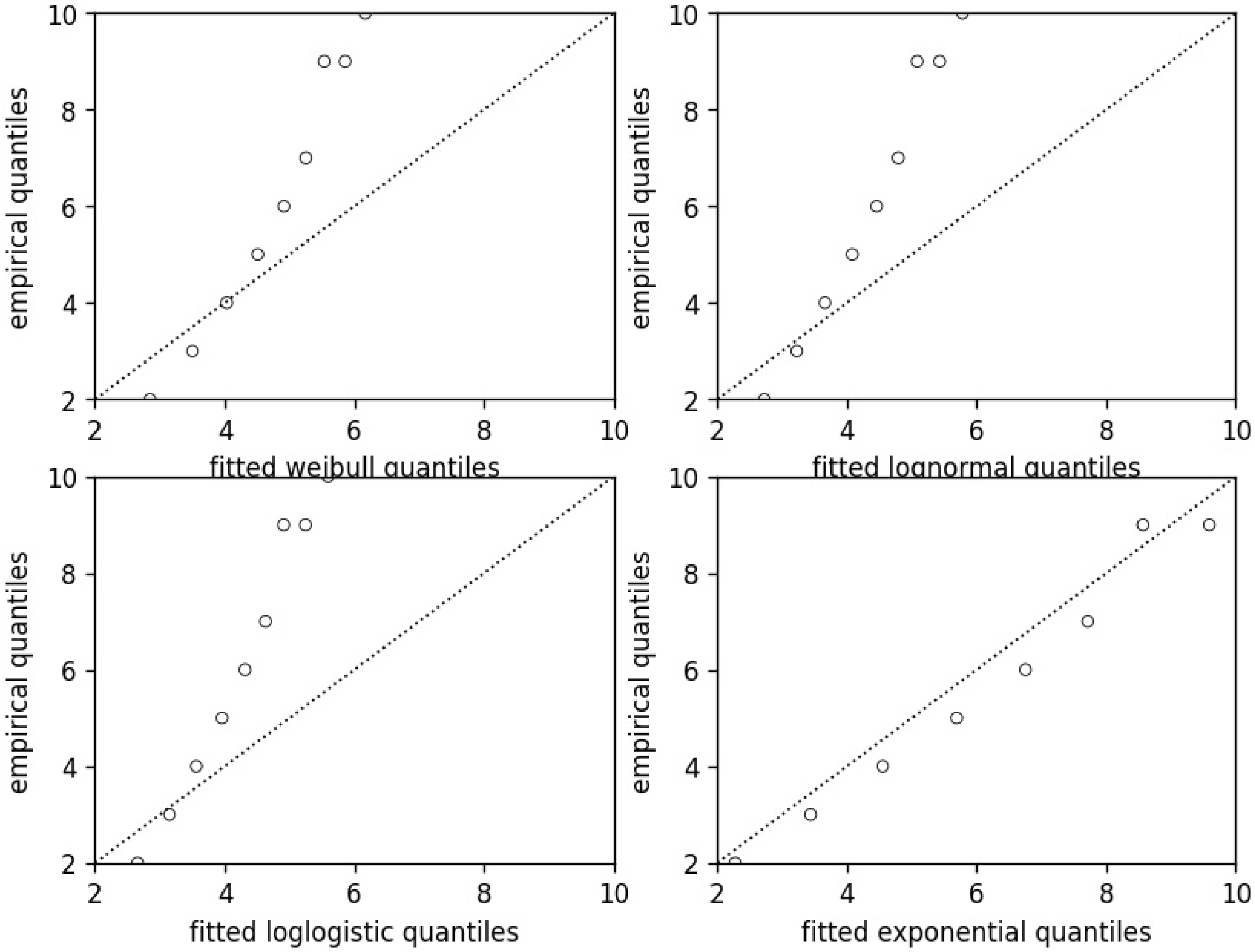

We see the same pattern as with the interaction level data, where the exponential quantiles appear to show the best fit to the data.

In [ ]:

```
exp = models[3]
exp.print_summary()
```

**model**	lifelines.ExponentialFitter
**number of observations**	92441
**number of events observed**	34642
**log-likelihood**	-104961.56
**hypothesis**	lambda_ != 0

	coef	se(coef)	coef lower 95%	coef upper 95%	cmp to	z	p	-log2(p)
**lambda_**	7.61	0.04	7.53	7.69	0.00	186.12	<0.005	inf

AIC 209925.12

In both cases, the exponential fitter works best for the data. We plot these alongside the graph:

In [23]:
```
plot_cumulative_densities_for_two(threads_df, conv_df)
```

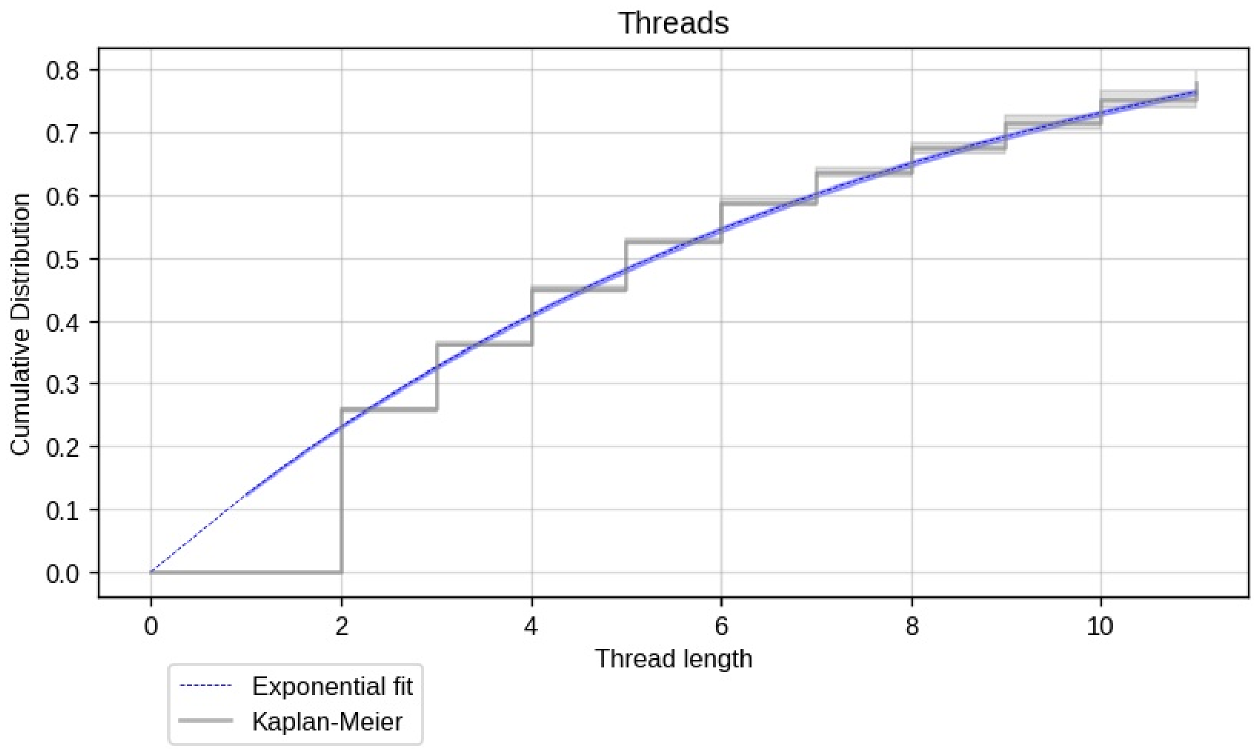

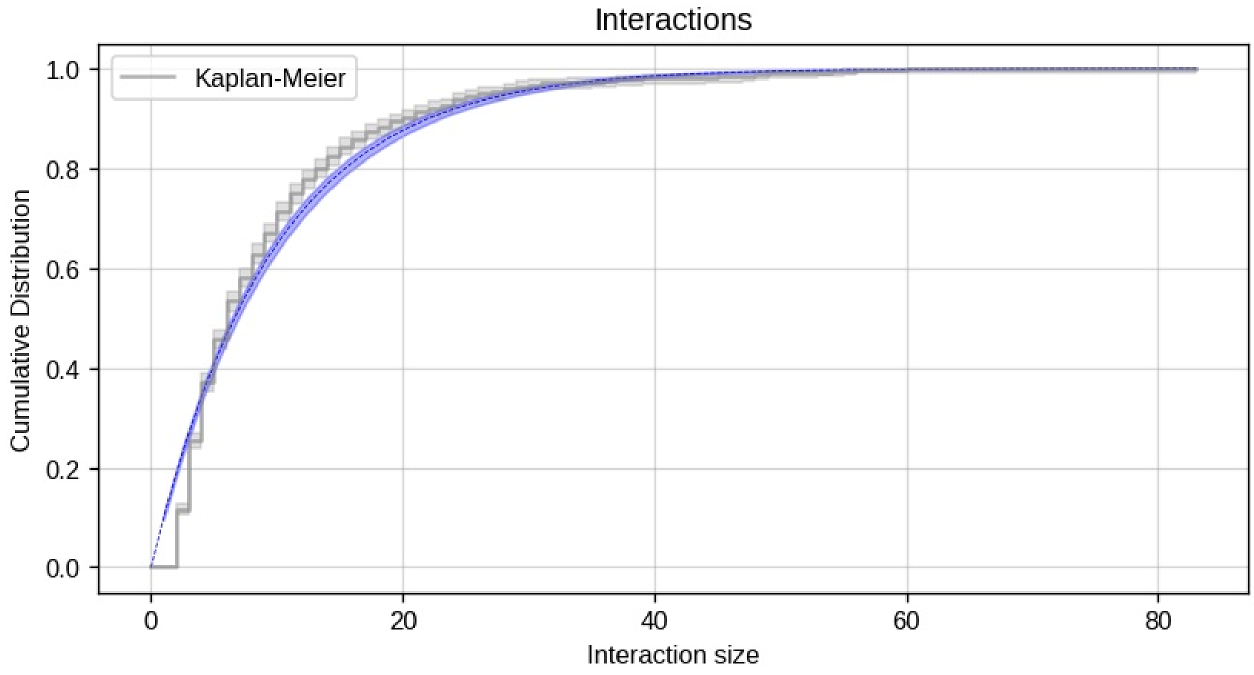

3.4.2 Comparing subreddits survival times

We are interested in the different survival times for the subreddits to address RQ2. We can initially plot these in a similar fashion to how we did it across the whole dataset. We use the exponential fitter as this provides a better visual representation of the data than the Kaplan-Meier curves alone:

In [ ]:
```
combined_plot(threads_df, conv_df, "TurnToInitiation", "Thread length", "Interaction size")
```

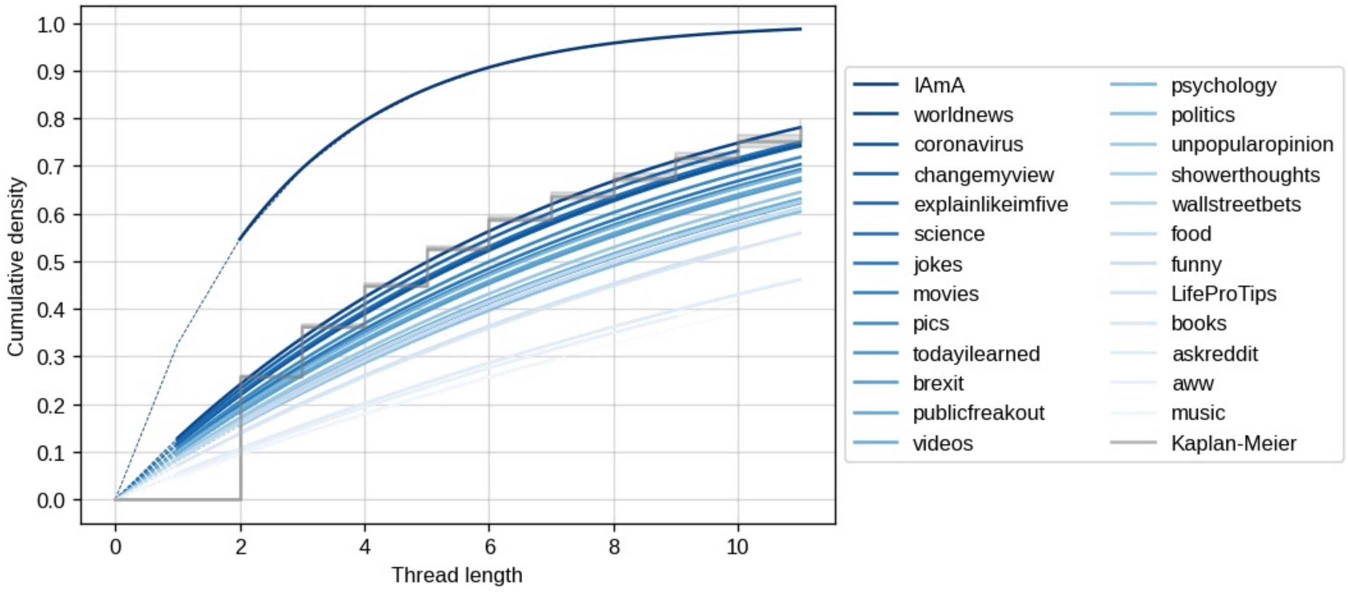

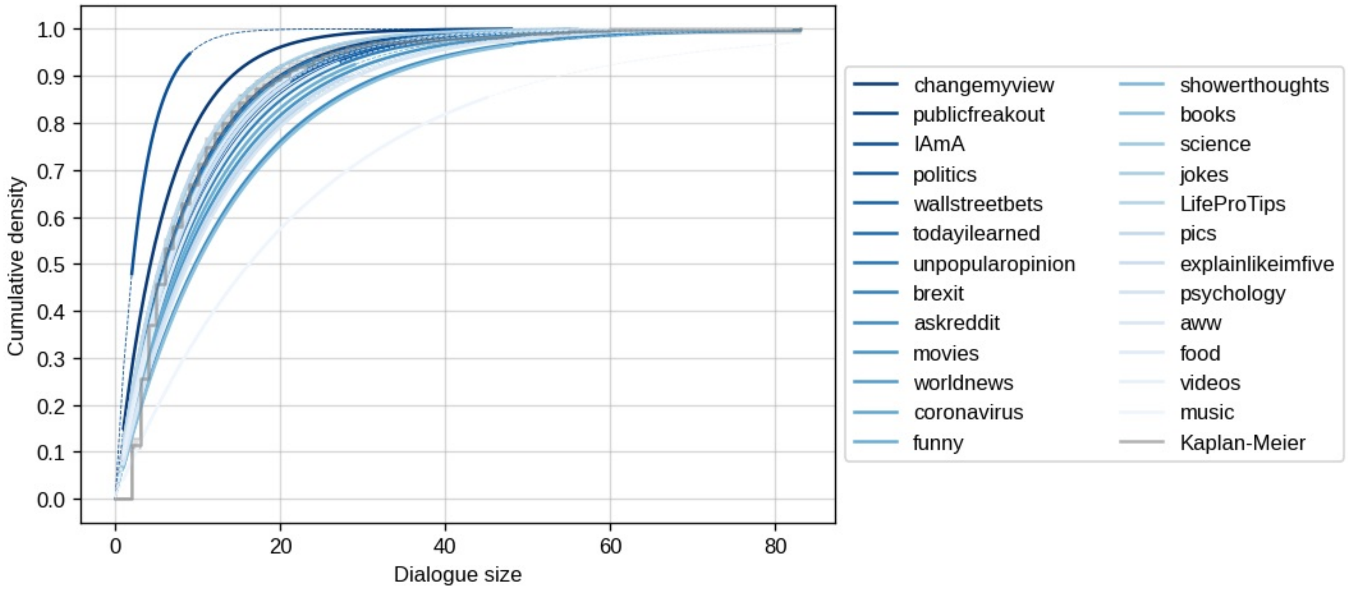

We can see from the different graphs that the subreddits vary in terms of there rank for the survival times across threads and interactions. We can see these ranks explicitly by plotting them. In this case the median survival times are given for each subreddit. These are calculated using the Kaplan-Meier estimator:

In [24]:
```
kmf = KaplanMeierFitter()

mst_conv = []
mst_thread = []
for i, sub in enumerate(conv_df.subreddit.unique()):
    T_conv = conv_df[conv_df["subreddit"] == sub].TurnToInitiation
    E_conv = conv_df[conv_df["subreddit"] == sub].contains_initiation
    kmf.fit(T_conv, E_conv, label=sub)
    mst_conv.append(kmf.median_survival_time_)
    T_thread = threads_df[threads_df["subreddit"] == sub].TurnToInitiation
    E_thread = threads_df[threads_df["subreddit"] == sub].contains_initiation
    kmf.fit(T_thread, E_thread, label=sub)
    mst_thread.append(kmf.median_survival_time_)
mst_dfconv = pd.DataFrame({"Subreddit":conv_df.subreddit.unique(), "Median Survival Time":mst_conv})
mst_dfthread = pd.DataFrame({"Subreddit":conv_df.subreddit.unique(), "Median Survival Time":mst_thread})
plot_median_survival(mst_dfthread,mst_dfconv)
```

In [28]:

```
Image('Initiation_survival_ranks.png')
```

Out[28]:

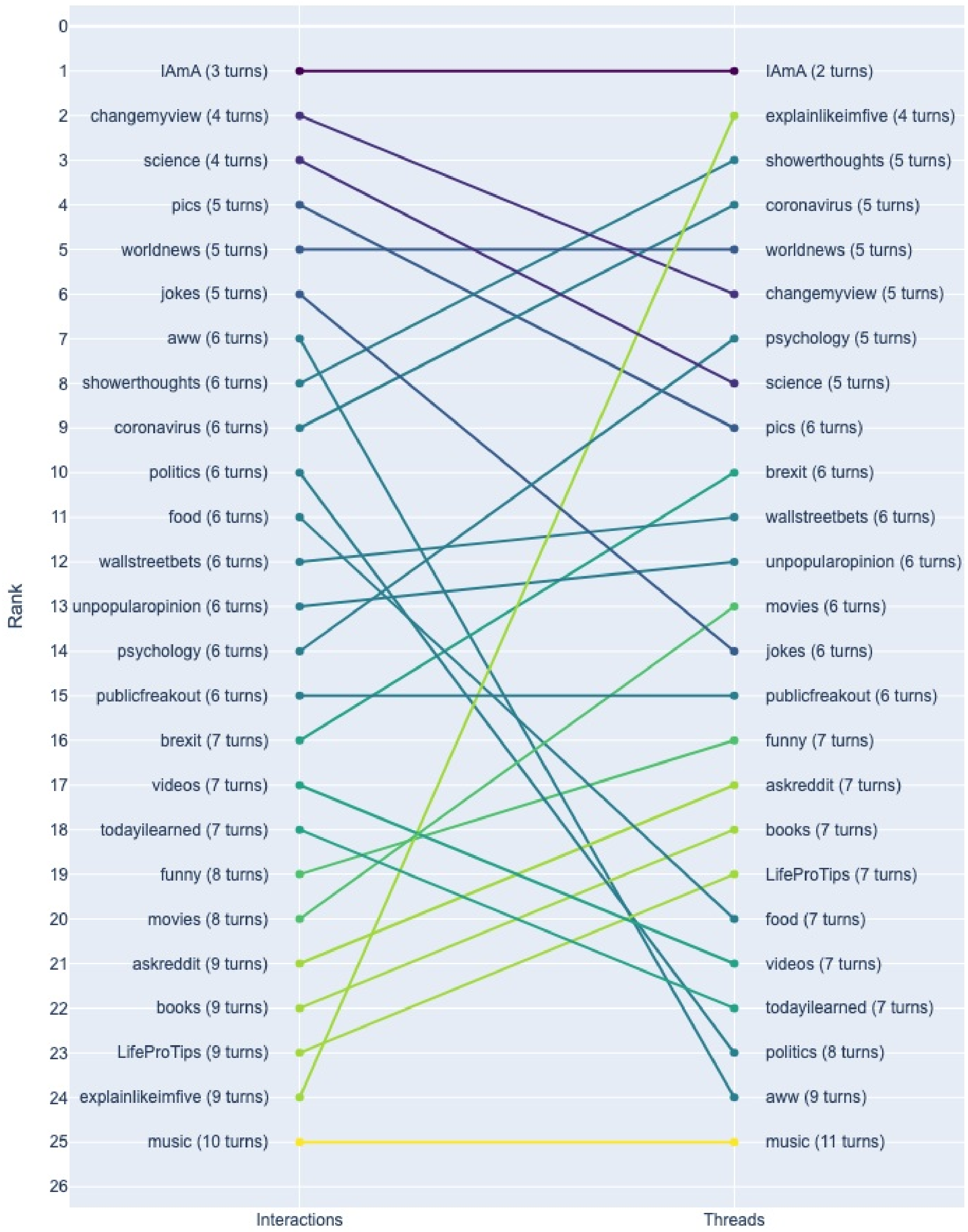

References

1. Devlin J, Chang M-W, Lee K, Toutanova K. BERT: Pre-training of deep bidirectional transformers for language understanding. Proceedings of the 2019 Conference of the North American Chapter of the Association for Computational Linguistics: Human Language Technologies, Volume 1 (Long and Short Papers). Minneapolis, Minnesota: Association for Computational Linguistics; 2019. pp. 4171–4186. doi:10.18653/v1/N19-1423

2. Maiya AS. ktrain: A low-code library for augmented machine learning. arXiv; 2022. doi:10.48550/arXiv.2004.10703

3. Bates D, Maechler M, Bolker B, Walker S. lme4: Linear Mixed-Effects Models using “Eigen” and S4. 2003. p. 1.1-35.5. doi:10.32614/CRAN.package.lme4

4. Davidson-Pilon C. lifelines: survival analysis in Python. Journal of Open Source Software. 2019;4: 1317. doi:10.21105/joss.01317

5. Dingemanse M, Kendrick KH, Enfield NJ. A coding scheme for other-initiated repair across languages. Open Linguistics. 2016;2: 35–46. doi:10.1515/opli-2016-0002

6. Dingemanse M, Roberts SG, Baranova J, Blythe J, Drew P, Floyd S, et al. Universal principles in the repair of communication problems. PLOS ONE. 2015;10: e0136100. doi:10.1371/journal.pone.0136100

7. Heisig JP, Schaeffer M. Why You Should Always Include a Random Slope for the Lower-Level Variable Involved in a Cross-Level Interaction. European Sociological Review. 2019;35: 258–279. doi:10.1093/esr/jcy053

8. Kaplan EL, Meier P. Nonparametric estimation from incomplete observations. Journal of the American Statistical Association. 1958;53: 457–481.
